# A phase 3, multicenter, double-blind, randomized, placebo-controlled clinical trial to verify the efficacy and safety of ansofaxine (LY03005) for major depressive disorder

**DOI:** 10.1038/s41398-023-02435-0

**Published:** 2023-05-10

**Authors:** Weifeng Mi, Xiaolan Di, Yiming Wang, Huafang Li, Xiufeng Xu, Lehua Li, Huaning Wang, Guoqiang Wang, Kerang Zhang, Feng Tian, Jiong Luo, Chanjuan Yang, Yunfei Zhou, Shiping Xie, Hua Zhong, Bin Wu, Dong Yang, Zhenhua Chen, Yi Li, Jindong Chen, Shuyun Lv, Qizhong Yi, Zhiwei Jiang, Jingwei Tian, Hongyan Zhang

**Affiliations:** 1 Peking University Sixth Hospital, Peking University Institute of Mental Health, NHC Key Laboratory of Mental Health (Peking University), National Clinical Research Center for Mental Disorders (Peking University Sixth Hospital), Beijing, China; 2 Beijing Huilongguan Hospital, Beijing, China; 3 The Affiliated Hospital of Guizhou Medical University, Guizhou, China; 4 Shanghai Mental Health Center, Shanghai, China; 5 First Affiliated Hospital of Kunming Medical University, Kunming, China; 6 Second Xiangya Hospital of Central South University, Changsha, China; 7 First Affiliated Hospital of the Fourth Military Medical University of Chinese People’s Liberation Army, Xi’an, China; 8 Wuxi Mental Health Center, Wuxi, China; 9 First Hospital of Shanxi Medical University, Taiyuan, China; 10 Second Hospital of Shanxi Medical University, Taiyuan, China; 11 Beijing Anding Hospital of Capital Medical University, Beijing, China; 12 The Affiliated Brain Hospital of Guangzhou Medical University, Guangzhou, China; 13 Shenzhen Kangning Hospital, Shenzhen, China; 14 Nanjing Brain Hospital, Nanjing, China; 15 Huzhou Third Municipal Hospital, Huzhou, China; 16 Xi ‘an Mental Health Center, Xi’an, China; 17 Hunan Brain Hospital, Changsha, China; 18 Renmin Hospital of Wuhan University, Wuhan, China; 19 Wuhan Mental Health Center, Wuhan, China; 20 Xiamen Xianyue Hospital, Xiamen, China; 21 The Fourth People Hospital of Urumqi, Urumqi, China; 22 The First Affiliated Hospital of Xinjiang Medical University, Urumqi, China; 23 Beijing KeyTech Statistical Technology Co., Ltd, Beijing, China; 24 Yantai University, Yantai, China

**Keywords:** Depression, Clinical pharmacology

## Abstract

Major depressive disorder (MDD) is the most prevalent form of depression and is becoming a great challenge for public health and medical practice. Although first-line antidepressants offer therapeutic benefits, about 35% of depressed patients are not adequately treated, creating a substantial unmet medical need. A multicenter, double-blind, randomized, placebo-controlled phase 3 clinical trial was conducted in patients with MDD in China to assess the efficacy and safety of ansofaxine (LY03005), a potential triple reuptake inhibitor of serotonin, norepinephrine, and dopamine. Eligible 588 MDD patients were included and randomly assigned (1:1:1) to 8-week treatment with ansofaxine 80 mg/day(*n* = 187), ansofaxine 160 mg/day(*n* = 186), or placebo(*n* = 185). The primary efficacy endpoint was the Montgomery-Åsberg Depression Rating Scale (MADRS) total score change from baseline to the end of the study. Safety indexes included adverse events, vital signs, physical examination, laboratory tests, 12-lead electrocardiogram (ECG), and evaluation of suicide tendency and sexual function. Significant differences were found in mean changes in MADRS total score at week 8 in the two ansofaxine groups (80 mg, −20.0; 160 mg, −19.9) vs. placebo (−14.6; *p* < 0.0001). All doses of ansofaxine were generally well-tolerated. Treatment-emergent adverse events (TEAEs) were reported by 137 (74.46%) patients in ansofaxine 80 mg group, 144 (78.26%) patients in ansofaxine 160 mg and 125 (67.93%) patients in the placebo group. The incidence of treatment-related adverse events (TRAEs) was 59.2% (109 patients), 65.22% (120 patients) in the 80, 160 mg ansofaxine groups, and 45.11% (83 patients) in the placebo group. The initial results of this trial indicate that ansofaxine at both the 80 mg/day and 160 mg/day was effective and safe in adult patients with MDD. ClinicalTrials.gov Identifier: NCT04853407.

## Introduction

Major depressive disorder (MDD) is one of the most common depressive disorders, with an estimated 12-month prevalence rate of 6.6% and a lifetime prevalence rate of 16.2% [1]. It is becoming a major challenge for public health and medical practice, accounting for 2.5% of the disease burden worldwide [2]. Furthermore, the coronavirus disease 2019 (COVID-19) pandemic seems to aggravate the burden of depression disorder since studies indicate that mental health symptoms, including depression, anxiety, insomnia, and acute stress are highly prevalent during the COVID-19 pandemic, especially in infected individuals, people with suspected infection, and people having close contact with COVID-19 patients [3, 4].

The pathogenesis of MDD is complex, including structure and function alterations of discrete brain regions, especially the hippocampus [5–7], prefrontal and limbic areas [8], parietal-temporal regions, and temporal regions [9], which are functionally regulated by monoaminergic neurotransmissions, such as serotonin (5-HT), norepinephrine (NE) and dopamine (DA), in an interdependent and interconnected manner [10, 11]. Disturbances in the 5-HT, NE, and DA signaling pathways, resulting in the onset and progression of MDD [10, 12, 13].

Currently, most approved antidepressants for MDD primarily focus on the monoamine neurotransmitters 5-HT and NE. However, there are certain limitations to these antidepressants. For first-generation antidepressants, such as tricyclic antidepressants (TCAs) and monoamine oxidase inhibitors (MAIOs), most of them have a significant adverse effect profile as well as lethality in overdose and are not commonly used. The second generation of antidepressants, such as selective serotonin reuptake inhibitors (SSRIs) and serotonin-norepinephrine reuptake inhibitors (SNRIs), are thought to carry less significant side effects and recommended as first-line treatment for most patients with MDD. The response rate to these drugs is moderate (40–60%), and the remission rate is relatively low [14, 15]. Even though the treatment is effective, it needs to take weeks to get a therapeutic effect. This is a particular concern when some depressed patients are at imminent risk of suicide. Meanwhile increasing evidence suggests that second-generation antidepressants, such as SSRIs, are ineffective in treating positive affect deficits, such as motivation and reward-related cognitive impairment in depression [16, 17]. Based on the current treatment dilemmas and challenges, we could conclude that focusing on monoamine neurotransmitters of 5-HT and NE is far from sufficient.

Anhedonia, loss of motivation, energy, and attention, which is one of the core symptoms of MDD, have been linked to the dysfunction of the DA system. DA is produced in small nuclei of tightly clustered neurons-predominantly the ventral tegmental area (VTA) and substantia nigra pars compacta (SNc). In animal models of depression, the DA system is downregulated due to hyperactivity of the infralimbic subregion (ilPFC), driving activity in the inhibitory basolateral amygdala (BLA)-ventral pallidum (VP) pathway while attenuating excitation via the Re-ventral subiculum of the hippocampus (vSub)-nucleus accumbens (NAc)-VP pathway [18, 19]. It has also been reported that increasing midbrain-cortical-marginal pathway synaptic cleft, hypothalamus DA levels can improve treatment lag in depression, anhedonia symptoms and sexual dysfunction [19–21]. Considering the critical role of DA in the pathophysiology and treatments of depression, a combination of dopamine reuptake inhibition (bupropion) and either an SSRI or an SNRI clinical trial have been carried out. The results of the trial demonstrate that this combination is generally well tolerated, can boost antidepressant response, and reduce SSRIs or SNRIs-associated sexual side effects [22]. This evidence has led to the development of triple reuptake inhibitors (TRIs) drugs that simultaneously inhibit the reuptake of all three monoamines(5-HT, NE, and DA).

Ansofaxine hydrochlorideis [(±)-4-(2-(dimethylamino)-1-(1-hydroxycyclohexyl) ethyl) phenyl 4-methylbenzoate hydrochloride], a new triple reuptake inhibitor chemical entity, is formulated as an extended-release (ER) oral tablet for the treatment of adults with MDD. Ansofaxine inhibits the function of the transport proteins responsible for clearing dopamine, serotonin, and norepinephrine from the synaptic cleft. A Microdialysis study shows that ansofaxine can increase all three neurotransmitters in the striatum after oral drug administration. Furthermore, in the vitro binding affinities and reuptake inhibition effects trail, ansofaxine exhibits high affinities at SERT, NET, and DAT and differential inhibition potencies at the three transporters. The concentration required to inhibit uptake (IC50) values of ansofaxine on radioligand binding to SERT, NET, and DAT were 1,330 ± 82.5 nM, 2,200 ± 278 nM, and 227 ± 21.7 nM, respectively. The IC50 values for 5-HT, NE, and DA reuptake were 31.4 ± 0.4 nM, 586.7 ± 84 nM, and 733.2 ± 10 nM, respectively. The preclinical safety studies showed that ansofaxine was negative in the genotoxicity combination studies and had acceptable toxicity profiles in the general toxicity studies, fertility and early embryonic development studies, and good safety and tolerance [23–26]. For clinical trials, the preliminary efficacy of ansofaxine for treating MDD has been shown in a randomized, double-blind, placebo-controlled phase 2 study comparing flexible doses of ansofaxine to placebo. The safety and good tolerance of ansofaxine have been demonstrated in phase 1 and 2 clinical trials [27]. This study aims to verify the efficacy and safety of ansofaxine in a larger sample of MDD patients.

## Materials and methods

### Study design

The study was a phase 3, multicenter, double-blind, randomized, fixed-dose, placebo-controlled clinical trial. The trial was conducted at 22 centers in China from December 2018 to December 2020. It was performed in accordance with the principles of Good Clinical Practice and the Declaration of Helsinki. All patients provided written informed consent prior to enrollment. Ethical approval was obtained from the local research ethics committees. See the full protocol in the supplementary information protocol.

### Participants

Participants were eligible if they were male and female outpatients aged 18 to 65 years with a diagnosis of MDD, meeting the DSM-5 (*Diagnostic and Statistical Manual of Mental Disorders*, 5th Edition) criteria for depressive disorder (296.2/296.3) with either single or recurrent episodes, without psychotic features. We used International Neuropsychology Interview (MINI) for psychiatric evaluation and diagnosis. Montgomery-Åsberg Depression Rating Scale (MADRS) total score [28] ≥26 points and Clinical Global Impressions-Severity (CGI-S) score [29] ≥4 points at screening visits were eligible for inclusion in the study. All sexually active patients in the study were required to use medically acceptable contraception.

Exclusion criteria for all participants were: any other psychotic disorders (except for MDD); depressive disorder secondary to other mental illnesses or physical illnesses as well; be allergic to venlafaxine and desvenlafaxine; failed to respond to a full course of treatment with venlafaxine; a clear suicide attempt or behavior; pregnant or lactating women, or patient with a planned pregnancy in the near future; a history of seizures (except for seizures caused by febrile convulsions in children); received electroconvulsive therapy (ECT) or systematic psychotherapy (interpersonal relationship therapy, dynamic therapy, cognitive behavioral therapy) or transcranial magnetic stimulation (TMS) within 3 months prior to screening; receiving light therapy 2 weeks prior to screening; stopping psychotropic drugs for less than 7 half-lives prior to study randomization (monoamine oxidase inhibitor for at least 2 weeks, fluoxetine for at least 1 month); a history of gastrointestinal disease known to interfere with drug absorption or excretion; clinical abnormalities on total bilirubin (TBIL), alanine aminotransferase (ALT) or aspartate aminotransferase (AST), creatinine, thyroid stimulating hormone (TSH), or 12-lead electrocardiogram (ECG) at screening period; participating in other clinical trials within 3 months prior to screening; serious acute or chronic diseases, mental illnesses, of which investigators believe that the subjects are not suitable for this study.

### Procedures

The study comprised a 1-week screening period and a 8-week double-blind treatment period (DBTP). During the screening period, the patients underwent physical examination, depression scale evaluations, 12-lead ECG, and laboratory examinations.

Eligible patients were randomized at a 1:1:1 ratio to ansofaxine 80 mg/da y, 160 mg/day, or placebo group using a minimization random allocation system. The system used baseline MARDS total score, age, and gender as prognostic factors to ensure equilibrium among groups and monitored enrollment and allocated medication using a code matching the assigned medication.

Study medication was dispensed ansofaxine 40 and 80 mg tablets and matching placebo tablets, identical in appearance and packaging. Participants would take one tablet of ansofaxine (40 mg) or one matched placebo pill daily in the first week and increase to one tablet of ansofaxine (80 mg) daily (or a matching placebo pill) daily in the second week. From week three to the end of DBTP, participants in the low-dose group administrated 80 mg tablet of ansofaxine and one 80 mg matched placebo pill, the high-dose group administrated two 80 mg tablets of ansofaxine, and the placebo group administrated two 80 mg matched placebo pills(see the electronic supplementary protocol.

For every visit, the patients would go through vital signs monitoring, depression scale evaluations, AEs record, and combined medication record. The 12-lead ECG and laboratory examinations would be retaken at the end of week 4 and 8.

### Outcomes

The primary efficacy endpoint was the change of MADRS total score from baseline to the end of week 8. Secondary efficacy endpoints were the changes from baseline of the following scores at the end of week 8: Hamilton Rating Scale for Depression-17 item (HAM-D_17_) [30] total score; CGI-S score; Hamilton Anxiety Rating Scale (HAMA) [31] total score; Sheehan Disability Scale (SDS) total score [32]. They also included the following at the end of week 8: CGI-I score; response rate in MADRS (defined as a ≥ 50% decrease in MADRS total score from baseline); response rate in HAM-D_17_ (defined as a ≥ 50% decrease in HAM-D_17_ total score from baseline); remission rate in MADRS (defined as a MADRS total score ≤12); remission rate in HAM-D_17_ (defined as a HAM-D_17_ total score ≤ 7).

Safety assessments included adverse events (AEs), withdrawal due to AEs, vital signs, physical examination, laboratory tests (hematology, serum chemistry, serum prolactin, and urinalysis), and 12-lead ECGs. Suicide ideation and behavior and sexual function were assessed using Colombia-Suicide Severity Rating Scale (C-SSRS) [33] and Arizona Sexual Experience Scale (ASEX) [34], respectively.

### Sample size

Sample size estimates were based on the primary efficacy endpoint, MADRS total score changes from baseline, referred to phase II clinical trial result and calculated by PASS 15 software (NCSS, LLC, USA). 140 patients were calculated in each group. Considering a 25% drop-out rate, 186 subjects were planned in each group, and a total of 558 subjects were in three groups.

### Blinding

Patients, clinicians, and independent outcome raters were masked to treatment allocation, and tablets in each group were identical in package and appearance. The database was locked when the final visit of the last randomized patient was completed, data entry for all patients was completed, and the database for all patients was deemed clean without any outstanding queries. After all of the above work was completed, an independent statistician operated the unblinding method, and a two-level unblinding method was used in the study.

### Statistical analysis

Statistical analysis was performed using SAS®9.4 (SAS Institute, Cary, NC, USA). Continuous data were summarized in terms of the mean and standard deviation (SD). Categorical variables were summarized in terms of frequency and percentages. The efficacy analysis was based on the full analysis set (FAS), and the safety analysis was based on the safety analysis set (SS), see in the supplementary information protocol.

The primary efficacy analysis compared MADRS at Week 8 in the FAS using a mixed model for repeated measurements (MMRM) with the change from baseline in the MADRS total score at each time point after treatment as a dependent variable, baseline MADRS scale total scores as a covariate, baseline MADRS total score strata (26–34, ≥35), age strata (18–30, 31–40, 41–50, and 51–65 years), gender, and the group as fixed effects, and each fixed effect factor and the covariate were nested within each visit. The adjusted mean and its 95% confidence interval (CI) of the changes from baseline in MADRS scale total scores to week 8 in each group were calculated according to this model, as well as the difference and its 95% CI of the adjusted mean for the investigational drug 80 mg group, 160 mg group versus the placebo group.

For safety analysis, the type, severity, frequency, and relationship with the study drug for all treat-emergent adverse events (TEAEs) were summarized, and subjects who dropped out from the study due to adverse events and those with serious adverse events were listed. AEs were coded using the Medical Dictionary for Regulatory Activities (MedDRA). Shift tables were used to summarize the changes of clinical significance evaluation (based on the clinician’s evaluation) of laboratory parameters, and all abnormal parameters with clinical significance were listed.

## Results

### Participant flow and sample characteristics

**Fig. 1:**
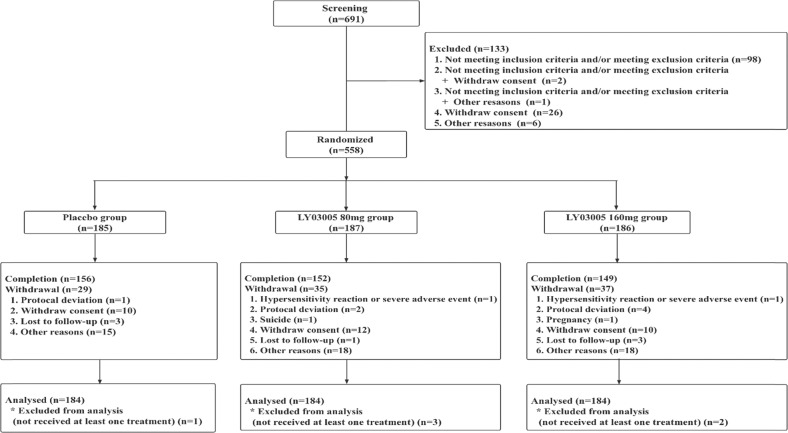
Study design and flow diagram. AE, adverse event.

Of the 691 subjects who provided informed consent, one hundred and thirty-three were screened as failure and 558 patients were finally enrolled in the DBTP (Fig. 1). 185, 187, and 186 patients were randomly assigned to the placebo, ansofaxine 80 mg/day and 160 mg/day groups, respectively. During the DBTP, a total of 101 (18.10%) patients discontinued from the study, including 29 (15.68%) patients in the placebo group, 35 (18.72%) in the ansofaxine 80 mg/day group and 37 (19.89%) in the ansofaxine 160 mg/day group, presenting a comparable withdrawal rate among three groups (*p* > 0.05). Among 558 patients, 552 were included in the FAS and evaluated for efficacy. There were no significant differences in demographic and clinical characteristics at baseline between groups. A total of 457 patients completed the DBTP (Table 1). All subjects who completed the 8-week DBTP without significant protocol deviation advanced to the per-protocol set (PPS, *n* = 445, 79.75%).

**Table 1:** Demographic and baseline characteristics (FAS).

Characteristic	Placebo (*n* = 184)	LY03005 80 mg (*n* = 184)	LY03005 160 mg (*n* = 184)
Age (year)			
Mean±SD	29.7 ± 10.93	29.4 ± 10.49	29.5 ± 10.66
Sex, *n* (%)			
Male	49 (26.63)	52 (28.26)	50 (27.17)
Female	135 (73.37)	132 (71.74)	134 (72.83)
Han, *n* (%)	165 (89.67)	173 (94.02)	168 (91.30)
Weight (kg)			
Mean±SD	59.54 ± 11.49	59.87 ± 11.02	57.93 ± 11.56
Numbers of MDE			
Mean±SD	1.7 (1.12)	1.7 (1.67)	1.5 (1.24)
MADRS total score			
Mean±SD	31.3 ± 4.22	31.6 ± 3.94	31.2 ± 3.59
HAM-D_17_ total score			
Mean±SD	21.7 ± 4.20	22.4 ± 4.31	21.9 ± 4.43
CGI-S total score			
Mean±SD	4.8 ± 0.67	4.9 ± 0.63	4.9 ± 0.63
HAMA total score			
Mean±SD	19.0 ± 5.90	19.8 ± 6.19	18.9 ± 5.49
SDS total score			
Mean±SD	16.9 ± 6.25	16.2 ± 5.56	16.0 ± 5.28
ASEX total score			
Mean±SD	20.8 ± 5.02	21.2 ± 4.97	20.7 ± 4.87

*ASEX* Arizona Sexual Experiences Scale, *CGI-S* Clinical Global Impression–Severity, *FAS* full-analysis set, *HAMA* Hamilton Rating Scale for Anxiety, *HAM-D17* 17-item Hamilton Rating Scale for Depression, *MADRS* Montgomery–Asberg Depression Rating Scale, *MDE* major depressive episode, *SDS* Sheehan Disability Scale.

### Primary efficacy endpoint

**Fig. 2:**
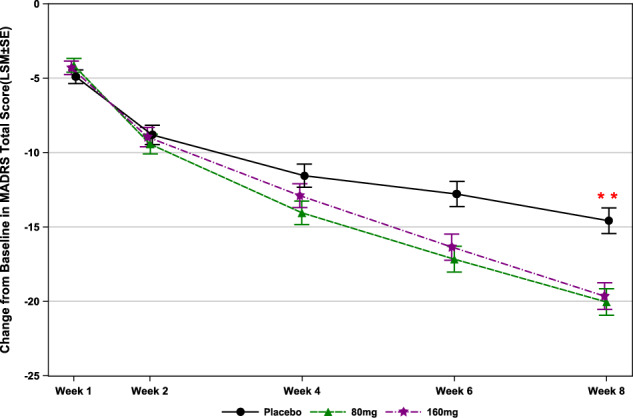
The Changes from baseline in MADRS total score (FAS). ** indicates *P* < 0.0001. FAS, full analysis set. MADRS, Montgomery- Åsberg Depression Scale.

After 8-week treatment, both dosages of ansofaxine were statistically significantly superior to placebo in the adjusted mean changes from baseline in the MADRS total score with the least squares mean (LSM) difference to placebo of −5.46 [ansofaxine 80 mg/day, 95% CI: (−7.14, −3.77), *p* < 0.0001] and −5.06 [ansofaxine 160 mg/day, 95% CI: (−6.75, −3.37), *p* < 0.0001] (Table 2, Fig. 2).

**Table 2:** Efficacy analyses, changes from baseline to week 8 (FAS, MMRM).

	Adjusted change from baseline [Mean (SD)]	Difference in adjusted means (95% CI) (LSM)	*P* value versus placebo
*Primary efficacy variable*			
*MADRS total score*			
Placebo	−14.6 (9.15)		
LY03005 80 mg	−20.0 (7.64)	−5.46 (−7.14, −3.77)	<0.0001
LY03005 160 mg	−19.9 (7.29)	−5.06 (−6.75, −3.37)	<0.0001
*Secondary efficacy variable*			
*HAM-D*_*17*_ *total score*			
Placebo	−9.4 (7.19)		
LY03005 80 mg	−13.4 (6.12)	−3.57 (−4.87, −2.27)	<0.0001
LY03005 160 mg	−13.1 (5.83)	−3.24 (−4.54, −1.94)	<0.0001
*HAMA total score*			
Placebo	−8.1 (6.39)		
LY03005 80 mg	−11.5 (5.84)	−3.09 (−4.24, −1.95)	<0.0001
LY03005 160 mg	−11.1 (5.60)	−2.76 (−3.91, −1.61)	<0.0001
*SDS total score*			
Placebo	−6.9 (6.05)		
LY03005 80 mg	−8.3 (6.59)	−1.68 (−2.90, −0.45)	0.0077
LY03005 160 mg	−8.2 (6.11)	−1.59 (−2.82, −0.35)	0.0120
*CGI-S score*			
Placebo	−1.8 (1.26)		
LY03005 80 mg	−2.4 (1.09)	−0.66 (−0.89, −0.43)	<0.0001
LY03005 160 mg	−2.5 (1.02)	−0.69 (−0.92, −0.46)	<0.0001
*MADRS anhedonia factor score*			
Placebo	−5.08 (0.32)		
LY03005 80 mg	−6.66 (0.33)	−1.58 (−2.24, −0.92)	<0.0001
LY03005 160 mg	−6.68 (0.34)	−1.60 (−2.26, −0.94)	<0.0001

*MADRS* Montgomery–Asberg Depression Rating Scale, *HAM-D17* 17-item Hamilton Rating Scale for Depression, *HAMA* Hamilton Rating Scale for Anxiety, *SDS* Sheehan Disability Scale, *CGI-S* Clinical Global Impression–Severity.

### Secondary efficacy endpoints

The adjusted mean changes from baseline in the HAM-D_17_ total score were significantly higher in subjects administered ansofaxine 80 and 160 mg than in those administered placeboes after 8 weeks of treatment. The LSM difference to placebo was −3.57 [95% CI: (−4.87, −2.27), *p* < 0.0001] for ansofaxine 80 mg/day group and −3.24 [95% CI: (−4.54, −1.94), *p* < 0.0001] for ansofaxine 160 mg/day group at the end of week 8 (Table 2,Supplementary Fig. 1).

A statistical significance was observed in the mean changes of CGI-S score, HAMA total score, and SDS total score from baseline for both dosages of ansofaxine *vs*. placebo (*p* < 0.05, Table 2).

**Fig. 3:**
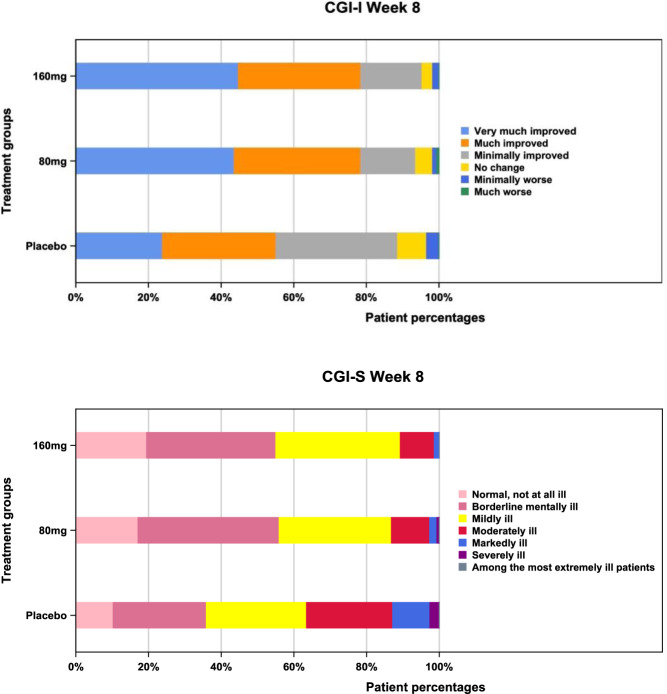
Distribution of CGI-I score and CGI-S score at the last observation (FAS). FAS, full analysis set; CGI-I, Clinical Global Impressions-Improvement. CGI-S, Clinical Global Impressions-Severity.

CGI-I and CGI-S scores differed significantly for ansofaxine in both dose groups *vs*. placebo (*p* < 0.0001). The percentage of patients with a CGI-I score of 1 (very much improved) or 2 (much improved) was both around 78.5% in the ansofaxine 80 mg/day and 160 mg/day groups, respectively, whereas it was only 55.1% in the placebo group (Fig. 3). The percentage of patients with CGI-S score of 1 (normal, not at all ill) or 2 (borderline mentally ill) was around 55.03% and 55.93% for 160 mg and 80 mg group, respectively, whereas the placebo group was only 35.9%.

MADRS response rate at the end of week 8 was achieved in 79.89%, 73.91%, and 42.39% in the ansofaxine 80 mg, 160 mg, and placebo groups, respectively. The difference reached a statistical significance for ansofaxine 80 mg/day group *vs*. placebo [odds ratio (OR) and 95% CI: 5.68 (3.54, 9.12) as well as ansofaxine 160 mg/day group *vs*. placebo group [OR and 95% CI: 4.02 (2.57, 6.29)]. MADRS remission rate at the end of week 8 was 51.63%, 52.17%, and 30.98% in the ansofaxine 80 mg/day, 160 mg/day, and placebo groups, respectively, and a statistically significant difference was obtained for ansofaxine 80 mg/day group *vs*. placebo [OR and 95% CI: 2.45 (1.59, 3.79)] as well as 160 mg/day group vs. placebo group [OR and 95% CI: 2.49 (1.62, 3.84)]. See Supplementary Fig. 2 for details.

Patients in the ansofaxine 80 and 160 mg groups had a significantly higher HAM-D_17_ response rate at the end of week 8 than those in the placebo group respectively (Supplementary Fig. S3), resulting in OR of 4.29 [95% CI: (2.74–6.73)] for the 80 mg/day group and 2.87 [95% CI: (1.86–4.41)] for the 160 mg/day group. The remission rate in HAM-D_17_ of both dosages of ansofaxine was statistically significantly superior to placebo [OR and 95% CI for ansofaxine 80 mg/day group *vs*. placebo group: 2.38 (1.50, 3.78); for ansofaxine 160 mg/day group *vs*. placebo group: 2.03 (1.28, 3.21) (Supplementary Fig. 3).

At the end of the final evaluation, the changes of SDS total score from baseline were −8.3 ± 6.59, −8.2 ± 6.11, and −6.9 ± 6.05 in 80 mg, 160 mg and placebo groups, respectively (Table 2). The between-group difference *vs*. placebo was statistically significant in LSM with −1.68 [95% CI: (−2.90, −0.45), *P* = 0.0077] for 80 mg group, and −1.59 [95% CI: (−2.82, −0.35); *P* = 0.0120] for 160 mg group.

To understand how the MADRS anhedonia factor was influenced in this study, we did an additional analysis. Mean changes in MADRS anhedonia factor score from baseline to the end of week 8 were −6.66,−8, and −5.08 in the ansofaxine 80 mg, 160 mg, and placebo groups, respectively. A statistically significant decrease in LSM compared to placebo was observed in the ansofaxine 80 mg/day with a value of −1.58 [95% CI: (−2.24, −0.92), *p* < 0.0001] and in the ansofaxine 160 mg/day group with −1.60 [95% CI: (−2.26, −0.94), *p* < 0.0001] (Table 2).

### Safety

TEAEs were reported by 137 (74.46%) patients in the ansofaxine 80 mg group, 144 (78.26%) patients in the ansofaxine 160 mg, and 125 (67.93%) patients in the placebo group. Most TEAEs were mild or moderate in severity. Fourteen subjects had severe TEAEs, reported by 4 (2.17%, 5 events), 4 (2.17%, 6 events), and 6 (3.26%, 8 events) in the ansofaxine 80 mg, ansofaxine 160 mg, and placebo groups, respectively. The severe TEAEs in 80 mg group were abdominal pain, diarrhoea, headache, insomnia, and suicidal ideation. The severe TEAEs in 160 mg group were nausea, vomiting, constipation, white blood cells, urine positive, and urine ketone body present. The severe TEAEs in the placebo group were abdominal pain, white blood cells urine positive, blood creatine phosphokinase increased, dizziness, paresthesia, palpitations, dysphoria, and spinal osteoarthritis.

A total of 31 subjects withdrew from the trial due to TEAEs; 25 out of the 31 subjects withdrew from the trial due to the study drug as judged by the investigator, including seven patients (3.80%) in the 80 mg ansofaxine group, thirteen patients (7.07%) in the 160 mg ansofaxine group, five patients (2.72%) in the placebo group. Headache (4 cases) and insomnia (3 cases) were the top two TRAEs that resulted in withdrawal in the 80 mg group, whereas nausea (6 cases) and abdominal discomfort (4 cases) were the top two causes of withdrawal in the 160 mg group. Unlike the ansofaxine dose groups, the top two reasons causing withdrawal in the placebo group were dizziness (2 cases) and palpitations (2 cases).

The incidence of treatment-related adverse events (TRAEs) was reported to be 59.24% (109 cases, 233 events), 65.22% (120 cases, 296 events), and 45.11% (83 cases, 170 events) in ansofaxine 80 mg, 160 mg, and placebo groups, respectively.TRAEs with an incidence ≥5% in any group, sorted by descending incidence in each System Organ Class (SOC), were shown in Table 3. The most common three TRAEs in ansofaxine groups were nausea, dizziness, and dry mouth, of which the incidence of nausea and dry mouth was higher in the 160 mg group *vs*. 80 mg group, whereas the incidence of dizziness was lower in the 160 mg group *vs*. 80 mg group.

**Table 3:** Treatment-related adverse events with an incidence ≥ 5% in any treatment group during double-blind period (SS).

SOC (PT)	Placebo *n* (%)	LY03005 80 mg *n* (%)	LY03005 160 mg *n* (%)	
*Gastrointestinal disorders*	42 (22.83)	60 (32.61)		73 (39.67)
nausea	20 (10.87)	39 (21.2)		48 (26.09)
dry mouth	13 (7.07)	16 (8.70)		19 (10.33)
abdominal discomfort	2 (1.09)	5 (2.72)		11 (5.98)
vomiting	1 (0.54)	2 (1.09)		10 (5.43)
*Nervous system disorders*	22 (11.96)	44 (23.91)		38 (20.65)
dizziness	13 (7.07)	24 (13.04)		19 (10.33)
lethargy	2 (1.09)	8 (4.35)		12 (6.52)
*Investigations*	24 (13.04)	26 (14.13)		34 (18.48)
*Cardiac disorders*	9 (4.89)	12 (6.52)		14 (7.61)
palpitations	6 (3.26)	9 (4.89)		11 (5.98)
*Metabolism and nutrition disorders*	1 (0.54)	10 (5.43)		12 (6.52)
decreased appetite	1 (0.54)	10 (5.43)		8 (4.35)
*Systemic disease and various reactions of administration site*	6 (3.26)	8 (4.35)		10 (5.43)
*Psychiatric disorders*	7 (3.80)	11 (5.98)		8 (4.35)

Note: There were 184 patients in each group. *SOC* system organ class, *PT* preferred term.

A total of 6 cases of serious adverse events (SAE) occurred in 5 subjects throughout the study, including 2 cases in 2 subjects in the 80 mg group (wound infection, arteriosclerosis coronary artery), 3 cases in 2 subjects in the 160 mg group (ectopic pregnancy and abortion induced in 1 subject, depression in the other subject) and 1 case in 1 subject in the placebo group (spinal osteoarthritis), and all of the cases were judged by investigators as unrelated with the study drug. No deaths occurred in this study. The detailed information regarding vital signs, physical examination, laboratory tests, 12-lead ECG, and C-SSRS was described in the supplementary information. After the 8-week treatment, changes from baseline in ASEX total score were (−1.6 ± 4.83), (−1.1 ± 4.70) in ansofaxine 80 mg and 160 mg groups, respectively, and no significant difference was shown vs. placebo (−0.5 ± 4.27). No cases were reporting newly developed sexual dysfunction in this study.

## Discussion

On the primary outcome measure of changes from baseline in MADRS total score, the superiority of both dosages of ansofaxine to placebo was statistical significance, with a prominent mean treatment difference of −5.46 points for 80 mg/day and −5.06 points for 160 mg/day. However, the change in the total MADRS score from baseline in the placebo group was 14.6 points, which was slightly higher than usual. The reduction compared with placebo is much greater than the two-point average for approved antidepressants [35, 36], and the 3 points recognized by National Institute for Health and Clinical Excellence (NICE) as clinically significant is extremely remarkable in randomized controlled trials of antidepressants.

Response rate is also frequently used as a measure of clinical relevance, and a average of approximately 15% difference between drug and placebo is regarded as sufficient to establish antidepressant treatment advantage [37, 38]. The response rate, as measured by ≥50% reduction in MADRS total score, was significantly higher for ansofaxine 80 mg/day (79.89%) and 160 mg/day (73.91%) versus placebo (42.39%), respectively. Compared with placebo, differences in HAM-D_17_ response rate vs. placebo (33.69% in ansofaxine 80 mg/day and 25.31% in ansofaxine 160 mg/day). Both differed from the placebo exceeded the 15% threshold for clinical relevance.

Similarly, treatment with ansofaxine was associated with a statistically significant greater MADRS remission rate (MADRS total score ≤10) of 51.63% and 52.17% in the 80 mg and 160 mg groups, respectively (*vs*. 30.98% for placebo). The changes of HAM-D_17_ remission rate vs. placebo (16.31% with 80 mg/day and 13.59% with 160 mg/day) were two-time more than 10%, which was suggested as an absolute inter-group difference to determine clinical significance [39].

The results are much higher than the moderate response rate of 40%–60% and low remission rate demonstrated by many first-line or widely prescribed antidepressants [14, 15]. For instance, the response rate of MADRS was reported to be 48%-65% in venlafaxine [40, 41]. The response rate and remission rate of MADRS (MADRS total score ≤10) were 46.3% ~51.6% and 28.7% ~32.3% in the vortioxetine 5–20 mg/day dose range [36], respectively. Clinically, vilazodone showed a 49% and 33.7% response rate and remission rate of MADRS, respectively [42].

Significant differences versus placebo were consistently observed across secondary and additional efficacy measures in treatment groups. Improvement in HAM-D_17_ total score, CGI-S score, SDS total score, response, and remission rate in MADRS, HAM-D_17_ was noted in all ansofaxine groups versus placebo at week 8; the difference was statistically significant versus placebo at the 80 mg and 160 mg doses. Research suggests that both these scales are sensitive to treatment effects and that they measure independent symptom and functional domains [28, 30]. These results suggested that ansofaxine is a promising antidepressant with significant efficacy.

Ansofaxine showed a beneficial effect on symptoms of anxiety in patients with MDD over placebo, as demonstrated by a decrease of HAMA total score of 11.5 (80 mg/day) and 11.1 (160 mg/day) throughout the 8-week treatment period (*vs*. 8.1 in placebo), which were comparable with the changes in patients treated with vortioxetine of 9.6 (15 mg/day) and 11.1 (20 mg/day) [43]. This result verified the anxiolytic efficacy of ansofaxine in these patients, offering a therapy option for MDD patients with anxiety symptoms.

A predicament for treatment in patients with MDD is the common symptom and critical diagnostic criterion of anhedonia, a predictor of non-response to plenty of antidepressants [44, 45]. Nevertheless, a good result was revealed in this trial with both dosages of ansofaxine showed the superiority on reduction of MADRS anhedonia factor score(−6.66 in ansofaxine 80 mg/day and −6.68 in ansofaxine 160 mg/day vs. −5.08 in placebo; *p* < 0.0001), offering a prospective pharmacological approach for the treatment of anhedonia in patients with MDD, especially, there is no specific therapy recommended for anhedonia so far. The analysis of the anhedonia factor score shows that ansofaxine is efficacious in improving a core domain of affective deficit that is presumed to be related to hypodopaminergia [46, 47]. These results support the hypothesis that ansofaxine, as a potential TRI, is more valuable than mono- or dual-acting antidepressants in treating the broader symptom domains of depression, especially SSRIs of questionable efficacy in patients with anhedonia.

It was observed that changes from baseline to the end of week 8 in SDS score were significantly greater for ansofaxine, with the inter-group difference *vs*. placebo were −1.68 (80 mg/day; *p* = 0.0077) and −1.59 (160 mg/day; *p* = 0.012). The results indicated that ansofaxine might favor the quality of life of patients with MDD. The improvement could be correlated with the relief of depressive symptoms, especially the mitigation of anhedonia.

Treatment with ansofaxine was generally safe and well tolerated in this trial, and TEAEs were mild to moderate in severity in most cases. No new safety findings were observed, and no new, unexpected, drug-related, serious AEs occurred with ansofaxine therapy. The incidence of TEAEs was similar to the data in a meta-analysis [48], with the incidence of TEAE being 73.3%–73.6% for escitalopram, 78.2% for other SSRIs (citalopram, fluoxetine, paroxetine, and sertraline), and 77.4% for SNRIs (venlafaxine and duloxetine). The discontinuation rate due to TRAEs was 3.80%, 7.07%, and 2.72% in ansofaxine 80 mg/day, 160 mg/day, and placebo groups, respectively. Whereas the discontinuation rate due to TRAEs was 12% in venlafaxine (37.5–225 mg/day) [49], 4.1% in 50 mg/day desvenlafaxine, and 8.5% in 100 mg/day desvenlafaxine [50], and 5%, 6%, 8% and 8% in vortioxetine 5 mg/day, 10 mg/day, 15 mg/day and 20 mg/day [51], respectively, suggesting that ansofaxine has a similar or even better profile of safety and tolerability compared with other common antidepressants. The most common ADRs (incidence ≥5%) were nausea, dry mouth, abdominal discomfort, vomiting, dizziness, lethargy palpitations, and decreased appetite, similar in patterns to those reported by SSRIs and SNRIs [48, 52].

Ansofaxine 80 and 160 mg did not increase suicide risks compared to the placebo group. There was no significant difference in laboratory tests and weight in the ansofaxine 80 mg/day and 160 mg/day group compared with the placebo. No clinically relevant changes were observed in 12-lead ECG and vital signs parameters.

Sexual dysfunction, a frequent side effect of drugs with serotonin reuptake inhibitor properties, is a common reason for therapeutic discontinuation. It had been reported that the prevalence of sexual dysfunctions in patients on antidepressants was two times higher than that in patients on control (50% *vs*. 24%) [48]. A meta-analysis reported that the incidence of treatment-emergent sexual dysfunction (TESD) in antidepressant treatment ranged from 26% to 80% [53]. In this study, the sexual dysfunction was assessed with ASEX and spontaneous report, which did not show a significant difference in all ansofaxine groups versus placebo at the endpoint. These results indicated that ansofaxine has a low risk of TESD.

Psychiatric disorders, such as hypomanic/manic, psychosis, is an adverse effects of the drug in people receiving antidepressant therapy. However, AEs related to psychiatric disorders were mild or moderate,while hypomanic/manic or psychosis cases were not collect in our clinical trial. The incidence of hypomanic/manic or psychosis depends on the study sample characteristics (inpatient vs. outpatient populations), antidepressant class (studies with TCAs or MAOIs monotherapy reported higher rates), diagnostic criteria (DSM vs. Research Diagnostic Criteria), and study duration (studies with longer follow-up reported higher rates) [54, 55]. All the elaborated rationales, including shorter time with smaller sample size, recruited outpatients with primary MDD and ansofaxine not categorized as TCAs, may not be sufficient to capture a series of AEs.

In conclusion, the results of this trial demonstrate that both dosages of ansofaxine 80 mg and 160 mg are safe, generally well tolerated, and remarkably effective at a clinically relevant level for the treatment of MDD.

### Limitations

Limitations of our study include the need for more active controls, the size of the sample, the short duration of treatment, and strict inclusion and exclusion criteria that may limit the generalizability of the results. Additional demographic factors (e.g., the age of participants and the proportion of the first episode) may have further affected the results of our study. Therefore, more evidence could be required to prove the efficacy of ansofaxine in older MDD patients.

## Supplementary information

Protocol

Supplementary Information
