# Supplementary material for: A phase 3, multicenter, double-blind, randomized, placebo-controlled clinical trial to verify the efficacy and safety of ansofaxine (LY03005) for major depressive disorder
Source: Transl Psychiatry. 2023 May 10;13:163. doi: 10.1038/s41398-023-02435-0 (PMC10171157; doi:10.1038/s41398-023-02435-0)
Supplement: Supplementary file 1 — Protocol [file 41398_2023_2435_MOESM1_ESM.pdf]

Clinical Study Protocol

**Protocol Title:**	A Phase III, A multicenter, double-blind, randomized, placebo-controlled study Verify the efficacy and Safety of Ansofaxine hydrochloride extended-release tablets for major depressive disorder.
**Approval number:**	2015L01158/2015L01159/2015L01160/2015L01161
**Protocol Number:**	LY03005/CT-CHN-305
**Clinical center**	Peking University Sixth Hospital
**Principal Investigator**	Zhang, Hongyan
**Sponsor:**	Shandong Luye Pharmaceutical Co., Ltd
**Version Number:**	1.0
**Version Date:**	09-July-2018

Confidentiality Statement

This document contains confidential information of Shandong Luye Pharmaceutical Co., Ltd. and you agree to keep this information confidential when accepting or reviewing this document. You may not copy this document or disclose it to others (Except for those as required by applicable laws or regulations or with written permission) and it may not be used for other purposes that are not approved.

Contact Information

Sponsor contact information

Shandong Luye Pharmaceutical Co., Ltd

Name: Dr. Guo Shuren, Vice President, Clinical Medical Research Center

Phone: 010-52819345

Mobile: 13501029003

Fax: 010-52819339

E-mail: guoshuren@luye.com

Address: No. 15, Chuangye Road, High-tech Zone, Yantai City, Shandong Province

If you can not reach the contact above, you can contact the one below for all requiremented information related to the study:

Name: Han Baihuan, Senior Manager, Clinical Medical Research Center

Mobile: 13761005330

Fax: 010-52819339

Email: hanbaihuan@luye.com

Sponsor’s designed medical monitor

Name: Zhong Qi, Senior Medical Monitoring Manager, Clinical Medical Research Center

Mobile: 17744502544

Fax: 010-52819339

E-mail: zhongqi@luye.com

Researcher contact

Clinical Research Team: Peking University Sixth Hospital

Name: Zhang Hongyan, Chief Physician/Professor

Office Phone: 010-82013183

Mobile: 13601237138

Fax: 010-82013183

E-mail: sally_zhy@sina.com

Address: No. 51, Huayuan North Road, Haidian District, Beijing

Signature page (Sponsor)

I am hereby signing that I have read this protocol, agree to the trial plan, and will be responsible for sponsoring, applying, organizing, and funding in strict accordance with this protocol, the Good Clinical Practice for Clinical drug Trials (GCP) and relevant national regulations. And monitor this clinical trial.

I will be responsible for appointing an monitor to monitor the operation of this clinical trial.

I will be responsible for establishing a quality management system and appointing quality control or quality assurance personnel to monitor the quality of this clinical trial.

I will be responsible for reporting serious adverse events and unexpected serious adverse reactions (SUSAR) as required by this protocol.

Signature: ______________________________ __________Year ____________

Shandong Luye Pharmaceutical Co., Ltd

Signature page (study leader unit)

I am hereby signing that we have been involved in the development and discussion of this clinical trial protocol, agree to the content of the study protocol, and will strictly follow this protocol, the Good Clinical Practice for Clinical Drug Trials (GCP) and national regulations. I will guide, assist and supervise the authorized personnel of the site in this clinical trial to understand the protocol, study drugs and test procedures, and perform the researcher’s duties. I will only use the informed consent form approved by the Ethics Committee and complete all responsibility for submitting relevant information to this Experimental Ethics Committee. I will accept the inspection of the monitor or auditors dispatched by the sponsoring unit, and accept the inspection by the State Drug Administration (SDA).

main researcher:

Signature: ______________________________ __________Year ____________

Research leader: Peking University Sixth Hospital

Signature page (research participants)

I am hereby signing that we have been involved in the development and discussion of this clinical trial protocol, agree to the content of the trial protocol, and will strictly follow this program, the Quality Control Practice for Drug Clinical Trials (GCP) and national regulations. I will guide, assist and supervise the authorized personnel of the site in this clinical trial to understand the protocol, test drugs and test procedures, and perform the researcher's duties. I will only use the informed consent form approved by the Ethics Committee and complete all responsibility for submitting relevant information to this Experimental Ethics Committee. I will accept the inspection of the monitors or auditors dispatched by the sponsoring unit, and accept the inspection by the State Drug Administration (SDA).

main researcher:

Signature: ______________________________ __________Year ____________

Research site:

Remarks: This page allows copying, page numbers are the same, but different research units should sign this page separately and handwritten research unit name

Signature page (statistical analysis unit for clinical trials)

I am hereby signing that we have been involved in the development and discussion of this clinical trial protocol, agree to the content of the trial protocol, and methods of conduct statistical analysis of clinical trials and follow in strict accordance with this protocol and all applicable laws and regulations.

main in-charge:

Signature: ______________________________ __________Year ____________

Clinical trial statistical analysis unit:

TABLE OF CONTENTS

LIST OF ABBREVIATIONS AND DEFINITION OF TERMS **10**

SUMMARY **12**

1. BACKGROUND INFORMATION **19**

2. OBJECTIVE: **27**

3. STUDY DESIGN REFERENCE **27**

4. STUDY DESIGN **27**

4.1 STUDY METHODS 27
4.2 PLACEBO RATIONAL 28
4.3 DOSE SELECTION RATIONAL 28
4.4 DURIATION OF THE STUDY 28
4.5 SAMPLE SIZE ESTIMATE 28
4.6 RANDOMIZATION METHOD 29
4.7 BLIND METHOD AND DESIGN 29

5. SUBJECT SELECTION **30**

5.1 INCLUSION CRITERIA 30
5.2 EXCLUSION CRITERIA 30
5.3 ELIMINATION CRITERIA 31
5.4 CRITERIA FOR WITHDRAW 32
5.5 RULE OF STOPPING THE TRIAL 33

6. DOSE REGIMEN **33**

6.1 DOSAGE FORMS AND STRENGTHS 33
6.2 STUDY DRUG PACKAGING AND LABELING 33
6.3 DRUG CODING 33
6.4 GROUPING AND ADMINISTRATION METHODS 34
6.5 DOSAGE ADJUSTMENT 34
6.6 DRUG STORAGE 34
6.7 DRUG ACCOUNTABILITY 35
6.8 CONCOMITANT MEDICATIONS 35

7. FOLLOW-UP VISIT **36**

7.1 SCREENING 41
7.2 DOUBLE BLIND TREATMENT STAGE 42

8. EFFICACY AND SAFETY EVALUATION **45**

8.1 EFFICACY ENDPOINT 45
8.2 SAFETY EVALUATION 45

9. SAFETY REPORTING AND PROCEDURES **49**

9.1 ADVERSE EVENT 49
9.2 SERIOUS ADVERSE EVENT 51
9.3 PREGNANCY 53

10. UNBLINDING **54**

11. DATA MANAGEMENT AND STATISTICAL ANALYSIS **55**

11.1 DATA MANAGEMENT 55

12 QUALITY CONTROL AND ASSURANCE 59

13 ETHICS REQUIREMENTS 60

13.1 GUIDANCE OF ETHICAL REQUIREMENTS 60
13.2 INFORMED CONSENT 60
13.3 EARLY TERMINATION TEST 61

14 SUMMARY REPORT 61

15 REVISION OF THE STUDY PLAN 61

16 PAPER PUBLICATION 61

17 DATA SAVING 61

18 REFERENCES 62

ANNEX 1: MONTGOMERY- ÅSBERG DEPRESSION SCALE (MADRS) 63

ANNEX 2: 17 HAMILTON DEPRESSION SCALE (HAM-D_{17}) 65

ANNEX 3: CLINICAL TOTAL IMPRESSION SCALE (CGI) 68

ANNEX 4: HAMILTON ANXIETY SCALE (HAMA) 68

ANNEX 5: SHEEHAN DISABILITY SCALE (SDS) 70

ANNEX 6: COLOMBIA - SUICIDE SEVERITY RATING SCALE (C-SSRS) 72

ANNEX 7: ARIZONA SEXUAL EXPERIENCE SCALE (ASEX) 83

ANNEX 8: DIAGNOSTIC CRITERIA FOR DEPRESSIVE DISORDERS IN THE DIAGNOSTIC AND STATISTICAL MANUAL OF MENTAL DISORDERS, 5TH EDITION (DSM-5) 84

ANNEX 9: CONCISE INTERNATIONAL NEUROPSYCHOLOGY INTERVIEW (MINI) 86

LIST OF ABBREVIATIONS AND DEFINITION OF TERMS

**Abbreviations**	
5-HT	5-hydroxytryptamine
AE	Adverse event
ALT	Alanine aminotransferase
ANCOVA	Covariance analysis
AST	Aspartate aminotransferase
AUC	Blood concentration-time curve area
BP	blood pressure
CGI-I	Clinical Globle Impression Scale - Inprovement
CGI-S	Clinical Globle Impression Scale - Severity of Disease
Cmax or Cmax	Peak concentration
Cr	Creatinine
C-SSRS	Colombia - Suicide Severity Rating Scale
CYP 450	Cytochrome P450 enzyme
DA	Dopamine
DAT	Dopamine transporter
DBP	Diastolic blood pressure
DSM-5	The Diagnostic and Statistical Manual of Mental Disorders, 5th Edition
ECG	Electrocardiogram
eCRF	Electronic case report form
ECT	Electroconvulsive therapy
EDC	Electronic data collection
FAS	Full analysis set
FT3	Free triiodothyronine
FT4	Free thyroxine
GCP	Good Clinical practices
h	hour
HAMA	Hamilton Anxiety Rating Scale
$HAM\text{-}D_{17}$	Hamilton Rating Scale for Depression 17 item
ITT	Intention-To-Treat
LOCF	The last observation is carried forward
MADRS	Montgomery- Åsberg Depression Scale

**Abbreviations**	
MAOIs	Monoamine oxidase inhibitors
MDD	Major Depressive Disorder
MedDRA	International medical term dictionary
MINI	Mini-international neuropsychiatric interview
MMRM	Mixed effect model of repeated measurement data
NaSSA	Noradrenergic and specific serotoninergic antidepressants
NDRIs	Norepinephrine and dopamine reuptake inhibitors
NE	Norepinephrine
NET	Norepinephrine transporter
NOAEL	No obvious toxic reaction dose
ODV	O-desmethyl venlafaxine
PI	Principle investigator
PPS	Per Protocol set
PT	Preferred Terms
SAE	Serious adverse event
SBP	Systolic blood pressure
SDA	State Drug Administration
SDS	Sheehan disability Scale
SERT	Serotonin transporter
SNRIs	Serotonin and norepinephrine reuptake inhibitor
SOC	System organ classification
SS	Safety analysis set
SSRIs	Selective serotonin reuptake inhibitor
SUSAR	Suspicious and unexpected serious adverse reactions
$t_{1/2}$	half life
TCAs	Tricyclic antidepressants
T_{max}	Peak time
TBIL	Total bilirubin
TMS	Cranial magnetic stimulation therapy
TSH	Thyroid stimulating hormone

Summary

**Sponsor**	Shandong Luye Pharmaceutical Co., Ltd
**Inverstigate Product**	Ansofaxine hydrochloride extended-release tablets
**Title**	A Phase III, multicenter, double-blind, randomized, placebo-controlled study verify the efficacy and safety of Ansofaxine hydrochloride extended-release for major depressive disorder.
**Purposes**	**Primary Objectives：** To verify the effecicacy of Ansofaxine hydrochloride extended-release tabelet for Major depressive disorder<br>**Secondary Objectives：** To evaluate the safety of Ansofaxine hydrochloride extended-release tabelet for Major depressive disorder
**Study population**	18 ~ 65 years old patients with MDD who meet the Diagnostic and Statistical Manual of Manual Disorders, 5th Edition (DSM-5)diagnostic criteria
**Sample size**	3 groups, 186 cases/group, a total of 558 cases
**Study design**	A multicenter, randomized, double-blind and placebo-controlled phase III clinical trial to verify the efficacy and safety of Ansofaxin hydrochloride sustained-release tablets in the treatment of MDD and to provide data evidence for the approval of marketing.<br>Study consists two periods: screening period (0 to 7 days) and double-blind treatment period(8 weeks). Patients with MDD who meet the enrollment criteria will be randomized in 1:1:1 ratio to the Ansofaxin hydrochloride 80 mg group, 160 mg group and placebo group and will receive double-blind treatment for 8 weeks. Subjects will take medication as prescribed and have visits at the weekends of study week 1, 2, 4, 6 and 8.<br>The primary endpoint of efficacy is the mean change in the total scores of the Montgomery and Asberg Depression Rating Scale (MADRS)10 items from baseline at the end of treatment. The secondary endpoints of efficacy are the

	mean changes from baseline at the end of treatment in 17-item Hamilton Rating Scale for Depression (HAM-D_{17})total scores, Clinical Global Impression – global improvement (CGI- I) and severity illness (CGI-S) scores, Hamilton Anxiety Rating Scale (HAMA) scores, HAM-D_{17} factor scores, remission and response rates in MADRS, remission and response rates in HAM-D_{17} and the mean changes in the scores of SHEEHAN Disability Scale (SDS). Safety assessments included adverse events, vital signs, physical examination, laboratory tests, 12-lead ECG, Columbia-Suicide Severity Rating Scale (C-SSRS) and Arizona Sexual Experience Scale (ASEX).<br><br>A mixed model for repeated measurements (MMRM) of the change from baseline in MADRS total score will be applied. In the Model, the change from baseline in MADRS total score at each time point will be taken as dependent variable; baseline MADRS total score will be taken as covariate; treatment group, site and visit will be considered as fixed effects; individual subject will be considered as random effect. The estimate of the difference between test group and placebo at 8 weeks MADRS total score and its 95% confidence interval will be calculated.<br><br>For Safety analysis, the type, severity, frequency, and relationship with the study drug for all adverse events occurring during the trial will be described in the list.
**Study Drug**	**Study Drug:** Ansofaxin hydrochloride sustained-release tablets, strengths of 40mg and 80mg per tablet, provided by Shandong Luye Pharmaceutical Co., Ltd.<br><br>**Placebo:** placebo matching LY03005, 40 mg and 80mg per tablet, provided by Shandong Luye Pharmaceutical Co., Ltd.
**Grouping and Dosage**	**1. Group allocation**<br><br>A total of 558 subjects with MDD who meet the diagnostic criteria will be randomized to 2 treatment groups (80mg and 160mg) or placebo group, 186 subjects in each group.<br><br>**2. Dose and adminstration：**<br><br>Once a day at a regular time in the morning, taken with empty stomach or after breakfast<br><br>(1) Placebo group:<br><br>➢ 1st week: 40mg simulating agent, 1 tablet;

	➢ 2nd week: 80mg simulating agent, 1 tablet;<br>➢ 3rd to 8th week: 80mg simulating agent, 2 tablets.<br>（2）Ansofaxin hydrochloride sustained-release 80mg group (80mg/day):<br>➢ 1st week: 40mg/tablet, 1 tablet;<br>➢ 2nd week: 80mg/tablet, 1 tablet;<br>➢ 3rd to 8th week: 80mg/tablet, 1 tablet plus 1 tablet of 80mg simulating agent.<br>（3）Ansofaxin hydrochloride sustained-release 160mg group (160mg/day):<br>➢ 1st week: 40mg/tablet, 1 tablet;<br>➢ 2nd week: 80mg/tablet, 1 tablet;<br>3rd to 8th week: 80mg/tablet, 2 tablets.
**Duration**	Double-blind treatment for 8 weeks
**Frequency of visit**	At screening and the weekends of 1, 2, 4, 6 and 8 weeks after treatment.
**Inclusion Criteria**	1. Male or female aged 18 to 65 years subjects from outpatients;<br>2. Subjects currently meet the Diagnostic and Statistical Manual of Manual Disorders, fifth Edition(DSM-5) criteria for MDD with either single or recurrent episodes (296.2/296.3) without psychotic characteristics;<br>3. Subects has a total score of the Montgomery- Åsberg Depression Scale (MADRS) ≥26 points at screening;<br>4. Subects has a clinical Global Impression –severity illness (CGI-S) score≥4 points at screening;<br>5. At screening, women of childbearing age (e.g., women who have not undergone surgical sterilization or less than one year after menopause) have a negative pregnancy test result. Male and female subjects of childbearing age agree to take effective contraceptive measures during the entire study period and at least 28 days after the last dose of test drug;<br>6. Subjects voluntarily participate in the trial by signing the informed consent and are able to follow the schedule in the protocol for visits,

	treatment, laboratory tests and other research procedures.
**Exclusion criteria**	(1) Allergic or known to be allergic to venlafaxine and desvenlafaxine;<br>(2) Subjects with MDD who were not responsive to the previous venlafaxine treatment with sufficient amount and duration and to at least two different mechanisms of action antidepressants with adequate amount and duration in the past;<br>(3) There is a clear suicide attempt or behavior and score of the 10th item (suicidal ideation) in MADRS scale is 4 points or greater;<br>(4) Pregnant or lactating women,women who have a planned pregnancy in the near future;<br>(5) Subjects meet the diagnostic criteria for other psychotic disorders(except for MDD) in DSM-5, such as Schizophrenia Spectrum and Other Psychotic Disorders, Bipolar and Related Disorders, Obsessive-Compulsive and related Disorders, post-traumatic stress disorder, separation disorders, anorexia nervosa or bulimia and personality disorder;<br>(6) Subjects who meet the diagnostic criteria for substance or alcohol abuse (excluding nicotine or caffeine) 6 months prior to the screening;<br>(7) MDD secondary to other mental illnesses or physical illnesses;<br>(8) Those with a history of seizures (except for convulsions caused by febrile seizures in children);<br>(9) Receiving electroconvulsive therapy (ECT) within 3 months prior to screening or according to the investigator's judgment that ECT is currently required;<br>(10) Those who have received systematic psychotherapy (interpersonal relationship therapy, dynamic therapy, cognitive behavioral therapy) within 3 months of screening;<br>(11) Those who have received transcranial magnetic stimulation (TMS) 3 months prior to screening;<br>(12) Those who have received light therapy 2 weeks prior to screening;

	(13) Those who have stopped psychotropic drugs for less than 7 half-lives prior to study randomization (monoamine oxidase inhibitor for at least 2 weeks, fluoxetine for at least 1 month);<br>(14) Those with seriously unstable cardiovascular, liver, kidney, blood, endocrine and other physical diseases or a medical history;<br>(15) Hypertensive patients with poor blood pressure control (SBP≥140 mmHg or DBP≥90 mmHg at screening);<br>(16) There is a history of gastrointestinal disease known to interfere with drug absorption or excretion or a history of surgery known to interfere with drug absorption or excretion;<br>(17) A history of increased intraocular pressure or narrow-angle glaucoma;<br>(18) Total bilirubin (TBIL) value is 1.5 times higher, alanine aminotransferase (ALT) or aspartate aminotransferase (AST) is 2 times higher and creatinine (Cr) is 1.2 times higher than the upper limit of normal ranges. Thyroid stimulating hormone (TSH) is outside of the normal range;<br>(19) Electrocardiogram (ECG) abnormalities are clinically significant at screening and the investigators believe that it is inappropriate for the subjects to be enrolled, such as QTc interval >470 ms for male and QTc interval >480 ms for female;<br>(20) Those who have participated in other clinical trials within 3 months prior to screening;<br>(21) Those with serious acute or chronic diseases, mental illnesses or clinically significant abnormalities in laboratory tests, of which investigators believe that the subjects are not suitable for this study.
**Efficacy assessment**	**Primary endpoints：**<br>Changes from baseline in the Montgomery- Åsberg depression scales (MADRS) total scores at the end of treatment.<br>**Secondary endpoints:**<br>• Changes from baseline in the 17 items Hamilton Depression Scales

	($HAM\text{-}D_{17}$) total scores at the end of treatment.<br>• Clinical Global Impression – global improvement (CGI-I) scores at the end of treatment.<br>• Changes from baseline in the Clinical Global Impression –severity illness (CGI-S) scores at the end of treatment.<br>• Changes from baseline in the the Hamilton Anxiety Scale (HAMA) total scores and factor scores at the end of treatment.<br>• Changes from baseline in the $HAM\text{-}D_{17}$ factor scores at the end of treatment.<br>• Response rate in MADRS at the end of treatment (Response means that the reduction in MADRS score is 50% or more relative to the baseline value).<br>• Response rate in $HAM\text{-}D_{17}$ at the end of treatment (Response means the reduction in the $HAM\text{-}D_{17}$ scores is 50% or more relative to the baseline values).<br>• Remission rate in MADRS at the end of treatment (Remission refers to MADRS ≤12).<br>• Remission rate in $HAM\text{-}D_{17}$ at the end of treatment（Remission refers to $HAM\text{-}D_{17}$ ≤7）.<br>• Changes from baseline in the SHEEHAN Disability Scale (SDS) total scores at the end of treatment.
**Safety evaluation**	• Adverse events<br>• Vital signs, physical examination, laboratory tests (blood routine, urine analysis, blood biochemistry, serology), and 12-lead ECG<br>• Colombia - Suicide Severity Rating Scale (C-SSRS)<br>• Arizona Sexual Experience Scale (ASEX)
**Statistical Analysis**	Statistical analysis will be performed using SAS. Continuous data will be summarized in terms of the mean, standard deviation (SD), median, minimum and maximum. Categorical variables will be summarized in

	terms of, frequency and percentages.<br><br>For the analysis of the primary efficacy endpoint, a mixed model for repeated measurements (MMRM) of the change from baseline in MADRS total score was applied. In the Model, the change from baseline in MADRS total score at each time point will be taken as dependent variable; baseline MADRS total score will be taken as covariate; treatment group, site and visit will be considered as fixed effects; individual subject will be considered as random effect. The estimated value of week 8 MADRS total score and its 95% confidence interval in each group will be calculated. Before establishingformal model, MMRM including treatment-by-site interaction will be used to identify model applicability. If P value for the interaction is greater than 0.1, the Model will be applicable; otherwise, efficacy among sites will be considered inconsistence.<br><br>For Safety analysis, the type, severity, frequency and relationship with study drug for all TEAEs will be summarized Subjects dropped out from study due to adverse events and those with serious adverse events will be listed. Adverse events will be coded using the Medical Dictionary for Regulatory Activities (MedDRA).Shift tables will be used to summarize the changes of clinical significance evaluation(base on the evaluation of clinician) of laboratory parameters, and all abnormal parameters with clinical significance will be listed.

A Phase III Clinical trial of Ansofaxine hydrochloride extended-release tablet for Major Depressive Disorder.

—— A multicenter, double-blind, randomized, placebo-controlled study assessing the efficacy and tolerability of Ansofaxine hydrochloride extended-release for major depressive disorder.

1. Background information

Major depressive disorder (MDD) is a common and serious mental illness characterized by high incidence, high recurrence, and high disability. It is often accompanied by self-accusation, low self-esteem, loss of interest in normally enjoyable activities, low energy, and difficulty concentration. Major depressive disorder is a chronic disorder and can negatively affect a person's personal life, work life, or education, as well as sleeping, eating habits, and general health.

Currently commonly used antidepressants medication include tricyclic antidepressant (TCAs), monoamine oxidase inhibitor (MAOIs), selective serotonin reuptake inhibitor (SSRIs), serotonin and norepinephrine reuptake inhibitors (SNRIs), dopamine and norepinephrine reuptake inhibitors (NDRIs), Norepinephrine with specific serotonin antidepressants (NaSSA) and so on. As a norepinephrine (NE) and serotonin (5-HT) uptake inhibitor of venlafaxine hydrochloride (product name effexor®,1993, marketed by Wyeth) and methyl venlafaxine (product name PRISTIQ®, 2008 developed by the Wyeth) has been applicated widely in clinical, however, the effect is slow onset, cause sexual dysfunction, and unable to improve the symptom of anhedonia ect.

The Ansofaxine hydrochloride extended-release was developed by Shandong Luye Pharmaceutical Co., Ltd. Catagrory 1.1 antidepressants new drug, is *p*-hydroxybenzoic acid (PHBA) of an O-desmethylvenlafaxine (ODV), with low affinity of hydrophilic and higher solubility in fat tissue compare to ODV, which has a higher membrane penetrability. Ansofaxine hydrochloride extended-release and its metabolites ODV are distribute selectively in the target organ hypothalamus of brian with a considerable concentration after absorbed, and both of them play an important therapeutic role. Compared with PRISTIQ®, Ansofaxine hydrochloride extended-release tablets not only improve bioavailability, but also increase the inhibitory effect of dopamine (DA), which is a potential three-reuptake inhibitor, which will have the advantages of rapid onset of efficacy, improve the symptoms of anhedonia and sexual dysfunction, and also improve cognitive function, reward motivated function and goal-oriented behavior.

Pharmacological action

Ansofaxine hydrochloride extended-release tablets release the drug into the blood stream continuously. When the drug is released, both ansofaxine and its metabolite ODV enter the brain, distribute selectively in the target organ hypothalamus with comparable concentrations, and work together to achieve therapeutic effects. Ansofaxine hydrochloride displayed high affinities for the dopamine transporter (DAT), the norepinephrine transporter and the serotonin transporter (SERT). It had significant inhibitory effects on the reuptake of DA, NE and 5-HT. After administered to rats, ansofaxine hydrochloride revealed an overall effect that is characteristic of a tri-inhibitor on the reuptake of DA, NE and 5-HT. Obvious occupancies of DAT, NET and SERT by ansofaxine hydrochloride in the rat brain were also seen. A single intragastric administration of ansofaxine hydrochloride was able to: 1) significantly increase the head twitches induced by 5-hydroxytryptophan (5-HTP) at sub-threshold doses in rats; 2) suppress the decrease in body temperature induced by apomorphine hydrochloride in mice; 3) increase the toxicity of yohimbine at sublethal doses in mice; 4) reduce the immobility time in the forced swimming test performed in rats and in the tail suspension test performed in mice; 5) raise the time spent by anxious rats in the open arms of an elevated zero maze; 6) inhibit the writhing response in mice induced by acetic acid; 7) attenuate the central and peripheral pains in the formalin test conducted in mice. Continuous intragastric administration of ansofaxine hydrochloride was found to markedly inhibit the elevated spontaneous locomotor activities in rats following olfactory bulb removal; increase the consumption of sugared water; reduce circulating corticosterone levels; and increase circulating testosterone levels.

Toxicology studies

Ansofaxine hydrochloride inhibited the potassium channels of Chinese hamster ovary (CHO) cells stably expressing the *hERG* gene *in vitro*, with an IC_{50} of 2.43 μM. However, no change in the corrected QT interval (QTc) on the electrocardiography (ECG) was observed in either the safety pharmacology study or the subchronic toxicity study. Meanwhile, ODV, but not ansofaxine, was detected in the plasma of the Cynomolgus monkeys treated with ansofaxine hydrochloride extended-release tablets in the pharmacokinetic study, suggesting that ansofaxine hydrochloride has no cardiotoxicity.

Ansofaxine hydrochloride had no irritating effect on the gastrointestinal tract. A single dose of ansofaxine hydrochloride administered intragastrically to rats did not have obvious effects on the systemic functions of the body. No noticeable impact on the locomotor activities or the motor coordination was found in mice. No synergistic action with pentobarbital sodium at subthreshold doses was identified. No marked effects were observed on the blood pressure, heart rate, ECG, or breathing in the Cynomolgus monkeys under anesthesia.

In the acute toxicity study conducted in Sprague Dawley (SD) rats, a single, very high dose of ansofaxine hydrochloride resulted in the death of some animals. Some rats exhibited symptoms/signs that may be linked to the drug’s mechanism of action, including tremor/shiver, convulsion, spasm, redness of the extremities and the ears, decreased spontaneous movements,

abnormal posture, lethargy, and prostrate posture. No obvious abnormality was noted for the rats treated with ansofaxine hydrochloride at 500 mg/kg (31.3 times the maximum recommended therapeutic dose, MRTD). No abnormality was observed in the Cynomolgus monkeys intragastrically administered a single dose of ansofaxine hydrochloride at 500 mg/kg (62.5 times the MRTD).

In one of the subchronic toxicity study of rats, the animals were treated with an intragastric administration of ansofaxine hydrochloride for 28 consecutive days. At the very high dose, a few animals experienced deaths and damages to the respiratory tract. One death was observed in rats receiving a dose of 300 mg/kg (18.8 times the MRTD). These deaths were unrelated to systemic toxicities of the drug. Instead, they may be attributable to the cumulative damages resulted from the repeated inhalation of ansofaxine hydrochloride into the lungs when the drug was administered. In the animals treated at a dose of 300 mg/kg (18.8 times the MRTD) or greater, mild lesions on the eyelids, damages on the lens and vitreous opacities were observed. These damages may be associated with the activation of the 5-HT receptors resulted from the drug's inhibitory effect on 5-HT reuptake. No obvious abnormalities were identified in the animals receiving a dose of 100 mg/kg (6.3 times the MRTD). The toxicokinetic study conducted at the same time showed no obvious drug accumulation in any of the dosage groups with continuous dosing for 28 days. The no-observed-adverse-effect level (NOAEL) was 100 mg/kg, which was converted based on body surface area and equivalent to 6.3 times the MRTD.

In the subchronic toxicity study in Cynomolgus monkeys, ansofaxine hydrochloride was orally administered for 28 days continuously. Sporadic (2 out of 8), reversible thymic atrophy was observed in the animals in the 200 mg/kg dosage group (25 times the MRTD). This damage may be related to the excessive inhibition on the reuptake of NE and 5-HT caused by an overly high dose of the drug, which can lead to decreased appetite and weight loss. These appetite and weight changes can then stimulate the adrenal cortex and trigger the stress response. No obvious abnormalities were identified in the animals receiving a dose of 60 mg/kg (7.5 times the MRTD). The toxicokinetic study in these Cynomolgus monkeys showed no gender difference and no obvious drug accumulation in any of the dosage groups with continuous dosing for 28 days. The NOAEL was 60 mg/kg, which was converted based on body surface area and equivalent to 7.5 times the MRTD.

In the other subchronic toxicity study in rats, ansofaxine hydrochloride was intragastrically administered for 91 days continuously at three different dosages (30, 100, 300 mg/kg). One animal in the 300 mg/kg (18.8 times the MRTD) dosage group experienced near death that was spontaneous and unrelated to the treatment. Other than a one-time, transient ptyalism in all dosage groups and a transient, slight weight loss in the male animals in the 300 mg/kg group, no obvious abnormalities was noted in any of the following examinations or tests: food consumption, ophthalmology, hematology, blood biochemical analysis, hormone levels, urinalysis, organ weights and ratios, gross anatomy, and histopathology. The toxicokinetic study

in rats showed no obvious drug accumulation in any of the dosage groups with continuous dosing for 91 days. The NOAEL was 300 mg/kg, which was converted based on body surface area and equivalent to 18.8 times the MRTD.

In the subchronic toxicity study in Beagle dogs, ansofaxine hydrochloride extended-release tablets were orally administered for 91 days continuously at three different dosages (50, 100, 200 mg/kg). Except for transient weight loss and reduced food consumption in the female dogs in the 200 mg/kg group, no obvious abnormalities was noted in the general health of the animals or any of the following examinations or tests: ophthalmology, hematology, blood biochemical analysis, hormone levels, urinalysis, bone marrow smear, organ weights and ratios, gross anatomy, and histopathology. The toxicokinetic study in these dogs showed no obvious drug accumulation in any of the dosage groups with continuous dosing for 91 days. The NOAEL was 200 mg/kg，which was converted based on body surface area and equivalent to 37.5 times the MRTD.

The genetic toxicology study indicated that ansofaxine hydrochloride was not mutagenic and did not induce chromosomal aberration in cells, nor did it cause DNA damages or chromosomal aberration in mice.

The reproductive toxicity studies in rats did not find any *toxicologically relevant abnormalities* or drug-related toxicopathological changes under gross or microscopic examinations, except for reduced embryo implantations in the 300 mg/kg (18.8 times the MRTD) group. The NOAEL was 100 mg/kg, which was converted based on body surface area and equivalent to 6.3 times the MRTD.

The reproductive toxicity study of embryo fetal development (EFD) in rabbits found no obvious changes in pregnant animals in any dosage group (15.6, 52.0, or 156.0 mg/kg); nor did the drug have any obvious effects on the reproductive functions of the pregnant rabbits, embryo formation, or the development of embryo and fetus. The toxicokinetic study in these rabbits showed no obvious drug accumulation in the body. The NOAEL for the pregnant rabbits and for the development of embryo and fetus was 156 mg/kg, which was converted based on body surface area and equivalent to 18.8 times the MRTD.

Non-clinical pharmacokinetics

After entering the body, ansofaxine hydrochloride was rapidly metabolized into ODV. Ansofaxine was nearly undetectable in the blood. After a single dose of ansofaxine hydrochloride (4 - 16 mg/kg in the rats; 10 - 30 mg/kg in the Cynomolgus monkeys) was administered intragastrically, the C_{max} and AUC of ODV were both positively correlated with the dosages. The rate of drug clearance was similar to what was observed when equal molar doses of ODV were administered intragastrically. No gender differences were seen in the rats or the monkeys.

Compared with PRISTIQ®, the relative bioavailability of ansofaxine hydrochloride extended-release tablets was 131% (calculated based on the measured levels of the metabolite ODV). When equal molar doses of ansofaxine hydrochloride extended-release tablets and solution were

administered to the Cynomolgus monkeys orally, the tablet group showed significantly greater $t_{1/2}$ and T_{max} than the solution group, which confirms the slow-release character of the extended-release tablets. The accumulation factor after multiple dosing of ansofaxine hydrochloride extended-release tablets was 1.15, indicating no obvious accumulation of ansofaxine hydrochloride in the body.

After ansofaxine hydrochloride was administered to the rats, both ansofaxine and its metabolite ODV were detectable in tissues. Ansofaxine and ODV were rapidly distributed to a wide range of tissues, but particularly selectively to the target organ inside the brain, hypothalamus ($C_{hypothalamus} >> C_{brain}$). At 0.25 h and 1 h, the concentrations of ansofaxine and ODV in the hypothalamus were comparable. Both ansofaxine and ODV were mostly cleared from all tissues 12 hours after dosing (with concentrations less than 10% of the C_{max}), suggesting no obvious accumulation.

The study of ansofaxine hydrochloride metabolism indicated that it is first metabolized into ODV, and then is further metabolized into the same ODV metabolite as previously reported in the literature. No other metabolites were identified.

Ansofaxine hydrochloride showed no obvious inducing effect on hepatic microsomal enzymes CYP1A2, CYP2D6, CYP2C9, CYP3A4 and CYP2C19 in rats. It had no inhibitory effect on human recombinant CYP1A2, CYP 2C9, CYP 2C19, CYP 2D6 and CYP 3A4.

The urinary excretion of ODV reached a plateau 24 hours after ansofaxine hydrochloride was administered to rats (greater than 90% of the total excreted amount in 144 hours). The main excretion routes were urine and feces. About 19.5% (± 6.8%) of ODV was excreted in the free form and 75.6% (± 12.6%) was excreted in the conjugated form. The total excretion of metabolites in urine and feces was 95.1% (± 19.6%).

Clinical Pharmacology

1. Evaluation of the tolerance and pharmacokinetics of a single oral dose of ansofaxine hydrochloride extended-release tablet in healthy volunteers in a randomized, double-blind, placebo-controlled, dose-escalation study.

72 Male and female health volunteers were recurited in the clinical trial, 60 subjects were treated with an ansofaxine hydrochloride extended-release tablet and 12 subjects were treated with placebo.

In total 72 patients have been evaluated for safety, 14 subjects in ansofaxine hydrochloride extended-release group reported adverse events occurred in 20 times; advense events including nausea, dizziness, fatigue, diarrhea, ventricular arrhythmia (undiagnosed), sinus tachycardia. Nausea are the most frequently reported AEs. 9 of the incidences adverse events were considered relevent to the investigator drug. The adverse events are mild; clinical or diagnostic observations only; intervention not needed and self-recovered. There are no any new abnormal findings on

physical examination or laboratory or oxygen saturation associated with the investigational drug. In clinical studies, single doses of Ansofaxine hydrochloride extended-release in the range of 20 mg to 200 mg per day shown to be safe and well tolerated.

Single dose, dose ascending of oral administration of ansofaxine hydrochloride extended-release at 20 mg, 40 mg, 80 mg, 120 mg, 160 mg and 200mg, ansofaxine hydrochloride extended-release rapidly converted to active metabolites O-methyl venlafaxine, a very low concentration of drug prototypes can be detected in plasma and urine samples in 80-200mg dose group,

The T_{max} was 6.00 to 8.00 hours, t1/2 was 8.30 to 11.29 hours, C_{max} and AUC increased in proportion to the dose, and 50% of ansofaxine hydrochloride were renal elimination in the form of O-methyl venlafaxine.

2. Evaluation of 2- Period, 2 sequence and crossover, comparative pharmacokinetics study of ansofaxine hydrochloride extended-release tablet after fasting and after fed in healthy volunteers

12 Male and female health volunteers were recurited in the clinical trial, 6 male and 6 female. 3 subjects in ansofaxine hydrochloride group reported adverse events occurred in total 5 incidences; described as nausea, 1 adverse events were considered relevent to the investigator drug. The adverse events are mild; clinical or diagnostic observations only; intervention not needed and self-recovered. In clinical study showed well tolerated and safe with single doses of ansofaxine hydrochloride of 120 mg oral administration either with high fat meal or without food.

Oral adminstration of 120mg ansofaxine hydrochloride with food with higher C_{max} of Ansofaxine hydrochloride extended-release, later T_{max} and the bioavalibility is about 106% compared to oral administration of ansofaxine hydrochloride without food.

3. Evaluation of the tolerance and pharmacokinetics of single dose vs. multiple doses of ansofaxine hydrochloride extended-release tablet in healthy volunteers in a randomized, double-blind, placebo-controlled, dose-escalation study.

A total of 48 male and female health volunteers were recurited in the clinical trial, 40 subjects were treated with an ansofaxine hydrochloride extended release tablet and 8 subjects were treated with placebo.

In total 48 patients have been evaluated for safety, 15 subjects in ansofaxine hydrochloride extended release group reported adverse events occurred in 24 incidences; 16 of the adverse events were considered relevent to the investigator drug, most commom advense events including nausea 5 incidences, vomiting 3 incidences, diarrhea 3 incidences, and dizziness 2 incidences, larbortory abnormality included elevated of total bilirubin 2 incidences and alanine transaminase 1 incidences. The adverse events are mild; clinical or diagnostic observations only;

intervention not needed and self-recovered. There are no any new abnormal findings on physical examination, 12 lead EKG, laboratory or oxygen saturation associated with the investigational drug. In clinical studies, single doses of ansofaxine hydrochloride extended release in the range of 40 mg to 160 mg per day, once a day for consecutive 7 days shown to be safe and well tolerated.

The results for multiple oral doses of ansofaxine hydrochloride extended-release tablet showed that in a the range of 40-160mg/day, the median T_{max} was 3-6 hour and the mean $t_{1/2}$ was 9.09-10.04 hours. The systemic exposure increased slightly in subjects receiving multiple doses. The accumulation factor was 1.20. The plasma levels of ODV reached the steady state after three days of continuous dosing. The steady-state C_{max} and AUC of ODV were in proportion to the dose.

Phase II clinical trial

A multicenter, Randomized, Double-Blind, Placebo-Controlled, Parallel Group, Study in major depressive disorder subjects to find the optimal theraputic dosage, and evaluate the preliminary efficacy and safety of ansofaxine hydrochloride extended-release.

Aged 18-65 years are eligible for the study who met the Diagnostic and Statistical Manual of Mental Disorders (DSM-IV) criteria for major depressive disorder. HAM-D_{17} total score≥ 20, Eligible patients will randomaly assigned to received ansofaxine hydrochloride extended-release tablet at 40mg, 80mg, 120mg, and 160mg group or placebo- group up to 6 weeks double-blinded treatment, patients will be follow up at week 1, 2, 4, and 6. The primary efficacy measure: the 17 Hamilton Depression Scale (HAM-D_{17}); secondary efficacy measure, the Montgomery-ÅsbergDepression Rating Scale (MADRS), Hamilton Anxiety Rating Scale (HAM-A), Clinical Global Impression-severity (CGI-S), The Clinical Global Impressions Scale improvement. HAM-D_{17} and MADRS response rate and remission. Safety evaluation include assessment of adverse events, vital signs, physical exam, laboratory test, 12-lead ECG, C-SSRS and ASEX etc. Full analysis set will be used to analyse the efficacy of the study.

Result of the study: The primary efficacy measure: HAM-D_{17} total score change from the baseline. Placebo group: -9.71±6.75, Ansofaxine hydrochloride extended-release group (as a treatment group) 40mg: -12.53±5.75, 80 mg group: -12.84±6.07, 120 mg group: -12.14±7.60, 160 mg group: -13.56±5.64. The mean differences (90% confidence interval) between each dose group (40 mg, 80 mg, 120 mg and 160 mg) and placebo group were as following:group 40 mg: -2.92(-5.05,-0.80), group 80 mg: -3.08(-5.19,-0.98), group 120 mg: -2.43(-4.53,-0.33), group 160 mg: -3.69(-5.82,-1.56): The upper limit of confidence interval is less than 0, indicating that each dose of Ansofaxine hydrochloride extended-release groups were superior to the placebo group.

The secondary efficacy: MADRS total score change from baseline at week 6. Placebo group -10.92±9.38, Ansofaxine hydrochloride extended-release 40 mg group -15.39±7.78, Ansofaxine hydrochloride extended-release 80 mg group -15.30±8.90, Ansofaxine hydrochloride extended-

release 120 mg group -15.36±10.04, Ansofaxine hydrochloride extended-release 160 mg group -15.65±7.75; The mean differences (90% confidence interval) between each dose group (40 mg, 80 mg, 120 mg and 160 mg) and placebo group were as following:: group 40 mg: -4.43(-7.27,-1.59), group 80 mg: -3.99(-6.82,-1.17), group 120 mg: -3.98(-6.82,-1.15), group 160 mg: -4.57(-7.43,-1.72); the upper limit of confidence interval is less than 0**,** Ansofaxine hydrochloride extended-release groups were superior to placebo group.

The additional of secondary efficacy endpoint: change from baseline in theHamilton Anxiety Rating Scale (HAM-A), Anxiety/somatization factor items, Clinical Global Impression-severity (CGI-S), The Clinical Global Impressions Scale improvement, Response rate of 1HAM-$D_{17}$and MADRS , , and MADRS remission. Each Ansofaxine hydrochloride extended-release groups were superior to placebo group.

The safety efficacy: the advese events incidences between placebo group and treatment group at dose of 40mg, 80mg, 120mg and 160mg were 61.22 %; 65.38 %; 75 %; 70.59 % and 68.63 % respectively, there was no significant difference between groups, (SS：P = 0.6423). The advese reaction incidences between placebo group and treatment group at dose of 40mg, 80mg, 120mg, and 160mg were 38.78 %, 51.92 %, 65.38 %, 56.86 % and 62.75 %, there was significant difference between groups, (P = 0.0630).There was significant difference between groups of ansofaxine hydrochloride extended-release at dose of 80 mg and 160 mg and placebo group (P = 0.0097 and P = 0.0272), There was no significant difference between groups of ansofaxine hydrochloride extended-release at dose of 40 mg and 60 mg and placebo group.

Most of adverse events or adverse reaction were mild to moderate, clinical or diagnostic observations only; intervention not needed and self-recovered. Most commom advense events including nausea, decrease appetite, fatigue and dizziness. There were no statistically significant on the Arizona Sex Experience scale and the Columbia-Suicide severity Rating Scale (C-SSRS) between groups at each time point of follow up visit. The most common adverse events associated with drop-out were nause, headache, and dizziness. Two patients in Ansofaxine hydrochloride extended-release 40mg group were reported to have had severe adverse events: one patient had hypomania and one patient had worsing of the symptoms of depression, both of them were considered relevent to the study drug, follow up to the stable condition. No dealth occurred in this study.

Phase II clincal trial result showed: The efficacy, safety and tolerability of Ansofaxine hydrochloride extended-release tablets in the range of 40-160 mg superior to the placebo group.

This clinical trial was conducted in accordance with the clinical trial approval issued by the State Food and Drug Administration (CFDA) on June 16, 2015 (Approval No.: 2015L01158/2015L01159/2015L01160/2015L01161).

2. Objective:

The primary objective:

To e verify the efficacy of Ansofaxine hydrochloride extended-release tablets in treatment of major deppresive disorder patients.

The secondary objective:

To valuate the safety of Ansofaxine hydrochloride extended-release tablets in treatment of major deppresive disorder patients.

3. Study design reference

1. Guidance for developing antidepressant medications(2018) [1]
2. Clinical drug trial registry（2007）
3. Cood Clinical Practice (GCP)（2003）
4. Rational for study of the Ansofaxine hydrochloride extended-release tablet.
5. Pre-clinical pharmacodynamic study of Ansofaxine hydrochloride extended-release tablet
6. Pre-clinical pharmacology and toxicology study of Ansofaxine hydrochloride extended-release tablet
7. CSR of a Randomized, Double-Blind, Placebo-Controlled, Parallel Group, Single Ascending Dose Study in Healthy Subjects to Evaluate the Safety, Tolerability and Pharmacokinetics
8. CSR of two-Period, Parallel Group, Crossover Study to Evaluate the Pharmacokinetics of Ansofaxine hydrochloride extended-release under fed and fast condition in Healthy Subjects
9. CSR of a Randomized, Double-Blind, Placebo-Controlled, Parallel Group, Single and multiple of Ascending Dose Study in Healthy Subjects to Evaluate the Tolerability and Pharmacokinetics
10. CSR of a multicenter, Randomized, Double-Blind, Placebo-Controlled, Parallel Group, dose finding phase II clinical Study to assess the efficacy and safety in major depressive disorder patients .

4. Study design

4.1 Study methods

A multicenter, Randomized, Double-Blind, Placebo-Controlled phase III clinical Study design to verify the efficacy and safety of ansofaxine hydrochloride extended-release tablets in major depressive disorder.

The study will consist of screening period (0- 7 days), and double blinded treatment period (8 weeks). The first stage is screening period (0-7 days), the second stage is double-blinded treatment period. Eligible subjects will be 1:1:1 randomly assigned to receive Ansofaxine hydrochloride extended-release tablets 80mg, 160mg group or placebo group. All subjects involved with the study will remain blinded to the treatment for 8 weeks. Subjects will be treated accordingly and will follow-up on week 1, 2, 4, 6 and 8.

The primary efficacy endpoint will measure the mean score change from baseline of the Montgomery- Åsberg Depression Rating Scale (MADRS) 10 items.

The secondary efficacy endpoint will measure the mean score change from baseline of the 17 Hamilton Depression Scale (HAM-D_{17}); Clinical Global Impression-severity (CGI-S); Hamilton Anxiety Rating Scale (HAM-A);HAM-D_{17} response rate and remission rate; MADRS response rate and remission rate; Sheehan disability scale (SDS)and the mean score change of the Clinical Global Impressions-inprovement (CGI-I).Safety assessment include adverse events, vital sign, physical exam, laboratory test, 12-lead ECG, The Columbia-Suicide severity Rating Scale (C-SSRS) and Arizona Sex Experience scale (ASEX). [2] .

4.2 Placebo Rational

Based on《Guidance on treatment of patients with Major depressive disorder》(2018), Design for the experimental treatment over placebo to verify the Efficacy and Safety of Ansofaxine hydrochloride extended-release in Major Depressive Disorder. The experiment is placebo-controlled study.

4.3 Dose selection rational

Phase II clinical trial showed, the efficacy dose were in the range of 40mg/day, 80mg/day, 120mg/day, 160mg/day, of which the overall better effect dose were of the of 80 mg and 160 mg; In addition, based on the recommended dosage of the pristiq® Manual [3], daily dose were 50 mg, but the dosage of 100 mg were also approved. According to the same mole of Venlafaxine (ODV), the 80 mg Ansofaxine hydrochloride extended-release tablets are equivalent to 50 mg pristiq®, 160 mg Ansofaxine hydrochloride extended-release tablets are equivalent to 100 mg pristiq® . Therefore, The Phase III trial study dose is recommended to 80 mg and 160 mg.

4.4 Duration of the study

From《Technical guidelines for the clinical trials of antidepressant drugs released》(2018), The duration of trial is 8 weeks based on the recommendation of 6-8 weeks treatment for acute major depressive disorder.

4.5 Sample size estimate

Sample size estimate were based on the primary efficacy endpoint, MADRS total score change from baseline, based on phase II clinical trial result, a standard deviation of 9 units was selected based on the MADRS score change from baseline at the end of treatment. Statistically significant

was 5% level on two side with a power of 90%. Active treatment group was significantly different from the placebo group with a mean different of -3.5 units, Subjects were randomly 1:1:1 assigned to 80mg group, 160 group and placebo group. PASS15 software was used to calculate the sample size, 140 patients per group. Considering about 25% drop-out rate , 186 subjects were planned in each group, total 558 subjects in three groups.

4.6 Randomization method

Dynamic randomization will be applied , the randomization will be conducted by the minimization random allocation system. Pocock&Simon minimization method will be used, baseline MADRS score, age and gender are prognostic factors, and will be taken as stratification factor tomake them balance between groups, After qualified subjects recruited, The researcher who involuted in clinical trial will use the specified username and password to log into the system, filled in the screening required information, obtained the random number relevant to certain drug, and then subject will receive the drug based on the randomized number that subject had. The random list will be generated by a third party statisticians using SAS; and the statistician will conduct the procedure of drug blinding set-up and emergency envelope production.

Two copies of randomized drug blinding code shall be kept by the investigator and sponsor for each. The procedure of unblinding will be performed in two levels, first-level unblinding takes place when all data forms have been keyed into the database and database locked, allocation that predetermined by the assignments for each participant who entered into the A, B and C study group are provided, and then statistician will perform the statistical data analysis. Second level of unblinding shall be made after completing the first draft of report of the statistical analysis, and then identify the group A, B and C are corresponded to the study group of 80 mg, 160mg group and placebo group.

4.7 Blind method and design

This is a double-blind clinical trial. The study drug is produced, packaged and provided by Luye Pharmaceutical Co., Ltd., and the placebo should be in according with the study drug from the appearance, odor and packaging. The third party statistician and other staffs who do not participate in the trial are chosed to do blinding set-up according to the ramdom list; and the blinding set-up predure will be document.

The third party statistician and the engineer of randomization system who participated in the process of randimazation and blinding set-up in the trial shall not be involved in any other work related to the clinical trial and shall not disclose the blinding information to any other staff. Only the encrypted email with blind code message can be send from statistician to the randomization system engineer, and the passwords and encrypted messages shall be transmitted separately.

5. Subject selection

5.1 Inclusion criteria

1) Male or female aged 18 to 65 years subjects from outpatients;
2) Subjects currently meet the Diagnostic and Statistical Manual of Manual Disorders, fifth Edition(DSM-5) criteria for MDD with either single or recurrent episodes (296.2/296.3) without psychotic characteristics;
3) Subects has a total score of the Montgomery- Åsberg Depression Scale (MADRS) ≥26 points at screening;
4) Subects has a clinical Global Impression －severity illness (CGI-S) score≥4 points at screening;
5) At screening, women of childbearing age (e.g., women who have not undergone surgical sterilization or less than one year after menopause) have a negative pregnancy test result. Male and female subjects of childbearing age agree to take effective contraceptive measures during the entire study period and at least 28 days after the last dose of test drug;
6) Subjects voluntarily participate in the trial by signing the informed consent and are able to follow the schedule in the protocol for visits, treatment, laboratory tests and other research procedures.

5.2 Exclusion criteria

(1) Allergic or known to be allergic to venlafaxine and desvenlafaxine;

(2) Subjects with MDD who were not responsive to the previous venlafaxine treatment with sufficient amount and duration and to at least two different mechanisms of action antidepressants with adequate amount and duration in the past;

(3) There is a clear suicide attempt or behavior and score of the 10th item (suicidal ideation) in MADRS scale is 4 points or greater;

(4) Pregnant or lactating women,women who have a planned pregnancy in the near future;

(5) Subjects meet the diagnostic criteria for other psychotic disorders(except for MDD) in DSM-5, such as Schizophrenia Spectrum and Other Psychotic Disorders, Bipolar and Related Disorders, Obsessive-Compulsive and related Disorders, post-traumatic stress disorder, separation disorders, anorexia nervosa or bulimia and personality disorder;

(6) Subjects who meet the diagnostic criteria for substance or alcohol abuse (excluding nicotine or caffeine) 6 months prior to the screening;

(7) MDD secondary to other mental illnesses or physical illnesses;

(8) Those with a history of seizures (except for convulsions caused by febrile seizures in

children);

(9) Receiving electroconvulsive therapy (ECT) within 3 months prior to screening or according to the investigator's judgment that ECT is currently required;

(10) Those who have received systematic psychotherapy (interpersonal relationship therapy, dynamic therapy, cognitive behavioral therapy) within 3 months of screening;

(11) Those who have received transcranial magnetic stimulation (TMS) 3 months prior to screening;

(12) Those who have received light therapy 2 weeks prior to screening;

(13) Those who have stopped psychotropic drugs for less than 7 half-lives prior to study randomization (monoamine oxidase inhibitor for at least 2 weeks, fluoxetine for at least 1 month);

(14) Those with seriously unstable cardiovascular, liver, kidney, blood, endocrine and other physical diseases or a medical history;

(15) Hypertensive patients with poor blood pressure control (SBP⩾140 mmHg or DBP⩾90 mmHg at screening);

(16) There is a history of gastrointestinal disease known to interfere with drug absorption or excretion or a history of surgery known to interfere with drug absorption or excretion;

(17) A history of increased intraocular pressure or narrow-angle glaucoma;

(18) Total bilirubin (TBIL) value is 1.5 times higher, alanine aminotransferase (ALT) or aspartate aminotransferase (AST) is 2 times higher and creatinine (Cr) is 1.2 times higher than the upper limit of normal ranges. Thyroid stimulating hormone (TSH) is outside of the normal range;

(19) Electrocardiogram (ECG) abnormalities are clinically significant at screening and the investigators believe that it is inappropriate for the subjects to be enrolled, such as QTc interval >470 ms for male and QTc interval >480 ms for female;

(20) Those who have participated in other clinical trials within 3 months prior to screening;

(21) Those with serious acute or chronic diseases, mental illnesses or clinically significant abnormalities in laboratory tests, of which investigators believe that the subjects are not suitable for this study.

5.3 Elimination criteria

The subjects who has been selected for the clinical study, will be remove if meet one of the following criteria.

1) The subjects did not meet the inclusion criteria or met the exclusion criteria;

2) No study drug taking record;

3) There were no any record for efficacy or safety information.

5.4 Criteria for withdraw

5.4.1 The decision to allow subjects withdraw from the study will be made by the invertigator.

1) Withdraw from the study if subjects experience severe allergic reaction or severe adverse events, based on the investigator's judgement.

2) Withdraw from the study if subjects experience other severe diseases.

3) Withdraw from the study if subjects have to be unblinded.

4) Non-compliance to protocol, impact the evaluation of trial's safety and efficacy.

5) Subjects has been experienced the suicide attempt, manic or psychosis symptoms.

6) Protocol violation which impact the assessment of the study drug's safety and efficacy.

7) Withdraw from the study if subjects have positive pregnancy test.

8) Meet anyone of the criteria below.

QTc>500 ms

QTc changes from baseline > 60 ms

The above criteria are based on the average three times finding of 12 lead ECG QTc interval, for example, if ECG showed QTc interval elongation, repeat the 12 lead ECG twice during the short period of time, and calculate the average of three times QTc interval of ECG, to determine whether subjects should terminiate the study or not based on the average QTc interval.

9) Termination of the study by the sponsor or investigator's clinical judgement.

5.4.2 Subjects withdraw from the study

1) Participant request to be withdrawn from study treatment, withdrawn the informed consent.

2) Lost follow-up, (at least documented three times contact with the subjects before lost follow up)

3) Other conditions in which the subject withdrew from the trial (eg, changes in place of residence, making it impossible to continue medication and follow-up).

No matter what reason for discontinuation or withdrawal of the subject from the study, should be evaluate the safety and efficacy of the study, and the primary underlying reason should be recorded in the original document and electronic case report form (eCRF).

5.5 Rule of stopping the trial

Early Termination of the trial means that the trial prematurely suspended before completion according to the protocol.

1) Terminate the clinical trial if there were severe security issues occurred.
2) Insufficient clinical response or efficacy, no clinically significant.
3) Clinical trial design has major defects or serious protocol deviations happened lead to difficult to evaluate the efficacy of the study drug.
4) Termination of the study by the sponsor or investigator.

6. Dose regimen

6.1 Dosage forms and strengths

Ansofaxine hydrochloride extended-release Tablets

Dosage form: tablet;

Strengths: 40 mg and 80 mg;

Approval number: 20171215 (40 mg), 20171122 (80 mg);

Expire data: November 2019 (40 mg), October 2019 (80 mg);

Sponsor: Shandong Luye Pharmaceutical Co., Ltd.

Ansofaxine hydrochloride extended-release Tablet Simulator

Dosage form: tablets (Does not contain any study drug ingredients, the appearance of the placebo are as same as 40mg and 80 mg Ansofaxine hydrochloride extended-release tablets);

Strengths: 40 mg and 80 mg；

Approval number: 20171123 (40 mg), 1710241 (80 mg);

Experie data: October 2019 (40 mg), September 2019 (80 mg);

Sponsor: Shandong Luye Pharmaceutical Co., Ltd..

6.2 Study Drug packaging and labeling

Clinical trial drugs packaged were consistent with clinical trial requirements. Labelled with full prescribing information: name of drug, indication, dosage and administration, Strengths, quantity, batch number, expire data, storage and handling, sponsor, and labelled for clinical trial use only.

6.3 Drug coding

This is a multicenter, randomized, double-blind, placebo-controlled phase III clinical Study, the

statistician and the staff who not involuted in the trial will generate the randomization code based on the predetermined blind drug code. The statistician will be record the entire process of coding, including the time and place of the blind, the process of the implementation of the blind and the participants, etc.

6.4 Grouping and administration methods

Double-blind treatment period (8 weeks): The placebo group and 2 treatment group with different dose of the Ansofaxine hydrochloride extended-release tablet and drug administration are shown in Table 1.

Table 1 grouping and drug administration 1

Dose grouping	Double blind treatment	
	Time	Taking medicine
**Placebo group**	Week 1	△
	Week 2	○
	Weeks 3-8	○○
**Ansofaxine hydrochloride extended-release tablets 80 mg group**	Week 1	▲
	Week 2	●
	Weeks 3-8	●○
**Ansofaxine hydrochloride extended-release tablets 160 mg group**	Week 1	▲
	Week 2	●
	Weeks 3-8	●●

▲Ansofaxine hydrochloride extended-release tablets tablets 40 mg △ Ansofaxine hydrochloride extended-release tablets tablets simulator 40 mg

●Ansofaxine hydrochloride extended-release tablets Tablets 80 mg ○ Ansofaxine hydrochloride extended-release tablets simulator 80 mg

6.5 Dosage adjustment

Change the dosage is not allowed in the clincal trial.
The subjects may be suspended if subjects experience adverse events and was agreed to by the investigator, the subjects will get other treatment based on inverstigator's judgement.

6.6 Drug storage

Study drug must be stored at room temperature in sealed package. Study drug will be in charged by the pharmacist, subjects will be assigned to dose regimens, will receive their assigned dose and administrate, only subjects who has enrolled in the trial will be administrate the study drug.

Receive and return the drug from subjects will be documented appropriately. The investigator or its authorized personnel will check and record the study drug's appearance, storage condition, usage report, expire time, and temperature log for the storage of study drug periodically.

6.7 Drug accountability

The investigational product will be supplied by Luye Pharmaceutical Co., Ltd. Designee. The investigational products will be shipped to the pharmacist or designee only. Upon receipt of investigational product, the pharmacist or designee will verify the contents of the shipments against the packing list to ensure the quantity is correct. If quantity and conditions are acceptable, the verifier will acknowledge the receipt of the shipment by signing, the designee or pharmacist in clinical trial center are responsible to maintain accountability for all investigational product, as well as document the situation of drug supply, storage, distribution and return from the subjects in detail. Frequently verifying that actual inventory matches documented inventory. If any dispensing errors or discrepancies are discovered, the sponsor must be notified immediately and the verification and clarification must be performed.

At the end of the clinical trial, Investigational product supplies will be counted and reconciled at the site before being returned to the Luye Pharmaceutical Co., Ltd., and signature and dated.

The administration of all study drugs (**including IP**) should be recorded in the appropriate in detail including actual use amount and the planned use amount of each subject on the original records and the sections of the eCRF.

Dose compliance calculation:

Compliance = number of tablets taken/number of tablets expected to be taken × 100%.

If the percental of compliance calculated by the above formula is less than 80 or greater than 120, the subjects will be considered not to compliance the treatment.

Document and detect the reason for subject non-compliance.

6.8 Concomitant medications

At each study visit, subjects will be asked whether they have taken any medication other than the investigational product and document concomitant medications within 30 days, non-medication therapy within 90 days prior to screening.

6.8.1 Prohibited medications

The following medications and adjuctive therapy are not allowed to concomitant or combined medicine during the clinical trial.

1) Any medication for antipsychotics, antidepressants, anti-anxiety drugs, mood stabilizers and Chinese herbal preperations with a stabilization effect (including health products);

2) Electroconvulsive therapy (ETC);

3) Acupuncture and other traditional Chinese medicine;

4) Systematic psychotherapy;

5) Transcranial magnetic stimulation therapy TMS;

6) Vagus nerve stimulation;

7) Deep brain stimulation;

8) Photo therapy.

6.8.2 Allowed concomitant medications and therapy

1) Subjects are permitted to take Zaleplon, Zopiclone, Dexzopiclone, and Zolpidem who have severe insomnia, the dosage should not be more than the maximum recommended dose according to the instruction manual. To administered at bedtime. This is for short term management of insomnia, not more than two weeks for continuous taking, and less than 4 weeks in total during the period of clincal trial.

2) Subjects are permitted to have non-systemic psychotherepay for symptoms.

3) Subjects are permitted to use of any concomitant medications deemed necessary for the care of the patient who have physical diseases, keep the same medication and dosage during the clinical trial.

Concurrent medical condition that must continue to be treated with medications, or other therapy, should be recorded in the appropriate in detail on the original records and the sections of the eCRF. Such as drug's name (or other treatment name), dosage, how many times to take per day, and what time to administrate, etc.

7. Follow-up visit

The detailed follow-up schedule and exam are shown in Figure 1, the subjects can be followed

up at any time for the safety reason of the drug.

Figure 1 test flow chart

	Screening period	Baseline period	Double blind treatment phase (8 weeks)					Early termination
Visit	Visit 1<br>-7～-1 d	Visit 2<br>0d	Visit 3<br>7 ± 1d	Visit 4<br>14 ± 1d	Visit 5<br>28 ± 3d	Visit 6<br>42 ± 3d	Visit 7<br>56 ± 3d	
Informed consent	×							
Inclusion and exclusion criteria	×	×4						
General information of patients	×							
Medical history and mental symptoms	×							
DSM-5 diagnostic criteria	×							
MINI	×							
random		×						
MADRS	×	×4	×	×	×	×	×	×
HAM-D_{17}		×	×	×	×	×	×	×
HAMA		×	×	×	×	×	×	×
CGI-S	×	×4	×	×	×	×	×	×
CGI-I			×	×	×	×	×	×
SHEEHAN Disability Scale		×	×	×	×	×	×	×
Physical examination	×						×	×

	**Screening period**	**Baseline period**	**Double blind treatment phase (8 weeks)**					**Early termination**
**Visit**	**Visit 1 -7 ~ -1 d**	**Visit 2 0d**	**Visit 3 7 ± 1d**	**Visit 4 14 ± 1d**	**Visit 5 28 ± 3d**	**Visit 6 42 ± 3d**	**Visit 7 56 ± 3d**	
Vital sign[1], weight	×	×[4]	×	×	×	×	×	×
height	×							
Laboratory test	×				×		×	×
FT3, FT4 and TSH	×							
HbsAg, anti-HCV	×							
Pregnancy test [3]	×						×	×
ECG	×				×		×	×
C-SSRS	×	×[4]	×	×	×	×	×	×
ASEX		×	×	×	×	×	×	×
Adverse event record	×	×[4]	×	×	×	×	×	×
Combined medication record	×	×[4]	×	×	×	×	×	×
Drug delivery, medication record		×	×	×	×	×	×	×

Note:

1. Measurement including body temperature, respiration, pulse and blood pressure (lying position and standing position).

2. Laboratory tests for safety: blood routine including the counts of white blood cells, lymphocytes, neutrophils, eosinophils, basophils, monocytes and red blood cells, hemoglobin, hematocrit (red blood cell ratio), platelet count; urine routine including urinary white blood cells, PH value, nitrite, protein, sugar, ketone body, urobilinogen, bilirubin, urinary bilirubin, red blood cells and urine specific gravity; blood biochemistry including total bilirubin, direct bilirubin, total protein, albumin, alanine aminotransferase, aspartate aminotransferase, alkaline phosphatase, γ-glutamyl transpeptidase, lactate dehydrogenase, creatine kinase, creatine kinase isozyme, urea Nitrogen/urea, creatinine, uric acid, sodium,

potassium, chlorine, total cholesterol, triglycerides, high-density lipoprotein cholesterol, low-density lipoprotein cholesterol and fasting blood glucose; serology test including prolactin (prolactin).

3. Applicable to women of childbearing age (women who have not undergone surgical sterilization or less than 1 year after menopause).

4. If the screening is the same day as enrollment (baseline), these items need only be done once

7.1 Screening

Visit 1- -7 ~ -1 day

The purpose of the visit was screening whether the patient were eligible for the trial or not. During the time the researcher will screening the candidates:

- Sign informed consent form;
- Baseline demographics (Demographic data will include date of birth, race and height (in cm);
- Medical history taking including but not limited to diagnose of the disorder, date of first diagnosed, histology, prior treatment and medication for other diseases;
- Psychiatric evaluation: MINI-International Neuropsychology Interview (MINI);
- Major Depressive disorder is diagnosed according to the DSM-5 criteria;
- Evaluation: MADRS, CGI-S and C-SSRS;
- Physical examination: including general conditions, skin and mucous, lymph nodes, eyes, ear, nose, mouth, head and neck, thyroid, heart, lung, abdomen, spine, limbs, muscle, joint and nervous system examination;
- Vital signs: body temperature, respiratory rate, pulse and blood pressure (supine position and standing position);
- Height and weight measurement;
- 12-lead ECG examination;
- Laboratory work: including blood routine, urine analysis, blood biochemistry, serology;
- Serology screen: HBsAg, anti-HCV; FT3, FT4, TSH;
- Pregnancy test (only women of childbearing age);
- Confirm the inclusion and exclusion criteria;
- Review concomitant medications;
- Adverse event assessment.

Visit 2 - baseline period (0 days)

- Re-confirm the inclusion and exclusion criteria;
- Randomization
- Assessment: MADRS, HAM-D_{17}, CGI-S, HAMA and SDS;
- Vital signs: body temperature, respiration, pulse and blood pressure (recumbent and

standing);

- body weight;
- Evaluation: C-SSRS and ASEX;
- Combined medication record;
- Adverse event assessment;
- The test drug was distributed and recorded. Subjects will administrate the test drug on the following day (Day 1);
- Schedule the next visit and remind the subject to bring back the remaining test medications and packaging when they visit.

Note: If the screening period is the same day as the baseline period, confirmation of the inclusion and exclusion criteria, MADRS, CGI-S and C-SSRS, Vital signs, weight, combined drug records, and adverse event evaluations will only be done once.

7.2 Double blind treatment stage

All visits during the double-blind treatment will have a ± (1 ~ 3) Day visit window, which is allow slight flexibility of the time schedule of the subjects but should be follow up on a specified date as far as possible. In arranging follow-up visits, it is necessary to ensure that the total duration of treatment is followed the protocol’s trial scheme, that the follow-up visits should not be arranged on the basis of the previous visit, and that follow-up visits should be arranged according to the baseline visit.

7.2.1 Visit 3 – 1 weekend (7 ± 1 day)

- Inquire about the occurrence of adverse events;
- Ask about the combination of medications;
- Evaluation: MADRS, HAM-D_{17}, HAMA, CGI-S, CGI-I and SDS;
- Evaluation: C-SSRS and ASEX;
- Vital signs: body temperature, respiratory rate, pulse and blood pressure (recumbent and standing);
- Weight measurement;
- Recover the remaining test drugs and packaging and record, release test drugs and record;
- Schedule the next visit and remind the participants to bring back the remaining test medications and packaging when they visit.

7.2.2 Visit 4 – 2 weekends (14±1 days)

- Inquire about the occurrence of adverse events;
- Ask about the combined medication situation;
- Evaluation: MADRS, HAM-D_{17}, HAMA, CGI-S, CGI-I and SDS;
- Evaluation: C-SSRS and ASEX;
- Vital signs: body temperature, respiratory rate, pulse and blood pressure (recumbent and standing);
- Weight measurement;
- Recover the remaining test drugs and packaging and record, release test drugs and record;
- Schedule the next visit and remind the subject to bring back the remaining test medications and packaging when they visit.

7.2.3 Visit 5 – 4 weekends (28 ± 3 days)

- Inquire about the occurrence of adverse events;
- Ask about the combined medication situation;
- Evaluation: MADRS, HAM-D_{17}, HAMA, CGI-S, CGI-I and SDS;
- Evaluation: C-SSRS and ASEX;
- Vital signs: body temperature, respiratory rate, pulse and blood pressure (recumbent and standing);
- Weight measurement;
- 12-lead ECG examination;
- Laboratory work: including blood routine, urine analysis, blood biochemistry, serology;
- Recover the remaining test drugs and packaging and record, release test drugs and record;
- Schedule the next visit and remind the subject to bring back the remaining test medications and packaging when they visit.

7.2.4 Visit 6 – 6 weeks (42 ± 3 days)

- Inquire about the occurrence of adverse events;
- Ask about the combined medication situation;
- Evaluation: MADRS, HAM-D_{17}, HAMA, CGI-S, CGI-I and SDS;
- Evaluation: C-SSRS and ASEX;

- Vital signs: body temperature, respiratory rate, pulse and blood pressure (recumbent and standing);
- Weight measurement;
- Recover the remaining test drugs and packaging and record, release test drugs and record;
- Schedule the next visit and remind the subject to bring back the remaining test medications and packaging when they visit.

7.2.5 Visit 7 – 8 weekends (56 ± 3 days)

- Inquire about the occurrence of adverse events;
- Ask about the combined medication situation;
- Evaluation: MADRS, HAM-D_{17}, HAMA, CGI-S, CGI-I and SDS;
- Evaluation: C-SSRS and ASEX;
- Physical examination: including general conditions, skin and mucous, lymph nodes, eyes, ear, nose, mouth, head and neck, thyroid, heart, lung, abdomen, spine, limbs, muscle, joint and nervous system examination;
- Vital signs: body temperature, respiratory rate, pulse and blood pressure (recumbent and standing);
- Weight measurement;
- 12-lead ECG;
- Pregnancy test (only women of childbearing age);
- Laboratory work: including blood routine, urine analysis, blood biochemistry, serology;
- Recover the remaining test drug and packaging and record.

7.2.6 Early termination test

Subjects may withdraw from the study at any time according to their wishes; the researcher or sponsor may require the subject to withdraw from the study at any time for safety reasons or because the subject is not be able to compliance with the follow up visit or procedure that required by the research program. If possible, the subject’s last visit evaluation will be assessed according to the 8th weekend (visit 7).The subject's source documents and eCRF should be documented in detail for the reasons of termination.

8. Efficacy and safety evaluation

8.1 Efficacy endpoint

The primary efficacy endpoint

Changes from baseline in the Montgomery- Åsberg depression scales (MADRS) total scores at the end of treatment.

The secondary efficacy endpoint

- Changes from baseline in the 17 items Hamilton Depression Scales (HAM-D_{17}) total scores at the end of treatment.
- Clinical Global Impression – global improvement (CGI-I) scores at the end of treatment.
- Changes from baseline in the Clinical Global Impression –severity illness (CGI-S) scores at the end of treatment.
- Changes from baseline in the the Hamilton Anxiety Scale (HAMA) total scores and factor scores at the end of treatment.
- Changes from baseline in the HAM-D_{17} factor scores at the end of treatment.
- Response rate in MADRS at the end of treatment (Response means that the reduction in MADRS score is 50% or more relative to the baseline value).
- Response rate in HAM-D_{17} at the end of treatment (Response means the reduction in the HAM-D_{17} scores is 50% or more relative to the baseline values).
- Remission rate in MADRS at the end of treatment (Remission refers to MADRS ≤12).
- Remission rate in HAM-D_{17} at the end of treatment（Remission refers to HAM-D_{17} ≤7）.
- Changes from baseline in the SHEEHAN Disability Scale (SDS) total scores at the end of treatment.

8.2 Safety evaluation

- Adverse event
- Vital signs, physical examination, laboratory tests (blood routine, urine analysis, blood biochemistry, serology), 12-lead ECG
- Colombia - Suicide Severity Rating Scale (C-SSRS)
- Arizona Sexual Experience Scale (ASEX)

8.2.1 Vital signs and weight

Vital signs and weight should be checked during screening, baseline, follow up and early

termination of trials.

Weight (kg): can wear clothes and shoes, do not wear coats

Body temperature (°C)

Blood pressure (mmHg): supine position and standing position

Pulse (Times/minute): supine position and standing position

Respirtory (Times/minutes): Sitting in the state

Blood pressure and pulse measurement procedures: before measuring blood pressure, subjects should be required to not smoke or drink caffeinated beverages for at least 2 hours. Blood pressure should be measured with a mercury-based sphygmomanometer or an electronic sphygmomanometer that is suitable for the circumference of the subject's arm, and as much as possible pointed the same researcher to measure all subjects' blood pressure by the same side of arm throughout the study. For each visit, subjects should rest at least 2 minutes before measuring blood pressure (BP). 2 consecutive supine BP readings will be measured at a 2-minute interval. The supine pulse is measured in between the first BP measurement and the 2nd BP measurement, which ensures a pulse is measured within a 2-minute time period. After the 2nd supine blood pressure measurement, the subjects will rise and stand. After standing for 1 minutes, the 2 consecutive stand BP readings (2-minute interval) will be measured. The pulse in stand position will be measured at the time of after the first standing BP measurement and before the 2nd standing BP measurement, which ensures the pulse is measured within a 2-minute intervel. The systolic and diastolic boold pressure levels were measured by Korotkoff I and V (vanishing). Each subject's vital sign measurements will as much as possible to obtain at the same time point at the each follow up visit and should be measured and dertermined by the same staff.

8.2.2 Physical examination

Physical exam will be perform during the time of Screening; visit 7 day and early termination of trials, including general appears, skin, mucosa, lymph nodes, eyes, nose, mouth ears and head and neck, thyroid, heart, lungs, abdomen, spine and limbs, muscle and nerve system, and height will be perform in the screening.

8.2.3 12-lead ECG test

A 12-lead electrocardiogram will be conducted during screening, visit 5, visit 7 and early termination of the trial, the participants should have a rest at least for 5 minutes before examination.

8.2.4 Laboratory work

1) Safety labs will be drawn on screening, visit 5, visit 7 and early termination of the trial
   - Hemotology: Complete blood count with difference (CBC) (hematocrit (HCT), hemoglobin, red blood cells (RBC), Red Cell Distribution Width (RDW), Mean

Corpuscular Volume (MCV), Mean Corpuscular Hemoglobin (MCH), Mean Corpuscular Hemoglobin Concentration (MCHC), platelets, white blood cells (WBC) with absolute differential counts of neutrophils, lymphocytes, monocytes, eosinophils, and basophils).

- Urine analysis: leukocyte esterase, ph value, nitrite, protein, ketone, bilirubin, blood , urine specific gravity, Glucose, urobilinogen,
- Biochemistry: Total bilirubin, direct bilirubin, total protein, albumin, alanine transaminase, oxaloacetic Transaminase, alkaline phosphatase, gamma-transglutaminase, lactate dehydrogenase, creatine kinase, creatine kinase isoenzyme, urea nitrogen/urea, creatinine, uric acid, sodium, potassium, chlorine, total cholesterol, triglycerides, high-density lipoprotein cholesterol, low-density lipoprotein cholesterol, fasting blood sugar.
- Serological: Prolactin (prolactin).

2) Laboratory screening indicators, only during the screening, including:

- Free T3 thyroid acid, free thyroxine, thyroid stimulating hormone
- Hepatitis B Surface antigen, HCV antibody

3) Women of childbearing age will perform pregnancy tests on screening, visit 7 and early termination of trials.

All abnormal laboratory tests with clinically significant after the onset of the drug administration will be reviewed until the abnormal laboratory tests were returned to either normal or baseline or stable. If the abnormal laboratory result didn’t not return to normal or return to baseline level or stability within a reasonable period of time, then the relevant causes should be identified and the sponsor should be notified.

8.2.5 Suicide Assessment-columbia-Suicide severity Rating Scale (C-SSRS)

This scale is used for suicide risk assessment during screening, baselines, follow-up and early termination trials and should be screened using the "screening/baseline" scale and other follow-up using the "since last visit" scale.

8.2.6 Arizona Sexual Experience Scale (ASEX)

The sexual function of the subjects was evaluated at baseline, follow up visits and early termination of the trial, and their sexual function changes were evaluated. This questionnaire is completed by the clinician or the person who is the subject of the test.

If the patient meet any one of the following, the patient may be considered to have sexual dysfunction: Asex total score of ≥19, any asex scores ≥5 points, any 3 asex score ≥ 4 points. If the patient does not meet any of the above criteria, the sexual function may be considered normal.

8.2.7 Special precautions and Clinical intervention

1) Deterioration of clinical symptoms and suicide

Major depressive disorder patients at any age, whether or not take antidepressants medication, are possibly to experience the worsening of the depression symptoms, increase the risk to have

suicidal ideation, suicidal attempt or abnormal behavioral changes, the symptoms can persist until a significant remission occurs. It is known that depression per se and certain mental disorders are associated with the risk of suicide, and these mental disorders themselves are the strongest predictors of suicide.

All subjects who treated with the drug (ansofaxine hydrochloride extended-release tablet or placebo) should be closely monitored any sign of deterioration of clinical symptoms, suicidal ideation and attempt and behavioral changes, especially in the early stages of drug therapy. When adults and children who have major depressive disorder, other psychiatric or non-psychiatric disorders treated with antidepressants medications, the following unexpected symptoms may occur: such as anxiety, agitation, panic attacks, insomnia, irritability, hostility, aggression, impulsivity, sedentary (psychomotor restlessness), hypomania and mania. Although the link between the onset of these symptoms and the deterioration of depression and/or suicidal impulses is not yet clear, these symptoms may be a precursor to suicide.

Participants who have continued to deteriorate symptoms of depression, commit suicide or suicidal ideation should consider discontinuing the test drug, especially for those who have serious symptoms, or who have had a sudden onset or new onset of symptoms.

In this trial, participants who have a risk of suicide should be excluded and, on each visit, the Colombian-suicide severity rating Scale (C-SSRS) should be assessed carefully to monitor the risk of suicide.

2) QTc interval prolongation

In vitro, ansofaxine hydrochloride inhibited hERG- encoded potassium channels which potentially cause QTc interval prolongation, however in the animal experiment of ansofaxine hydrochloride didn’t showed QTc prolongation, In phase I tolerance clinical trials and phase II dose exploratory clinical trials didn’t show QTc prolongation with clinically significant as well. In order to ensure the safety of the subjects, in this experiment, we should rule out the patients who have abnormal ECG with clinical significance (especially with the prolongated QTc), and ECG examination should be performed to monitor any abnormality of QT interval during the trial.

3) Blood pressure

Experimental study on cardiovascular safety of crab monkeys showed that the study drug can increase blood pressure in crab monkeys. But in phase I tolerance clinical trials showed that there was no blood pressure changes with clinically significant, however, in phase II dose exploratory clinical trial showed that a few subjects who have increased in blood pressure. In order to ensure the safety of the subjects, the effect of study drugs on blood pressure should be concerned, blood pressure should be closely monitored in this clinical trials in the position of supine and standing during each visit. Patients who have uncontrolled hypertension should be ruled out.

9. Safety reporting and procedures

9.1 Adverse event

9.1.1 Definition of adverse events

Adverse event (AE) is defined as any reaction, side effect, or untoward event that occurs during the course of the clinical trial whether or not the event is considered related to the treatment or clinically significant.

For this clinical trial, AEs will include any events reported by the patient, any new medical conditions, symptoms, any new abnormal findings on physical examination or laboratory evaluation. Additionally, any worsening of a pre-existing condition or abnormality will also be considered as an AE.

In this trial, adverse events occurred from the time that the subject signed the informed consent form to the last visit of the end of study were recorded.

Adverse events include, but are not limited to, the following situations:

- A non-existent adverse medical event at the time of signing the informed consent, which occured in the course of the study;
- When signing the informed consent form, there is a combination of underlying diseases, aggravated in the course of the trial;
- Adverse events caused by special design of clinical study or intervention in the course of the study;
- Abnormal laboratory result with clinical significant based on the researcher's judgement.

Note: During the screening period with clinically significant laboratory abnormalities, if there is a reasonable and clear medical reasons to support the abnormalities occurred after the signing of the informed consent, then recorded as AE. If there is no medical reason to support it, it is recorded as a combined illness/ history of present illness.

9.1.2 Criteria for severity of adverse events

All AEs and clinically significant laboratory abnormalities will be graded as mild, moderate, and severe when filling out the eCRF adverse event table, the researcher will describe the extent of adverse events according to the criteri below.

Mild: asymptomatic or mild symptoms does not interfere with the subject's daily activities.

Moderate: to some extent interfere with the daily activities of the subject.

Severe: severely interferes with the subject’s daily activities.

The severe adverse events and serious adverse events are different. The severity is a category that used to rate the grade of the event, adverse events and serious adverse events can be rated as severe. Headaches, for example, can be rated as severe, but cannot be list in the SAE unless it meet to the criteria of SAE.

9.1.3 criteria forjudge the relationship between adverse events and test drugs

All adverse events should be evaluated for their relevance to the test drug, which is definite as definite, probable, possible, unlikely, unrelated to the incidence of adverse reactions.

$$\text{Adverse reaction rate} = \frac{\text{Number of cases with at least one}}{\text{Total number of cases used to assess}} \times 100\%$$

The criteria for determining the relationship between adverse events and test drugs are as follows:

Certain: evidence of the use of research drugs, the occurrence of adverse events and the use of research drugs have a reasonable time sequence, and adverse events are more reasonable to explain drug interpretation than other reasons. The drug withdrawal response was positive (adverse events disappeared after discontinuation of the drug), and repeated drug use (if feasible) was positive (reproduction).

Probable related: evidence of the use of research drugs, the occurrence of adverse events and the use of research drugs in a reasonable time sequence. Adverse events are more reasonable to explain drug interpretation than other reasons. The drug withdrawal response was positive.

Possible related: evidence of the use of the study drug, the occurrence of adverse events and the time dependence of the use of the study drug is reasonable. Adverse events can also be explained by other reasons. The drug withdrawal response was positive.

Unlikely: with evidence of the use of research drugs, the occurrence of adverse events is more reasonable to explain with other reasons. The withdrawal response was negative or unclear.

Unrelated: there is no correlation between the use of research drugs, or the timing of the use of research drugs and adverse events, or other causes that clearly lead to adverse events.

9.1.4 Record of adverse events

The adverse event record form should be filled out truthfully during the test.
Its type, degree, occurrence time, duration, treatment measures, treatment after detailed records, in the comprehensive consideration of comorbidities, combined with the use of drugs based on the evaluation of its relevance to the test drug. All physical examinations and laboratory examinations required by the test plan should be recorded in the subjects ' ECRF.
Comparing the results of physical examination, laboratory examination and pre-administration

examination results after drug administration, if the change suggested that the clinical status of the subjects deteriorated, the researcher must evaluate them to determine whether they were in line with the definition of adverse events and to record all changes that were identified as adverse event definitions in the adverse events section of eCRF.

Medical documents relating to adverse events should be recorded in the original document, including a report of laboratory examination results. Diagnostic or therapeutic invasive (such as surgery),non-invasive operations should not be credited as adverse events, and the disease that causes the operation should be documented as adverse events.Acute appendicitis, which occurs during adverse events reporting, should be reported as adverse events, and therefore appendectomy should be documented as a treatment for this adverse event.

9.1.5 Treatment and follow-up of adverse events

In the event of adverse events, whether there is a causal relationship between the incident and the test drug, should be actively dealt with. Patients who experience adverse events should be treated with acceptable clinical treatment measures.

If it is necessary to apply the medical measures rejected by the study, the applicant should be consulted whether the patient withdrew from the trial.

Adverse events should be followed up continuously until one of the following conditions is met:

1) resolved;
2) The event is stable;
3) The incident returns to the baseline level;
4) The event may be attributed to a drug other than a test drug or is unrelated to the test behavior;
5) When more information is impossible to obtain (the subject refuses to provide more information, or there is evidence that the participant is still missing after the best effort).

9.2 Serious adverse event

9.2.1 Definition of serious adverse events

A serious adverse event is defined as any adverse experience that meets any of the following criteria:

1) Results in death;
2) Is life-threatening;
3) Requires hospitalization or prolongation of existing hospitalization.
4) Results in persistent or significant disability or incapacity
5) Teratogenic, birth defects

6) Important medical events that may not result in death, be life-threatening, or require hospitalization may be considered serious when, based upon appropriate medical judgment, they may jeopardize the patient or subject and may require medical or surgical intervention to prevent one of the outcomes listed in this definition.

Note:

1) Life threatening indicate that the risk of imminent death of a serious patient, not the assumption that serious event will develop to death in the future.

2) Prolonged hospitalization or hospitalization not including the following situations:

- Management reasons for hospitalization (e.g. annual medical examinations);
- Routine admissions in ER (eg, admitted overnight for fluid resuscitation)
- Due to the chronic disease admission, there is no new adverse reactions, there is no worseing of the original disease (in order to check the chronic laboratory abnormalities);
- hospitalization was followed the protocol’s required during the clinical trial (as required by the protocol);
- Prior to the study, the subjects were already diagnosed with from some type of disease, the symptoms did not get worse, but already planned ahead to hospitalization and/or undergoing surgical procedures in the course of the study (e.g. elective cosmetic surgery)
- Hospitalization solely for the transfusion of blood products
- Hospitalization for normal delivery
- The other situation that Investigator don't think need to be hospitalized

9.2.2 Processing and recording report of serious adverse events

In the event of any serious adverse event occurring in the trial, whether the incident is related to the trial and the test drug, whether it be an initial report, a follow-up report or a summary report, the investigator should immediately evaluate the incident and complete the report form of the serious adverse event, sign and date,

Within 24 hours of being notified to Shandong Luye Pharmaceutical Co., Ltd. or designated CRO, Ethics Committee, the State Drug Administration, the National Health and Health Committee, the pilot unit of the Provincial Drug Administration department.

The relevant contact information for reporting SAE is as follows:

Reporting unit	phone	fax	public postboxes	address
Shandong Luye	010-52819238	010-52819366	clinicalsafety@luye.com	7th Floor, Block A, Aerospace Precision

Pharmaceutical Co., Ltd. or its designated CRO				Building, No. 30 Haidian South Road, Haidian District, Beijing
State Drug Administration	010-88331023<br>010-88331033<br>010-88330746	010-88363228	no	No. 2 Building, No. 26, Xuanwumen West Street, Xicheng District, Beijing, China
National Health and Wellness Committee	010-68792776<br>010-68792201	010-68792734<br>010-68792513	no	No. 38, North Lishi Road, Xicheng District, Beijing, China, National Health and Family Planning Commission, Building 2, Medical and Medical Medical Center, Medical Safety and Blood Department

9.3 Pregnancy

9.3.1 Pregnancy treatment and record report

From subjects that have signed the informed consent to the last follow-up visit, if female subjects or male subjects' partner becomes pregnant during the course of the study,the investigator or other site personnel must be informed the immediately, the investigator should record the event in the source document, and complete the pregnancy report form, signed and dated. Report to Shandong Luye Pharmaceutical Co., Ltd. Representative or the designated CRO and the Ethics Committee of the Research Center within 24 hours.

The investigator should also discuss the risk of continuing pregnancy and the effects to the fetus with the female subjects or male subjects' partners

9.3.2 Follow-up of pregnancy

All pregnancy events should be followed to the end of the pregnancy, i.e. termination of pregnancy or childbirth. if childbirth, should be follow-up to 42 days after the birth of the newborn. The investigator should keep updating the pregnancy report form, sign and dated based on any important information reported during pregnancy, and report it to Shandong Luye

Pharmaceutical Co., Ltd. or the designated CRO and the Ethics Committee of the Research Center within 24 hours.

If the subject appears to have any pregnancy-related SAE after completing the study, which is may be related to the study drug by the clincial judgement of researcher, it must be reported to Shandong Luye Pharmaceutical Co., Ltd. immediately.

Pregnancy events is not regarded as SAE, however outcome of pregnancy such as ectopic pregnancy, spontaneous abortion, intrauterine fetal demise, neonatal death, or congenital anomaly in a live born, a terminated fetus, an intrauterine fetal demise, or a neonatal death must be reported and handled as SAEs.

The outcomes of pregnancy or other medical events that meet SAE definitions should be reported and handled as SAE.

10. Unblinding

Emergency unblinding procedures: in order to protect the well being and safety of the trial subject, the coding system for the investigational medical product in blinded trials should include a unblinding code that permits rapid identification of the product in case of emergency unblinding is deemed to be necessary. Each unblinding code only can be used once and then become invalidated. There is a system in place for 24-hour cover to access the emergency unblinding. (login URL http://jm.epiedc.com) Follow the prompt operation for emergency uncovering. The investigator should use the system for emergency unblinding through the online blinding system as the main system. Each centre is also provided the sealed envelopes with appropriate code for emergency unblinded, which are kept in center. If serious adverse event or death or emergency intervention is required, the investigator in charge of the centre will report the monitor, the sponsor and the main researcher, and decide whether or not to make an emergency appearance.

Record for First-level blinded code : the first-level of drug blind code is identify the person who is assigned to the drug Group, second-level blind code is identify the person who is assigned to each dosage of drug group of the 80mg, 160mg and placebo group. Both level of blinded code will keep in sealed envelop respectively in site and sponsor.

Blind rule: Sponsors, researchers, data managers, and statisticians together under the blind to complete all research data audit and lock database,and then start to first-level unblinding, and the statisticians to perform the statistical analysis; after the completion of the preliminary draft of the statistical analysis report, perform a second-level unblinding.

Any modification of the database after the unblinding need to obtain the agreement from the principal investigator, the statisticians, the data manager and sign and data together.

11. Data management and statistical analysis

11.1 Data management

All collected data must be entry and completed by site staff to EDC system.

Completed eCRFs are required for each subject who signs an informed consent. All data will be transimited to the EDC system. Corrections are recorded in an audit trail once the the data transmit to EDC system, the new information, identification of the person making the correction, the date the correction was made, and the reason for change.

If further changes are required, the relevant monitor or data Manager will open a queries in the EDC system. The queries will be answered by the relevant staff in the research center. The name of the person who answered the question, the time and date will be tracked for audit. Once all the original data has been checked and all questions have been answered, the monitor or data manager will freeze the eCRF page

A handbook will be provided to the research center in regard how to entry the data and how to answer the queries in the EDC system. In addition, the EDC system training will be provided to the research Centre staff.

The sponsor or its designee will be permitted to review the subject's medical and hospital records pertinent to the study to ensure accuracy of the eCRFs.

The researcher (the authorized personnel) will be permitted to access the EDC system. The system is used to collect clinical research data electronically.

The EDC system access controls will be set to decide who can get into a system and what tasks they can perform, Generally, only researchers and authorized personnel can enter and correct data on eCRF.

It is necessary to correct the inconsistency of clinical data by computer logic checks as well as manual examination. The quries will be open in the system, and the site staff will be notified that new queries need to be answer online. All correction will be made directly online by the relevent researcher or authorized personnel. Source data verification and cross-check will be performed to ensure the consistency between the data entered at different time and different modules to ensure the quality of the data.

The researchers must electronically sign and data after completion.

The data management plan will be performed at the begining of the study, which will include detailed requirements for how to ensure the data are consistent and accurat, and how to clearlying the error data. The database will be updated according to signed data correction.

11.2 Statistical analysis plan

11.2.1 Statistical Analysis Set

The analysis sets defined in this study are as follows:

1) Full Analysis Set (FAS, Full Analysis Set)
2) Defined as all randomized subjects who had a baseline primary efficacy evaluation, took at least 1 dose of study drug. Subjects who take the drug which are not randomly assigned will be placed in randomly assigned group according to ITT.Per Protocol Set (PPS, Per Protocol Set)

Includes only subjects completing the the study drug treatment, without major protocol deviation, completing the primary endpoint efficacy evaluation. PPS is defined as follow PPS populations be defined as follow

- Qulifify for the inclusion criteria
- Completion of study treatment and follow up visit per protocol, fill in the content required in eCRF;
- Non comcomminitent medications which possibley affect the study drug's efficacy during the clincial trial.
- Compliance to the protocol

FAS and PPS are mainly used for efficacy analysis.

3) Safety Analysis Set (SS, Safety Set)

Includes all randomized subjects who have received at least one study drug. Subjects who take the drug which are not randomly assigned will be placed in actual treatment group according to the ASaT (Analysis as Treated) principle.

The above analysis data set will be discussed and decided by the main investigator, sponsor, statistician and data management before the database lock.

11.2.2 Statistical analysis method

11.2.2.1 General principles

All significant testing will be two-sided, unless otherwise specified. Data will be summarized using descriptive statistics number of subjects, mean, median, standard deviation, minimum, and maximum, lower quartile and uppper quartile for continuous variables and using frequency and percentage in each category for discrete variables.

All statistical analyses will be performed using SAS 9.4.

11.2.2.2 Test enrollment and completion

Summary the number of subjects who are screened, randomized and completed, the number of subjects in each statistic analysis set will be summarized , the number of subjects discontinued from study and the reason for discontinuation are analysed. Sreen falure subjects, discontinued subjects, subjects who are not entered in statistical analysis set will be listed separately.

11.2.2.3 Demographic data and baseline characteristics

Describe the demographic characteristics (age, gender, etc.), baseline information, as well as medical history, and evaluate the comparability of the baseline information among treatment groups.

11.2.2.4 Evaluation of efficacy

1) Primary endpoint analysis

MADRS score change from baseline at week 8

The Mixed-Effect Model Repeated Measure (MMRM) will be used to analyze the total score change from baseline in MADRS at week 8. The MADRS score change from baseline at different post baseline time points as dependent variable, and the baseline MADRS scale as covariance, the treatment group and site as the fixed effects, fixed effect variable and covariance are nested in study visit. and the subjects individual as random effect. In the model, the corrected mean change in MADRS total score from baseline, mean difference between each dose group and placebo and its 95% confidence interval will be calculated.. Safety analysis were performed on the safety population , consisting of all randomly assigned patients who received at least 1 dose of medication, safety evaluation including all type of adverse events, severity of the symptoms, and the frequency of occurrence during the period of study, and the relationship between advese events and investigator product will be described in the table.The applicability of the model is evaluated by analyse the interaction of the treatment group -by-site interaction, and if the p value of interaction great than 0.1, then the MMRM model can be used to analysis the change from baseline in MARDS score at week 8. Otherwise, the efficacy among sites will be considered as non-consistent. The results of MMRM will be the primary results.

In addition, changes in the total score of the MADRS scale at week 8 from baseline will also be evaluated by to ANCOVA (Analysis of Convariance) model for sensitivity analysis. The change in MADRS total score from baseline at week 8 is the dependent variable. The baseline MADRS score is covariates, and the treatment group and site are fixed effects. In the model, the corrected mean change in MADRS total score from baseline, mean difference between each dose group and placebo and its 95% confidence interval will be calculated.. In order to investigate the applicability of the model, the ANCOVA model including the treatment group-by-site interaction should be tested before the formal model is established. If the P value of interaction is great than 0.1, the model can be considered to be applicable and the formal model can be established . otherwise, efficacy among sites will be considered inconsistence. LOCF (Last Observation Carried Forward) will be used for missing data imputation.

2) Secondary endpoint analysis

Secondary endpoint will use the same method as the primary endpoint, statistically analyzed was the change from baseline in HAM-D_{17} total score, HAMA total score and SDS total score.

The change in HAM-D_{17}, HAMA, and SDS total score and each item score from baseline will be summarized , Paired t test will be used for statistical comparison. Analysis of variance (ANOVA) was used for statistical comparison of treatment groups.

The change in CGI-S score from baseline will be summarized, Wilcoxon-Signed Rank Test will be used to statistical comparison. Cochran-Mantel Haenszel test stratified by site will be used to statistical comparison.

The change in CGI-I score from baseline will be summarized, Cochran-Mantel Haenszel test (CMH-) stratified by site will be used to statistical comparison.

Summary the response rate of MADRS, HAM-D_{17}, and remission rate of MADRS, HAM-D_{17} at the different time point before and after the treatment. Clopper-Pearson was used to calculate 95% confidence interval. CMH- test stratified by site was used to statistical comparison.

11.2.2.5 Safety evaluation

Adverse events will be coded using MedDRA. The statistical analysis will be focus on treatment emergent adverse events (TEAE), The adverse events that occurred from the screening to the first dose will be presented by listing, but not included in the statistical analysis of adverse events.Adverse events mentioned in the following paragraphs are all for TEAE.

All adverse events, adverse events related to the study drug, adverse events unrelated to the study drug, serious adverse events, and adverse events leading to withdraw will be summarized, number of subjects, frequency and percentage of subjects will be calculated, Fisher exact test will be used for statistical comparison. The TEAE and related TEAE categorized by SOC and PT will be summaryized by treatment group. All adverse events, adverse events related to the study drug, adverse events unrelated to the study drug, serious adverse events, and adverse events leading to withdraw will also be presented by listings.

Shift tables will be used to summarize the clinical significance evaluation of laboratory and ECG parameters, and all abnormal parameters with clinical significance will be listed.

11.2.2.6 Multiplicity

In order to control the type I error in the multiple comparisons, a step-down process was used to compare the change in MADRStotal score from baseline at week 8 between each dose group (80mg, 160mg) with placebo, which is that comparison was performed for the 160 mg group first and, if the different is statistically significant, the comparison will be performed subsequently in the 80mg group. Otherwise, no comparison will be performed in the 80mg group.

The result for safety evaluation, P-value is the nominal P value, P-value is used to describe the relationship between safety endpoint and treatment groups and will not be used for formal

statistical inference.

11.2.2.7 Processing of missing data

The Mixed-Effect Model Repeated Measure (MMRM) will be used to analysis the total score of MADRS, HAM-D_{17}, HAMA and SDS for missing data. The LOCF methods was used to impute the missing data and ANCOVA model was used for sensitivity analysis.

For MADRS response rate, HAM-D_{17} response rate, MADRS remission rate and HAM-D_{17} remission rate, the predicted value from MMRM model will be used to imput the missing data, and this analysis will be considered as primary analysis results.

The analysis results of the MADRS response rate, HAM-D_{17} response rate, MADRS remission rate, HAM-D_{17} remission rate based on the LOCF imputation will be considered as sensitivity analysis.

The rest of endpoints efficacy evaluation, including HAM-D_{17} single item scores, HAMA single item scores, CGS-S score, CGS-I score, the LOCF method was used to impute the missing data.

This studywill not perform any missing data imputation for safety endpoint.

11.2.2.8 Interim analysis

The clinical trial will not perform interim data analysis

11.2.2.9 Data Monitoring Committee

The data monitoring committee will not be set up for this clinical trial.

12 Quality control and assurance

1) This clinical trial will be conducted in a certified clinical drug trial facility. Researchers who have the enriched knowedge and experience that required for this clinical trial and are relatively stable in conduct the trial.

2) Before the start of the experiment, all the researchers who participated in the clincal trial were received the training of the protocol plan, how to implement the study, procedures, and other skills that needed .

3) Before the initial of the test, all the staff who involved in the trial will received the training of how to evaluate the psychiatric interview form to ensure the consistency of the score assessment.

4) Explain the study in detail to the subjects, to ensure that subject fully understand the significance of the study, the importance of actively cooperate with the clincal study and be compliance with the treatment according to the protocol.

5) Prohibit other drugs intake or treatments that may influence the outcome of the trial.

6) The laboratory has established standard operating procedures and quality control procedures for experimental.

7) Quality control (QC) procedures and quality assurance system will be implemented and followed by sponsor and researcher.

8) Clinical site monitoring is conducted to ensure that the reported trial data are accurate, complete, and verifiable to ensure the source document are consistent with the eCRF, and that the conduct of the trial is in compliance with the currently approved protocol/amendment(s), with ICH GCP, and with applicable regulatory requirement(s).

9) During the test, the monitors will regularly monitor the progress and quality of the test to ensure that the test is carried out according to the protocol and checks the eCRF to ensure consistency with the original data.

10) Study drug must be stored at room temperature in sealed package and the counter is locked, The study drug is kept by the drug manager, the remaining drugs are stored separately and the remaining amount is registered and returned to the Luye Pharmaceutical Co., Ltd., at the end of the trial.

11) The designee will management source document, data, and responsible for the correction of instruments etc.

13 Ethics requirements

13.1 Guidance of Ethical requirements

This clinical trial will be implemented in accordance with the Helsinki Declaration and GCP requirements. The protocol must be approved by the Ethics committee before it can be implemented. The researchers will ensure that the clinical trials comply with the laws, regulations, scientific and ethical standards of the People's Republic of China on medical trials.

13.2 Informed consent

Subjects entering the trial (screening or any other research-related activity) are required to sign a written informed consent form. The investigator will fully explain to each subject the information related to the test drug, the nature, purpose, procedures, observations, tests performed, possible risks and benefits, including possible adverse reactions and The subject’s rights and obligations, give the subject sufficient time to consider the relevant content of the trial in order to decide whether to participate. The subject’s personal information will be kept strictly confidential, and the subject’s identity, privacy and test results will not be disclosed unless required by law.

13.3 Early termination test

This clinical trial will be terminated if the frequency and severity of adverse events suggest that the test drug is potentially hazardous to human health. The sponsor, the ethics committee and the relevant management department should be reported before termination.

14 Summary report

After all the cases in this clinical trial are completed, statistical analysis of all the data by the statisticians is presented, and a summary report is provided to the unit of the study leader(principle investigator).

15 Revision of the Study plan

If this protocol is found to be necessary for revision in the experiment, the principle investigator and the sponsor should be consulted and approved by both parties. After the proposal has been revised, it should be re-reported to the ethics Committee for filing/approval.
If important new information is found to be involved in the test drug, a written amendment of the informed consent should be sent to the ethics Committee for approval and the subject's informed consent will be obtained again.

16 Paper publication

The investigator may publish the results of the clinical trial after the end of the clinical trial, subject to the prior written consent of the sponsor, for protecting the intellectual property information owned by the sponsor or its representatives. For multi-center clinical trials, the primary investigator at each center must ensure that data from a single research center cannot be published or published in advance until the full clinical trial results are published.

The sponsor or his representative should not limit or hinder the researcher from publishing or publishing clinical trial results

17 Data saving

The investigator should agree to keep all research data, including the subject’s original records, informed consent, eCRF, detailed records of drug distribution, etc., and should be kept at least for 5 years after the end of the clinical trial. Researchers must notify Shandong Luye Pharmaceutical Co., Ltd. before destroying any clinical research records. If the researcher wishes to move the research record to another location, Shandong Luye Pharmaceutical Co., Ltd. must be notified in advance. If the researcher cannot guarantee that the research organization will require such archiving for some or all of the documents, the researcher and Shandong Luye

Pharmaceutical Co., Ltd. must make special arrangements to place the documents in a sealed container to another place.

18 references

[1]. State administration of Food and Drug Administration. Guiding principles for clinical trials in the treatment of depressive drugs 2018

[2]. Li Huafang. The clinical study of psychotropic drugs is a common scale. Shanghai Science and Technology Education press.2011

Wyeth Pharmaceuticals Inc. High lights of prescribing information: prescribing information for Pristiq (Ansofaxine hydrochloride extended-release).

https://www.accessdata.fda.gov/drugsatfda_docs/label/2018/021992s042lbl.pdf

Annex 1: Montgomery- Åsberg Depression Scale (MADRS)

Note: Scoring needs to be based on clinical interviews, and interviews are transferred from a broad-based questioning question about symptoms to more detailed questions that can accurately score severity. The grading staff must determine if the score is at a certain rating level (0, 2, 4, 6), or between levels (1, 3, 5), and then write a score in the score space on the right.

project	Grading	score
**Sadness of appearance**<br>Refers to disappointment, frustration, and despair in terms of speech, expression, and posture (more important than the usual low level of depression). Scored according to the degree of depression observed and the degree of ”happy to get up.”	0 = no sadness<br>1<br>2=It looks frustrating, but it is not difficult to be happy.<br>3<br>4=Most of the time looks sad and unpleasant<br>5<br>6=It seems very sad all day, extremely depressed	|_|
**2. Sad experience**<br>Refers to the subjective experience of depression, whether it is reflected in appearance, including depression, frustration, disappointment, or feeling helpless and hopeless. It is assessed by its intensity, duration, and the extent to which the emotion is affected by the event.	0=Occasional sadness, consistent with the environment<br>1<br>2= There is sadness or low mood, but there is no difficulty in getting happy.<br>3<br>4= deeply sad or depressed, but the mood can still be affected by the external environment<br>5<br>6=Continuous grief experience or depression	|_|
**3. Inner tension**<br>Uncomfortable, nervous, nervous, nervous, and uneasy, until fear, fear, or extreme pain. Evaluate according to the degree, frequency, duration and extent of the comfort warrants required by the participant.	0=Calm, occasionally inner tension<br>1<br>2=Occasionally uneasy and unspeakable discomfort<br>3<br>4=Continuous inner tension, or intermittent panic, although difficult, but patients can still control<br>5<br>6=Fear and extreme pain that cannot be restrained, extremely frightened	|_|
**4. Reduced sleep**<br>Refers to a reduction in the depth or duration of sleep experienced subjective	0=sleep as usual<br>1<br>2=It is difficult to fall asleep or sleep time is slightly reduced, or sleep is shallow, sleep	|_|

experience compared to normal sleep during health.	Wake up<br>3<br>4 = sleep reduction or sleep interruption for at least 2h<br>5<br>6=The total daily sleep time does not exceed 2-3h	
**5. Loss of appetite**<br>It means that the appetite has decreased compared with when it is healthy. Score according to the degree of loss of appetite or the degree of need to force yourself to eat.	0 = normal appetite or enhancement<br>1<br>2 = mild loss of appetite<br>3<br>4 = no appetite, food and tasteless<br>5<br>6=Requires others to persuade to eat	|_|
**6. Difficulty in concentration**<br>It is difficult to concentrate on thinking until it is impossible to concentrate on thinking. Score according to the degree, frequency and range of difficulty of concentration.	0=Attention without difficulty<br>1<br>2=Occasionally it is difficult to concentrate<br>3<br>4=It is difficult to concentrate and keep thinking, so that reading or talking ability is reduced<br>5<br>6=It is extremely difficult to read or talk	|_|
**Burnout**<br>Refers to the difficulty of starting a daily activity, or starting and slowing down.	0 = there is almost no difficulty in starting, no delay<br>1<br>2=It is difficult to start<br>3<br>4=It is difficult to carry out simple daily activities, and it takes a lot of effort to complete<br>5<br>6= There is no spirit at all, no one can help with anything.	|_|
**8. Feeling can't**<br>Refers to the subjective lack of interest in the surrounding environment or activities of original interest, and the ability to produce appropriate emotional responses to surrounding things or people.	0=The interest in people and things around is normal<br>1<br>2=Lower ability to feel everyday interest<br>3<br>4= Losing interest in things around; lacking affection for friends and acquaintances<br>5<br>6=Emotional numbness, can not experience anger, sadness or pleasure; and no friends or relatives<br>Emotional, painful	|_|
**9. Pessimistic thinking**<br>Thoughts such as humiliation, inferiority, self-	0=no pessimism<br>1<br>2=There are failures, self-blame ideas or self-deprecation<br>3	|_|

blame, self-sin, remorse, and self-destruction.	4 = Lasting self-blame, or there is still a sense of guilt and sin that is still understandable,<br>Growing pessimistic about the future<br>5<br>6= Self-destruction, remorse, and sinister reverie, ridiculous and unshakeable<br>Self-condemnation	
**10. Suicidal ideation**<br>It means that life is worthless, it is better to die, with suicidal thoughts and suicide preparation. The suicide attempt itself does not affect the score.	0=none<br>1<br>2= Tired of life, occasional suicidal thoughts<br>3<br>4=I think it might be better to die, often have suicidal thoughts; and think suicide is a kind of<br>Possible self-solving, but no specific suicide plans and plans<br>5<br>6= Have a clear plan to wait for an opportunity to commit suicide; actively prepare for suicide	|_|
**Total score|_|_|**		

Annex 2: 17 Hamilton Depression Scale (HAM-D_{17})

Note: Most projects use a 5-level scale of 0 to 4 points. The standards at each level are: (0) no; (1) mild; (2) moderate; (3) severe; (4) extremely severe. A few projects use a 3-point scale of 0 to 2 points. The grading criteria are: (0) none; (1) light to moderate; (2) severe			
project			score
1	Depressed mood (sad, hopeless, helpless, worthless)	0 = asymptomatic; 1 = only when asked;<br>2 = spontaneously expressed in the conversation;<br>3 = This emotion is expressed non-verbally (eg through expressions, gestures, voices, and desires to cry);<br>4=The patient's self-speech and non-verbal expressions are almost completely expressed as such emotions	|_|
2	Guilty	0 = asymptomatic; 1 = blame yourself, feel yourself disappointing others;<br>2 = think that you have committed a crime, or repeatedly think about past mistakes or mistakes;<br>3=I think that my current illness is a punishment for my own mistakes, or a guilty conscience;<br>4=Hearing accusations or condemning voices and/or threatening illusions	|_|

3	suicide	0 = asymptomatic; 1 = feels meaningless to live;<br>2=I hope that I am already dead or often think about things related to death;<br>3 = negative concept (suicidal thoughts) or suicidal gestures;<br>4=Suicide attempt (4 points for any serious attempt)	|_|
4	Difficulty falling asleep	0=No difficulty in falling asleep;<br>1 = The main complaint sometimes has difficulty falling asleep (the bed can still not sleep after 30 minutes);<br>2=The main complaint is that it is difficult to fall asleep every night.	|_|
5	Not sleeping well	0 = asymptomatic;<br>1=The chief complaint is that the night sleep is shallow and many nightmares;<br>2=Wake up in the middle of the night (before 12 pm) - 2 points for any wake up (not including<br>To the restroom)	|_|
6	Wake up early	0 = asymptomatic;<br>1 = wake up early, but can fall asleep again;<br>2=Can't fall asleep after waking up early	|_|
7	Work and activities	0 = asymptomatic;<br>1= Feeling powerless, tired or weak in activities, work or hobbies;<br>2= Losing interest in activities, hobbies or work – patients directly or indirectly expressed listlessness and excellence<br>Indecisive and hesitant (feeling that you must force yourself to work or be active);<br>3=The activity time is reduced or the efficiency is reduced. The resident's daily ward activity (hospital labor or entertainment) is not<br>Including the daily affairs of the ward for less than 3 hours;<br>4=The work is stopped due to the current illness. The resident does not participate in any activities other than the daily affairs of the ward, or can not complete the daily affairs of the ward without the help of others.	|_|
8	Hysteresis (meaning slow thinking and speech, difficulty concentrating, active decline)	0 = normal speech and thinking;<br>1 = mild lag was found during mental examination;<br>2=Significant delay in mental examination;<br>3 = difficulty in mental examination;<br>4=completely stiff	|_|
9	intense	0 = asymptomatic;<br>1=Uncertain;<br>2=Playing hands, hair, etc.;<br>3=Walking around, can't sit still;<br>4=Hands, biting nails, pulling hair, biting lips	|_|
10	Mental anxiety	0 = asymptomatic;	|_|

		1 = subjective tension and irritability;<br>2= worried about small things;<br>3=Expressions and speeches reveal obvious concerns;<br>4=Undoubtedly showing fear	
11	Physical anxiety (physiological symptoms of anxiety such as dry mouth, shortness of breath, indigestion, diarrhea, abdominal cramps, belching, palpitations, headache, excessive ventilation, sighing, frequent urination, sweating)	0 = asymptomatic;<br>1 = mild;<br>2 = moderate;<br>3=severe;<br>4= seriously affecting life and activities	|_|
12	Gastrointestinal symptoms	0 = asymptomatic;<br>1 = loss of appetite, but do not need encouragement to eat on their own, the abdomen is heavy;<br>2 = need to be urged by others; request or need to apply laxatives or laxatives, or drugs for gastrointestinal symptoms	|_|
13	Systemic symptoms	0 = asymptomatic;<br>1 = heavy feelings on the limbs, back or head; back pain, headache, muscle pain; general weakness and fatigue;<br>2 = 2 points for any obvious symptoms	|_|
14	Sexual symptoms (loss of libido, menstrual disorders)	0 = asymptomatic;<br>1 = mild;<br>2=severe	|_|
15	Suspected disease	0 = asymptomatic; 1 = excessive attention to (physical health); 2 = repeated consideration of health issues;<br>3 = Frequent complaints, requests for help, etc.; 4 = Suspected delusions	|_|
16	Weight loss	0 = no weight loss;<br>1 = there may be weight loss associated with the current disease;<br>2 = definitely weight loss (according to the patient);<br>3=not evaluated	|_|
17	Self-awareness	0 = know that you are sick, showing depression;<br>1=I know I am sick, but I blame it for poor food, environmental problems, work, viral infection or<br>Need to rest, etc.;<br>2= completely denied the disease	|_|
Total score|_|_|			

Annex 3: Clinical Total Impression Scale (CGI)

I. Severity of disease (CGI-S)

Combine your overall clinical experience with this particular population, how serious is the patient’s condition at this time?

0=Unrated 4=Moderately sick

1 = normal, completely disease free 5 = obviously sick

2 = marginal psychosis 6 = serious illness

3=Slightly sick 7=The disease is extremely serious

II. Overall Progress (CGI-I)

At your discretion, this improvement is entirely due to the treatment of the drug. How does the patient’s condition change compared to when the patient was enrolled in the interview?

0 = not rated 4 = no change

1=Advance is very obvious 5=Slightly worse

2=Advance is obvious 6=Significantly worsened

3=Slight progress 7=Deterioration is very obvious

Annex 4: Hamilton Anxiety Scale (HAMA)

All items used a 5-level scale of 0 to 4 points. The standards at each level are: (0) non-existent; (1) mild; (2) moderate; (3) severe; (4) very serious.		
**project**		**score**
Anxious mood	Worried, the hunch has the worst thing to happen, feels scared, easy to provoke	|_|
Nervous	Feelings of tension, fatigue, shocking reaction, tearing after shaking, shaking, feeling uneasy, not relaxing	|_|

Fear	Fear of darkness, strangers, alone, animals, cars, or people	|_|
4. Insomnia	Difficult to fall asleep, easy to wake up, sleep poorly and feel tired after waking up, dreaming, nightmares, night terrors	|_|
5. Memory or attention disorder	Intention can not concentrate, memory is poor	|_|
6. Depressed mood	Losing interest, lack of pleasure in past hobbies, depression, early waking, squatting	|_|
7. Muscle symptoms	Muscle soreness, muscle twitching, inflexible activity, myoclonic convulsions, molars, trembling, increased muscle tone	|_|
8. Feeling symptoms	Tinnitus, blurred vision, cold and fever, weakness, tingling	|_|
9. Cardiovascular symptoms	Tachycardia, heart palpitations, chest pain, vascular motility, fainting, heartbeat leakage	|_|
10. Respiratory symptoms	Chest tightness or tightness, suffocation, sigh, difficulty breathing	|_|
11. Gastrointestinal symptoms	Dysphagia, abdominal pain, burning, bloating, nausea, vomiting, bowel, diarrhea, weight loss, constipation	|_|
12. Genitour urinary symptoms	Frequent urination, urgency, amenorrhea, menstrual flow, cold, premature ejaculation, loss of libido, impotence	|_|
13. Autonomic symptoms	Dry mouth, flushing, paleness, sweating, dizziness, tension headache, hair erection	|_|
14. Performance during the meeting	Sitting restless, irritated or pacing, shaking hands, frowning, stiff expression, sighing or shortness of breath, pale, swallowing from time to time, etc.	|_|
**Total score|_|_|**		

Annex 5: SHEEHAN Disability Scale (SDS)

A brief assessment of disability and defects by patient self-assessment
Please mark only one circle for each rating scale.

Work * or school

Symptoms affect your work or study：

Not at all Mild Moderate Obvious Extrem

⓪ ← ------①----②----③-----④----⑤----⑥-----⑦----⑧----⑨--- → ⑩

☐Over the past 1 weeks, I have not worked or studied for reasons related to illness.

* Work includes paid work, unpaid work or training。

Social life

Symptoms affect your social life or leisure activities

Not at all Mild Moderate Obvious Exterm

⓪ ← ------①----②----③-----④----⑤----⑥-----⑦----⑧----⑨--- → ⑩

Family life or family responsibilities

Symptoms affect your family life or your family's contribution

Not at all Mild Moderate Obvious Exterm

⓪ ← ------①----②----③-----④----⑤----⑥-----⑦----⑧----⑨--- → ⑩

Number of days absent from absence

In the past week, how many days have you been unable to complete your normal day-to-day duties due to your symptoms?

Inefficient days

In the past week, even if you go to school or go to work every day, how many days are there because your symptoms make you feel obstructed and the efficiency is reduced?

Appendix

SDS assessment instructions

This scale is a self-rating scale, which is mainly used to assess the functional status and efficacy of patients with mood disorders or anxiety disorders. A survey of patients attending primary care centres showed that the internal consistency of the scale was good with an alpha coefficient of 0.83.

Evaluate the situation of the last 7 days before the interview.

The assessment includes work, social life, and family life; related items include days of absence and days of inefficiency.

Each entry is divided into 11 levels of 0 to 10 points, with a total score of 0 to 30 points.

Annex 6: Colombia - Suicide Severity Rating Scale (C-SSRS)

7-1 Colombia - Suicide Severity Rating Scale - Screening/Baseline

Suicidal ideation		
Ask questions 1 and 2. If the answer to both questions is no, please go to the "Suicide Behavior" section. If the answer to question 2 is "yes", please ask questions 3, 4, 5. If the answer to question 1 and/or the answer to question 2 is yes, complete the "Ideas Strength" section below.	In a lifetime: the time when you most want to commit suicide	The past 6 months
**1 hope to die**<br>The subject acknowledged the idea of hoping to die or not to live, or the idea of waking up after falling asleep.<br>**Have you ever wanted to die or hope to wake up after you fall asleep?**<br>If yes, please describe:	whether<br>☐ ☐	whether<br>☐ ☐
**2 Unspecific active suicide ideas**<br>During the assessment period, the idea of ending my life and committing suicide was general and not specific (such as " I thought about suicide"), but did not think about the way suicide, related methods, intentions or plans.<br>**Have you ever thought of suicide?**<br>If yes, please describe:	whether<br>☐ ☐	whether<br>☐ ☐
**3 Active suicidal ideation with method (unplanned) but no intention of action**<br>During the assessment period, the subject acknowledged the idea of suicide and thought about at least one suicide method, which is different from the specific plan that has time, place or specific method (such as thinking about suicide but not specific) s plan This includes someone who would say, "I thought about taking too much medication, but I never had a specific plan for when, where and actually to actually do it... and I will never do it."<br>**Have you thought about how you would commit suicide?**<br>If yes, please describe:	whether<br>☐ ☐	whether<br>☐ ☐
**4 Active suicidal ideation with intention of action but no specific plan**	whether	whether

There is an idea of active suicide, and the subject says that there is an intention to implement these ideas, rather than "I have these ideas but I will definitely not implement them."<br>**Have you had these ideas and have had the intent to implement them?**<br>If yes, please describe:	☐ ☐	☐ ☐
**5 Active suicidal ideation with specific plans and intentions**<br>The details of the plan for suicidal thoughts have been fully or partially developed and the subject has an intent to execute the plan.<br>**Have you started to develop or have a detailed suicide plan?**<br>**Do you want to implement this plan?**<br>If yes, please describe:	whether<br>☐ ☐	whether<br>☐ ☐
Intensity of mind		
The following characteristics regarding the most intense intentions should be assessed (eg, from 1 to 5 above, 1 is the lightest and 5 is the heaviest). Ask him (her) the time he most wants to commit suicide.<br><br>In life: the strongest idea: ___________ _________________<br>Type number (1~5) meaning description<br>The past 6 months: the strongest idea: ___________ _________________<br>Type number (1~5) meaning description	Strongest	Stronges t
**frequency**<br>**How many times have you generated these ideas?**<br>(1) less than 1 time in 1 week<br>(2) Once a week<br>(3) 2 to 5 times a week<br>(4) 1 time per day or almost once a day<br>(5) Multiple times a day	_______	_______
**duration**<br>**How long will these thoughts last when you have suicidal thoughts?**<br>(1) Short time - just a few seconds or minutes<br>(2) less than 1h/period of time<br>(3) 1~4h/long time<br>(4) 4~8h/almost 1d	_______	_______

(5) more than 8h/lasting or continuous time		
**Controllability**<br>**If you want, can you stop suicide or hope to die?**<br>(1) can easily control these ideas<br>(2) can control these ideas, almost no difficulty<br>(3) can control these ideas, but some difficulties<br>(4) can control these ideas, but it is very difficult<br>(5) can′t control these ideas<br>(0) don′t try to control these ideas	_______	_______
**Stopping factor**<br>**Is there any factor - someone or something (such as family, religion, pain of death, etc.) - has stopped your thoughts of wishing to die or committing suicide?**<br>(1) The suppression factor has indeed stopped your suicide attempt<br>(2) The stopping factor may have stopped you<br>(3) Is it uncertain whether the stopping factor has stopped you?<br>(4) The stopping factor probably has not stopped you<br>(5) The suppression factor has never stopped you.<br>(0) Not applicable	_______	_______
**Causes of suicidal ideation**<br>**What are the reasons for your hopes of dying or committing suicide? Is it to end the pain or to end your feelings at the time (in other words, this pain or your feelings at the time prevents you from continuing to endure), or to arouse his attention, reaction or revenge? Or both?**<br>(1) solely to attract the attention, reaction or revenge of others<br>(2) mainly to attract the attention, reaction or revenge of others<br>(3) It is to attract the attention, reaction or revenge of others, and to end or stop the pain.<br>(4) Mainly to end or stop the pain (you can′t continue to endure this pain or the feeling at the time)<br>(5) It is all about ending or stopping the pain (you can′t continue to endure this pain or the feeling at the time)<br>(0) Not applicable	_______	_______
**Suicidal behavior**<br>(As long as these suicidal behaviors are independent events, check all the content that matches: all types must be asked)	In life	The past 6 months

**Actual attempt:**<br>The result of the behavior is that there is a potential self-injury and at least some thoughts about wanting to die. Behavior is to some extent considered a way of suicide, and the intention is not necessarily 100%. If there is any intention or desire to die and is accompanied by action, then this behavior can be considered as an actual suicide attempt. There is no need to have damage or injury, just the potential for injury or injury. If someone pulls the trigger after putting the muzzle in the mouth, but the gun is bad and does not cause damage, it will also be considered an actual suicide attempt.<br>Inferred intent: Even if the individual denies the intention or desire to die, clinical inference can be made based on the behavior or the specific situation. For example, if a very lethal behavior is obviously not an accident, then it can be inferred that it is suicide rather than other intentions (such as shooting the head, jumping off the window of a high floor, etc.). Similarly, if someone denies the intention to die, but if the person knows that his behavior is fatal, then this intention can also be inferred.<br>**Have you tried suicide?**<br>**Have you done anything to hurt yourself?**<br>**Have you done dangerous things that could lead to your own death?**<br>**What have you done?**<br>**Have you used _______ as a way to end your life?**<br>**When you _____, do you want to die (even if there is only one such idea)?**<br>**Or have you thought that you might die because of ___________?**<br>**Or are you doing this purely for other reasons, without any intention of suicide (such as to release stress, feel better, gain sympathy, or make other things happen)? (self-injury without suicidal intentions)**<br>If yes, please describe:<br><br>Has the subject had a suicidal suicidal behavior?	whether<br>□ □<br><br>Total attempts<br>_______<br><br>whether<br>□ □	whether<br>□ □<br><br>Total attempts<br>_______<br><br>whether<br>□ □
**Interrupted attempt**<br>When someone starts a potential self-injury, they are interrupted by (an external factor) (if this is not the case, an actual suicide attempt will occur).<br>Overdose: Someone has a pill in his hand but is prevented from swallowing. Once the pill is swallowed, this becomes a suicide attempt rather than an interrupted suicide attempt. Shooting: Someone is pointing at himself with a gun, the gun is	whether<br>□ □<br><br>Total	whether<br>□ □<br><br>Total

taken away by Someone else or I don′t know why I can′t pull the trigger. Once the person pulls the trigger, even if the gun is misfiring, this is a suicide attempt. Jumping from the building: Someone is ready to take off, but Is caught and taken away from the window sill. Hanging: Someone puts the rope around his neck, but it is blocked if it is not suspended.<br>**Is there a time when you start doing something to end your life, but someone or something stopped you before you actually started?**<br>If yes, please describe:		attempts ________	attempts ________
**Give up**<br>When someone starts taking steps to try to commit suicide, they stop self-destructive behavior before actually committing suicide. This is similar to an interrupted attempt, except that the person′s suicide attempt is stopped by himself, not by others.<br>**Is there a time when you start doing something to end your life, but before you actually start, do you stop this behavior?**<br>If yes, please describe:		whether ☐ ☐<br>Total attempts ________	whether ☐ ☐<br>Total attempts ________
**Prepared action or behavior**<br>Actions or preparations for upcoming suicide attempts, including anything beyond the scope of speech and thinking. For example, develop specific methods (such as buying medicines, buying guns) or preparing for suicide (such as giving things away and writing suicides).<br>**Have you taken action for suicide attempts or prepared suicides (eg, collecting medicines, getting guns, giving valuables or writing suicides)?**<br>If yes, please describe:		whether ☐ ☐	whether ☐ ☐
**Suicidal behavior**<br>Did suicidal behavior occur during the assessment period?		whether ☐ ☐	whether ☐ ☐
**Only answer the actual suicide attempt**	Last suicide attempt	The most deadly suicide attempt	Initial/first suicide attempt

**Actual fatality, physical damage:**<br>0 No physical damage or very light body damage (such as epidermal abrasions)<br>1 Mild physical injury (such as speechlessness, burns, mild bleeding, sprains).<br>2 Moderate physical injury; need medical attention (such as conscious but sleepy, some reactions, second degree burns, large blood vessel bleeding).<br>3 moderate to severe physical injury; hospitalization is required and may be intensive care (eg, third-degree burns that are in a state of drowsiness, good reflexes, 20% of the body area, massive hemorrhage but recoverable, severe fractures).<br>4 Severe physical injury requires hospitalization and intensive care (such as being in a state of drowsiness and losing reflex function, a third degree burn of more than 20% of the body area, a large amount of blood loss, and unstable vital signs and serious damage to vital parts).<br>5 death	date	_______	_______	_______
	Input code#	_______	_______	_______
**Potentially fatal: answer only if the actual fatality = 0**<br>The actual suicide attempt may be fatal if it does not cause physical damage (the following example does not cause actual physical damage, but may have a very serious fatality: the trigger is pulled after the muzzle is placed in the mouth, but because the gun is not misfiring Causes physical damage; lies on rails that are about to come by train, but is pulled out of the rails before being run over by the train).<br>0 behavior may not cause harm<br>1 Behavior may cause harm but will not cause death<br>2 Despite medical care, behavior can still lead to death	date	_______	_______	_______
	Input code#	_______	_______	_______

7-2 Colombia - Suicide Severity Scale - since the last visit

Suicidal ideation	
Ask questions 1 and 2. If the answer to both questions is no, please go to the "Suicide Behavior" section. If the answer to question 2 is "yes", please ask questions 3, 4, 5. If the answer to question 1 and/or the answer to question 2 is yes, complete the "Ideas Strength" section below.	Since the last visit
**1 hope to die**<br>The subject acknowledged the idea of hoping to die or not to live, or the idea of waking up after falling asleep.<br>**Have you ever wanted to die or hope to wake up after you fall asleep?**<br>If yes, please describe:	whether<br>☐ ☐
**2 Unspecific active suicide ideas**<br>During the assessment period, the idea of ending my life and committing suicide was general and not specific (such as "I thought about suicide"), but did not think about the way suicide, related methods, intentions or plans.<br>**Have you ever thought of suicide?**<br>If yes, please describe:	whether<br>☐ ☐
**3 Active suicidal ideation with method (unplanned) but no intention of action**<br>During the assessment period, the subject acknowledged the idea of suicide and thought about at least one suicide method, which is different from the specific plan that has time, place or specific method (such as thinking about suicide but not specific) s plan). This includes someone who would say, "I thought about taking too much medication, but I never had a specific plan for when, where, and how to actually do it... and I will never do it."<br>**Have you thought about how you would commit suicide?**<br>If yes, please describe:	whether<br>☐ ☐
**4 Active suicidal ideation with intention of action but no specific plan**<br>There is an idea of active suicide, and the subject says that there is an intention to implement these ideas, rather than "I have these ideas but I will definitely not implement them."<br>**Have you had these ideas and have had the intent to implement them?**<br>If yes, please describe:	whether<br>☐ ☐

**5 Active suicidal ideation with specific plans and intentions**<br>The details of the plan for suicidal thoughts have been fully or partially developed and the subject has an intent to execute the plan.<br>**Have you started to develop or have a detailed suicide plan?**<br>**Do you want to implement this plan?**<br>If yes, please describe:	whether<br>☐ ☐
Intensity of mind	
The following characteristics regarding the most intense intentions should be assessed (eg, from 1 to 5 above, 1 is the lightest and 5 is the heaviest). Ask him (her) the time he most wants to commit suicide.<br>The strongest idea: ___________ ______________________<br>Type number (1~5) meaning description	Strongest
**frequency**<br>**How many times have you generated these ideas?**<br>(1) less than 1 time in 1 week<br>(2) Once a week<br>(3) 2 to 5 times a week<br>(4) 1 time per day or almost once a day<br>(5) Multiple times a day	_______
**duration**<br>**How long will these thoughts last when you have suicidal thoughts?**<br>(1) Short time - just a few seconds or minutes<br>(2) less than 1h/period of time<br>(3) 1~4h/long time<br>(4) 4~8h/almost 1d<br>(5) more than 8h/lasting or continuous time	_______
**Controllability**<br>**If you want, can you stop suicide or hope to die?**<br>(1) can easily control these ideas<br>(2) can control these ideas, almost no difficulty<br>(3) can control these ideas, but some difficulties<br>(4) can control these ideas, but it is very difficult<br>(5) can′t control these ideas<br>(0) don′t try to control these ideas	_______

**Stopping factor**<br>**Is there any factor - someone or something (such as family, religion, pain of death, etc.) - has stopped your thoughts of wishing to die or committing suicide?**<br>(1) The suppression factor has indeed stopped your suicide attempt<br>(2) The stopping factor may have stopped you<br>(3) Is it uncertain whether the stopping factor has stopped you?<br>(4) The stopping factor probably has not stopped you<br>(5) The suppression factor has never stopped you.<br>(0) Not applicable	________
**Causes of suicidal ideation**<br>**What are the reasons for your hopes of dying or committing suicide? Is it to end the pain or to end your feelings at the time (in other words, this pain or your feelings at the time prevents you from continuing to endure), or to arouse his attention, reaction or revenge? Or both?**<br>(1) solely to attract the attention, reaction or revenge of others<br>(2) mainly to attract the attention, reaction or revenge of others<br>(3) It is to attract the attention, reaction or revenge of others, and to end or stop the pain.<br>(4) Mainly to end or stop the pain (you can′t continue to endure this pain or the feeling at the time)<br>(5) It is all about ending or stopping the pain (you can′t continue to endure this pain or the feeling at the time)<br>(0) Not applicable	________
**Suicidal behavior**<br>(As long as these suicidal behaviors are independent events, check all the content that matches: all types must be asked)	Since the last visit
**Actual attempt:**<br>The result of the behavior is that there is a potential self-injury and at least some thoughts about wanting to die. Behavior is to some extent considered a way of suicide, and the intention is not necessarily 100%. If there is any intention or desire to die and is accompanied by action, then this behavior can be considered as an actual suicide attempt. There is no need to have damage or injury, just the potential for injury or injury. If someone pulls the trigger after putting the muzzle in the mouth, but the gun is bad and does not cause damage, it will also be considered an actual suicide attempt.<br>Inferred intent: Even if the individual denies the intention or desire to die, clinical inference can be made based on the behavior or the specific situation.	whether<br>☐ ☐<br><br>Total attempts<br>________

For example, if a very lethal behavior is obviously not an accident, then it can be inferred that it is suicide rather than other intentions (such as shooting the head, jumping off the window of a high floor, etc.). Similarly, if someone denies the intention to die, but if the person knows that his behavior is fatal, then this intention can also be inferred.<br>**Have you tried suicide?**<br>**Have you done anything to hurt yourself?**<br>**Have you done dangerous things that could lead to your own death?**<br>**What have you done?**<br>**Have you used _______ as a way to end your life?**<br>**When you _____, do you want to die (even if there is only one such idea)?**<br>**Or have you thought that you might die because of ___________?**<br>**Or are you doing this purely for other reasons, without any intention of suicide (such as to release stress, feel better, gain sympathy, or make other things happen)? (self-injury without suicidal intentions)**<br>If yes, please describe:<br><br>Has the subject had a suicidal suicidal behavior?	whether<br>☐ ☐
**Interrupted attempt**<br>When someone starts a potential self-injury, they are interrupted by (an external factor) (if this is not the case, an actual suicide attempt will occur).<br>Overdose: Someone has a pill in his hand but is prevented from swallowing. Once the pill is swallowed, this becomes a suicide attempt rather than an interrupted suicide attempt. Shooting: Someone is pointing at himself with a gun, the gun is taken away by someone else or I don′t know why I can′t pull the trigger. Once the person pulls the trigger, even if the gun is misfiring, this is a suicide attempt. Jumping from the building: Someone is ready to take off, but is caught and taken away from the window sill. Hanging: Someone puts the rope around his neck, but it is blocked if it is not suspended.<br>**Is there a time when you start doing something to end your life, but someone or something stopped you before you actually started?**<br>If yes, please describe:	whether<br>☐ ☐<br><br>Total attempts<br>_______

**Give up**<br>When someone starts taking steps to try to commit suicide, they stop self-destructive behavior before actually committing suicide. This is similar to an interrupted attempt, except that the person's suicide attempt is stopped by himself, not by others.<br>**Is there a time when you start doing something to end your life, but before you actually start, do you stop this behavior?**<br>If yes, please describe:	whether<br>☐ ☐<br><br>Total attempts<br>_______
**Prepared action or behavior**<br>Actions or preparations for upcoming suicide attempts, including anything beyond the scope of speech and thinking. For example, develop specific methods (such as buying medicines, buying guns) or preparing for suicide (such as giving things away and writing suicides).<br>**Have you taken action for suicide attempts or prepared suicides (eg, collecting medicines, getting guns, giving valuables or writing suicides)?**<br>If yes, please describe:	whether<br>☐ ☐
**Suicidal behavior**<br>Did suicidal behavior occur during the assessment period?	whether<br>☐ ☐
**suicide**	whether<br>☐ ☐
**Only answer the actual suicide attempt**	The most deadly suicide attempt
**Actual fatality, physical damage:**<br>0 No physical damage or very light body damage (such as epidermal abrasions)<br>1 Mild physical injury (such as speechlessness, burns, mild bleeding, sprains).<br>2 Moderate physical injury; need medical attention (such as conscious but sleepy, some reactions, second degree burns, large blood vessel bleeding).<br>3 moderate to severe physical injury; hospitalization is required and may be intensive care (eg, third-degree burns that are in a state of drowsiness, good reflexes, 20% of the body area, massive hemorrhage but recoverable, severe fractures).<br>4 Severe physical injury requires hospitalization and intensive care (such as being in a state of drowsiness and losing reflex function, a third degree burn of more than 20% of the body area, a large amount of blood loss, and unstable vital signs and serious	Input code#<br>_______

damage to vital parts).<br>5 death	
**Potentially fatal: answer only if the actual fatality = 0**<br>The actual suicide attempt may be fatal if it does not cause physical damage (the following example does not cause actual physical damage, but may have a very serious fatality: the trigger is pulled after the muzzle is placed in the mouth, but because the gun is not misfiring Causes physical damage; lies on rails that are about to come by train, but is pulled out of the rails before being run over by the train).<br>0 behavior may not cause harm<br>1 Behavior may cause harm but will not cause death<br>2 Despite medical care, behavior can still lead to death	Input code#<br>_______

Annex 7: Arizona Sexual Experience Scale (ASEX)

Arizona Sexual Experience Scale (ASEX) - Male

For each entry, please indicate your overall level over the past week, including today.	**Score**
1. How strong is your sexual desire?<br>1 very strong 2 very strong 3 some strong 4 some weak 5 very weak 6 no sexual desire	|_|
2. Is your libido easily evoked (ignited)?<br>1 very easy 2 is easy 3 is easy 4 is difficult 1 very difficult 6 never evoked	|_|
3. Can you easily get and keep an erection?<br>1 very easy 2 very easy 3 some easy 4 some difficult 5 very difficult 6 never get	|_|
4. Can you easily reach orgasm?<br>1 very easy 2 very easy 3 some easy 4 some difficult 5 very difficult 6 never reached orgasm	|_|
5. Are you satisfied with your orgasm?<br>1 very satisfied 2 very satisfied 3 some satisfied 4 some dissatisfied 5 very dissatisfied 6 can not reach orgasm	|_|
**Total score|_|_|**	

Arizona Sexual Experience Scale (ASEX) - Women

For each entry, please indicate your overall level over the past week, including today.	score
1. How strong is your sexual desire?<br>1 very strong 2 very strong 3 some strong 4 some weak 5 very weak 6 no sexual desire	|_|
2. Is your libido easily evoked (ignited)?<br>1 very easy 2 very easy 3 some easy 4 some difficult 5 very difficult 6 never evoked	|_|
3. Is your vagina easy to get wet during sex?<br>1 very easy 2 very easy 3 some easy 4 some difficult 5 very difficult 6 never become	|_|
4. Can you easily reach orgasm?<br>1 very easy 2 very easy 3 some easy 4 some difficult 5 very difficult 6 never reached orgasm	|_|
5. Are you satisfied with your orgasm?<br>1 very satisfied 2 very satisfied 3 some satisfied 4 some dissatisfied 5 very dissatisfied 6 can not reach orgasm	|_|
**Total score|_|_|**	

Annex 8: Diagnostic Criteria for Depressive Disorders in the Diagnostic and Statistical Manual of Mental Disorders, 5th Edition (DSM-5)

A. During the same 2 weeks period, 5 or more of the following symptoms appear, showing different changes compared to the previous function, at least 1 of which is 1. Mental depression or 2. Loss of interest or pleasure.

Note: Does not include those symptoms that can be clearly attributed to other physical illnesses.

The mood is depressed most of the day, either by subjective experience (eg, feeling sad or empty), or by others (eg, dark tears).

(1) Depressed most of the time, most of the time, can be subjective reports (for example, feeling sad, empty, hopeless), or other people’ s observations (for example, showing tears) (Note: children and adolescents may behave as a state of mind Irritating).

(2) For almost all of the day or day, there is a significant reduction in interest or fun for all or almost all activities (either subjective or observational).

(3) Significant weight loss, or significant weight gain (eg, 5% change in body weight over one month), or almost daily loss of appetite, or increase in appetite (Note: children can show up as they should not increase) body weight).

(4) Insomnia or excessive sleep almost every day.

(5) Almost every day, there is psychomotor agitation or delay (observed by others, not just subjectively experienced restlessness or dullness).

(6) Fatigue or lack of energy almost every day.

(7) I feel that I am worthless every day, or that I feel guilty (to the extent of delusion) that is excessive and inappropriate (not just because I am sick or guilty).

(8) Almost every day there is a decline or hesitancy in thinking or concentration (either subjective or other).

(9) Repeated thoughts of death (not just fear of death), repeated suicidal ideas without specific plans, or some kind of suicide attempt, or a specific plan to commit suicide.

B. These symptoms cause clinically meaningful pain or cause damage to social, professional or other important functions.

C. These symptoms cannot be attributed to the physiological effects of a substance or other physical illnesses.

Note: Diagnostic criteria A – C constitute a major depressive episode.

Note: Responses to major loss (eg, loss of pain, economic bankruptcy, loss of natural disasters, severe physical illness or disability) may include the symptoms listed in Diagnostic C: such as strong grief, immersion in loss, Insomnia, loss of appetite and weight loss, these symptoms can be similar to depressive episodes. Although such symptoms are understandable or responsive to loss, in addition to the normal response to major loss, careful consideration should be given to the possibility of major depressive episodes. This decision must be based on personal history and a cultural norm that expresses pain in the context of loss to make clinical judgments.

D. The presence of this major depressive episode cannot be better explained by schizoaffective disorders, schizophrenia, schizophrenia-like disorders, delusional disorders, or other specific or unspecified schizophrenia spectrums and other psychotic disorders. .

E. From a manic episode or a mild manic episode.

Note: This exclusion does not apply if all manic or maddening episodes are caused by substance abuse or due to the physiological effects of other physical illnesses.

Annex 9: Concise International Neuropsychology Interview (MINI)

Concise International Neuropsychology Interview Chinese Edition

MINI - INTERNATIONAL NEUROPSYCHIATRIC INTERVIEW

(MINI Chinese version)

Author:

United States: D. Sheehan, J. Janavs, R. Baker, K. Harnett-Sheehan, E. Knapp, M. Sheehan

University of South Florida- Tampa

France: Y. Lecrubier, E. Weiller, T. Hergueta, P. Amorim, LI Bonora., JP Lepine

Hospital de la Salpetriere - Paris

Main translation: Si Tianmei

Persons involved in the Chinese translation of the discussion (by last name strokes): Kong Qingmei, Liu Qi, Chen Jingxu, Su Yunai, Zhang Hongyan, Zhang Weihua, Dang Weimin, Cheng Jia, Dong Wentian

Proofreading: Professor Shu Liang

Peking University Mental Health Research Institute

Translation and proofreading time: June 2005 - August 2006

Translator’s note

The MINI-International Neuropsychiatric Interview (MINI) is a simple, effective and reliable structured interview tool developed by Sheehan and Lecrubier. It is mainly used for screening and diagnosis of the Diagnostic and Statistical Manual of Mental Disorders. Sixteen I-Psychiatric Disorders and a Personality Disorder, including 130 questions, in the Fourth Edition (DSM-IV) and the International Handbook of Statistical Classification of Mental Disorders (ICD-10). As with the Fixed Clinical Examination Patient Edition (SCID-P) and the Composite International Diagnostic Interview Form (CIDI), each diagnosis in the MINI is a question group, and most of the diagnoses have screening problems that exclude diagnosis. There have been studies comparing the reliability and validity of MINI with SCID-P and CIDI, and the results show that MINI has a very acceptable reliability and validity score. MINI has been translated into a variety of languages and is widely used in clinical trials and clinical practice.

In recent years, China has been increasingly involved in international clinical research. Based on this, after obtaining the consent of the original author, we translated the MINI English version 5.0.0 (2004) into a Chinese version and evaluated the reliability and validity. The results show that the Chinese version of the MINI Chinese version has a high degree of consistency in the diagnosis of depressive episodes, anxiety disorders, substance dependence, and psychotic disorders with the diagnosis made with SCID-P (Si Tianmei, et al. 2009). The reliability and validity of the Chinese version of MINI in the diagnosis of manic episodes, eating disorders, antisocial personality disorder, and post-traumatic stress disorder remain to be studied.

The use of MINI ensures the accuracy and consistency of the diagnostic process, and can reveal potential psychiatric comorbidities. Because the interview process is short, the questions are concise, and easily accepted by patients, they can be used in clinical practice. I sincerely hope that this tool will help doctors’ clinical practice and research.

Due to the limited level, there may be deficiencies in the translation. I hope that I will be tested and corrected in future use. I hope everyone will criticize and correct.

M.I.N.I diagnostic record table

Patient Name: ______________________ Numbering: ____________________
Date of birth: _________________________ Rating start time: ____________________
Rating person name: ______________________ End of assessment: ____________________
Rating date: ______________________ Evaluation time: ____________________

Question group	time limit	Standards compliant	DSM-IV	ICD-10
A depression (MAJOR DEPRESSIVE EPISODE)<br>A’ Depression with depression characteristics (optional)<br>【MDE WITH MELANCHOLIC FEATURES (optional)】	Current illness (last 2 weeks)<br>Past episode<br>Current illness (last 2 weeks)	☐<br>☐<br>☐	296.20-296.26 single time<br>296.30-296.36 recurrence<br>296.20-296.26 single time<br>296.30-296.36 recurrence	F32.x<br>F33.x<br>F32.x<br>F33.x
B bad mood (DYSTHYMIA)	Current illness (last 2 years)	☐	300.4	F34.1
C Suicide (SUICIDALITY)	Current illness (last month)	☐		
D (light) manic episode [(HYPO) MANIC EPISODE]	Now suffering<br>+ past episodes	☐<br>☐	296.00-296.06	F30.x<br>-F31.9
E Panic disorder (PANIC DISORDER)	Current illness (last month)<br>+ lifetime	☐<br>☐	300.01/300.21	F40.01<br>-F41.0
F place phobia (AGORAPHOBIA)	Now suffering	☐	300.22	F40.00
G Social phobia (social anxiety disorder)<br>[SOCIAL PHOBIA (Social Anxiety Disorder)]	Current illness (last month)	☐	300.23	F40.1
H OCD<br>(OBSESSIVE-COMPULSIVE DISORDER)	Current illness (last month)	☐	300.3	F42.8
I Post-traumatic stress disorder (optional)<br>[POSTTRAUMATIC STRESS DISORDER (optional)]	Current illness (last month)	☐	309.81	F43.1
J alcohol abuse or alcohol dependence<br>(ALCOHOL ABUSE AND DEPENDENCE)	Current illness (last 12 months)	☐	303.9/305.00	F10.2x<br>/F10.1
K Non-alcoholic psychoactive substance use disorder<br>(NON-ALCOHOL PSYCHOACTIVE SUBSTANCE USE DISORDERS)	Current illness (last 12 months)	☐	304.00-.90/<br>305.20-.90	F11.00<br>-F19.1<br>/F11.2<br>/F19.1
L Psychiatric Disorder (PSYCHOTIC DISORDER)	Lifetime +<br>Now suffering	☐<br>☐	295.10-295.90/<br>297.1/297.3/<br>293.81/293.82/<br>293.89/298.8/298.9	F20.xx<br>-F29

M Anorexia nervosa (ANOREXIA NERVOSA)	Current illness (last 3 months)	☐	307.1	F50.0
N neurogenic bulimia (BULIMIA NERVOSA)	Current illness (last 3 months)	☐	307.51	F50.2
O generalized anxiety disorder (GENERALIZED ANXIETY DISORDER)	Current illness (last 3 months)	☐	300.02	F41.1
P Antisocial personality disorder (optional) [ANTISOCIAL PERSONALITY DISORDER (optional)]	lifelong	☐	301.7	F60.2

Guidance

The Concise International Neuropsychiatric Interview (M.I.N.I) is a quick, easy, and reliable structured interview that includes 16 Axis I mental disorders in DSM-IV and ICD-10. After a simple training, the scale can be used by clinicians, but non-professionals need in-depth training if they conduct interviews.

● Interview:

In order to make the interview as brief as possible, before the formal interview, please tell the patient that you will conduct a formal clinical interview, ask some concise and specific questions about the psychological condition, and ask the patient to answer with “yes” or “no”.

● Conventional form

The M.I.N.I is divided into several question groups, identified by letters, and each letter or question group corresponds to a diagnostic category.

The beginning of each question group (except for the group of psychiatric disorders) lists the screening questions that correspond to the main symptom criteria for the disease in the **Black box shadow**.

At the end of each question group, the doctor indicates in the diagnostic box whether the patient meets the diagnostic criteria.

● Provisions:

Sentences written in ordinary Song dynasty should be accurately read to the patient to standardize the diagnostic evaluation process.

Sentences written in *italics* do not need to be read to the patient. This is a guide to the examiner to help the examiner score during the diagnosis.

Sentences written in **bold** indicate the time frame of the survey, and the examiner should read as many times as possible. Only symptoms that occur within the time frame of the survey should be considered in scoring.

The sentence (underlined in brackets) is a clinical example of the symptom that helps to further explain the problem and can be read to the patient.

The answer with an arrow (➔) above indicates that the criteria necessary for a diagnosis are not

met. In this case, the inspector should go back to the end of the question group, circle ”No” in all the diagnostic boxes, and then proceed to the next question group. ➔
When a word is separated by a slash ”/”, the interviewer should read only those symptoms that the patient has (eg, question A3).

- **Assessment guidance:**

All questions read out must be assessed. On the right side of each question, circle ”yes” or ”no”. The doctor should ensure that the patient understands all aspects of the problem (such as time frame, frequency of occurrence, severity, and/or other choices).

For any questions or suggestions that require MINI training or updated MINI information, please contact the following person:

Si Tianmei si-tianmei@163.com

Shu Liang shu-liang@126.com

A. Depressive episode (MAJOR DEPRESSIVE EPISODE)

(➔refers to: go to the diagnosis box, circle "No" on the top of the corresponding diagnosis, and then go to the next question group.)

A1	In the last two weeks, have you felt depressed or depressed for most of the day?	No	Yes
A2	In the last two weeks, have you lost interest or pleasure for what you like on weekdays?	No	Yes
	Is the A1 or A2 code ”yes”?	➔ No	Yes

A3	**In the last two weeks, when you feel depressed and/or lose interest:**		
	a. Do you have a loss of appetite or increase almost every day? Or even if you don’t deliberately diet, but the weight (body weight) declines or gains weight? (eg, weight changes exceed 5%, if a person with a weight of 70kg, the weight changes more than 3.5kg in a month). If any question answers ”yes”, the code ”yes”±	No	Yes
	b. Do you have difficulty sleeping almost every night? (difficulty falling asleep, easy to wake up at night, wake up early or sleep too much)	No	Yes
	c. Do you speak or move slowly every day, or feel annoyed, restless, and difficult to sit still?	No	Yes
	d. Do you feel tired or lose energy almost every day?	No	Yes
	e. Do you have a sense of worthlessness or unrealistic guilt almost every day?	No	Yes

	f. Are you hard to concentrate or hesitate almost every day, and it is difficult to make a decision?	No	Yes
	g. Do you want to hurt yourself, commit suicide or want to die?	No	Yes
	Are there three or more responses to the code "Yes"?<br>***or***<br>*If A1 or A2 encodes "No", does A3 have more than four answer codes "Yes"?*	**No Yes**<br>**Depressive episode Now suffering**	
	If the patient currently meets the criteria for depression:		
A4	a. In your life, have you been there for a while, more than two weeks, you feel depressed or depressed, or lost interest in most things, and there are many other problems we mentioned above?	➔<br>No	Yes
	b. Is there more than 2 months between your last depressive episode and this depressive episode, do you not feel depressed or lose interest?<br>*(If the patient is positive for depression (A3= "Yes"), please continue to ask the following questions ;)*	**No Yes**<br>**Depressive episode Recurrent**	

A'. Depression with depression characteristics (optional)

(MAJOR DEPRESSIVE EPISODE WITH MELANCHOLIC FEATURES)

(➔ refers to: go to the diagnosis box, circle "No" on the top of the corresponding diagnosis, and then go to the next question group.)

A5	*a. Is the A2 code "yes"?*	No	Yes
	b. At this time of the most serious depressive episode, are you not responding to anything you like and what makes you feel good? If "No": If something good happens, still can't make you happy? Even a short happy?	No	Yes
	Is there a code "yes" in A5a or A5b?	➔<br>No	Yes
A6	**In the last two weeks, when you feel depressed and/or lose interest:**		
	a. Does your depression feeling and bereavement react differently? The mourning reaction is the sad feeling that occurs when a loved one dies.	No	Yes
	b. Do you feel the morning is heavier almost every day?	No	Yes
	c. Are you waking up almost 2 hours earlier than usual in the morning and can't sleep anymore?	No	Yes
	d. Is A3a coded "yes"? (lack of appetite or weight loss)	No	Yes
	e. Is the A3c code "yes"?	No	Yes
	f. Do you feel excessive and unrealistic guilt?	No	Yes

A6 has 3 or more answer codes "Yes"?

**No Yes**<br>**Depressive episode**<br>**Melancholy**<br>**Now suffering**

B. Bad mood (DYSTHYMIA)

(➔ refers to: go to the diagnosis box, circle "No" on the top of the corresponding diagnosis, and then go to the next question group.)

If the patient's current symptoms meet the diagnostic criteria for depression, skip this question group.

B1	In the last two years, have you been sad, depressed or depressed most of the time?	➔ No	Yes
B2	Do you feel good when you have been feeling sad for two months or more?	No	➔ Yes
B3	**During the time you feel sad, is it most of the time:**		
	a. Has your appetite changed significantly?	No	Yes
	c. Do you have difficulty falling asleep or sleeping too much?	No	Yes
	d. Are you tired or lacking energy?	No	Yes
	e. Do you feel lost confidence?	No	Yes
	f. Is it difficult for you to concentrate or hesitate to make a decision?	No	Yes
	g. Do you feel that life has no hope?	No	Yes
	B3 has two or more answers coded "yes"?	➔ No	Yes
B4	Do these depressive symptoms make you feel very distressed or hinder your social, professional function, or affect other important functions?	➔ No	Yes
	Is the B4 code "yes"?	**No**	**Yes**

No Yes
Bad mood
Now suffering

C. Suicide (SUICIDALITY)

	**In the last month:**			**score**
C1	Do you think it will be better to die or hope that you are dead?	No	Yes	1
C2	Do you want to hurt yourself?	No	Yes	2
C3	Do you want to commit suicide?	No	Yes	6
C4	Do you have a suicide plan?	No	Yes	10
C5	Are you trying to commit suicide?	No	Yes	10
	**In your life:**	No	Yes	
C6	Have you tried suicide?	No	Yes	4

Is there at least one of the above codes "yes"?

If yes, please assign a score of "Yes" to C1-C6, score according to the score on the right side, and then total the scores. According to the total score, (according to the following criteria):

No Yes

Suicide risk

Now suffering

Low risk €1-5 points:

Medium risk €6-9 points:

High risk €≥ 10 points

D. (light) manic episode [(HYPO) MANIC EPISODE]

(➔refers to: go to the diagnosis box, circle "No" on the top of the corresponding diagnosis, and then go to the next question group.)

D1	a．	Have you ever had a period of time, feeling ”high mood” or feeling energetic, or confident when you are in trouble, or do others think that you are different when you are peaceful? (Please don’t consider your performance during drinking or drug poisoning.) If the patient is confused about this problem, or is not sure of the meaning of “emotional ups” in your question, explain as follows: I mean “high mood” means: You feel elated, energetic, reduced sleep needs, quick thinking, lots of ideas, increased ability to work, increased creativity, increased initiative, and impulsive behavior.<br>If ” No”, circle ” No” in D1b; if ” Yes”:	No	Yes
	b.	Do you feel ” high mood” or energetic now?	No	Yes
D2	a．	Have you ever had a period of time, a few days are particularly easy to provoke, and therefore often quarrel, or have a verbal dispute or physical attack with people, or shouting at someone other than your family members?<br>Are you or people around you aware that you are more likely to be irritated or overreacted than others? Even if you think there is a reason for the situation (please don’t consider your performance during drinking or drug poisoning)?	No	Yes
		If ” No”, circle ” No” in D2b; if it is:		
		b. Are you still feeling irritated or angry at the moment?	No	Yes
			➔	
		Is D1a or D2a coded ”yes”?	**No**	Yes

D3 *If D1b or D2b = yes: then you only need to ask about the current episode*

If D1b or D2b = no: ask about the situation when the symptoms are most obvious in the past

When you feel ”high mood”, energetic or irritating:

a. Do you feel that you can do something that others can’t do, or are you a particularly important person?	No	Yes
b. Do you need only a small amount of sleep (such as ”Do you feel rested after a few hours of sleep”)?	No	Yes
c. Are you very talkative, difficult to interrupt, or fast, and difficult for others to understand?	No	Yes
d. Are you thinking about problems quickly?	No	Yes
e. Do you feel that your distraction is easy to distract, and any small stimulus can distract you?	No	Yes
f. Are you becoming very active or unable to be quiet, so that others are often worried about you?	No	Yes
g. Are you passionate about engaging in activities that make you feel happy, regardless of risk or consequences (such as spending a lot of time on carnival, driving or sexual activity)?	No	Yes
	➔	
Is there a three or more answer code ”yes” in D3?	No	Yes
Or: D1a = no (previous episode) or D1b = no (current episode), D3 has 4 answer		

code "yes"?

D4 These symptoms last for at least a week and cause obvious problems for your family life, social function or learning, or are you hospitalized because of these symptoms? No Yes

If any answer is "yes", it is rated as "yes"

Is the D4 code "No"?

Please indicate if this is current or past?

No Yes
Manic episode
Current episode €
Past episode€

Is the D4 code "yes"?

Please indicate if this is current or past?

No Yes
Manic episode
Current episode €
Past episode€

E. Panic disorder (PANIC DISORDER)

E1	Have you ever had anxiety, fear, discomfort or nervousness in different situations or situations? Most people in the situation at that time did not feel this way? Does this feeling reach the most serious level in 10 minutes?<br><br>*Only this episode reaches the most severe level within 10 minutes, only the code is ”yes”*	No	Yes
	If E1=No, E5 selects “No” and jumps to F1.		
E2	Have these previous episodes occurred unexpectedly or spontaneously, or are these episodes unpredictable and unpredictable?	No	Yes
	If E2=No, E5 selects “No” and jumps to F1.	No	Yes
E3	After this episode, did you worry about recurrence for more than a month, or worried about the consequences of the episode?	No	Yes
	If E3=No, E5 selects “No” and jumps to F1.		
E4	**During the most serious episodes you can remember, are there the following situations?**		
	a. Do you have a heartbeat, a heartbeat or a heartbeat?	No	Yes
	b. Do you have sweat or wet hands?	No	Yes
	c. Do you have tremors or hand shakes?	No	Yes
	d. Are you breathing or breathing difficulties?	No	Yes
	e. Do you have a feeling of infarction or a foreign body pharynx?	No	Yes
	f. Do you have chest pain, chest pressure or discomfort?	No	Yes
	g. Do you have nausea, stomach upset or sudden diarrhea?	No	Yes
	h. Do you feel dizzy, unstable, top-heavy or faint?	No	Yes
	i. Do you feel that the things around you become strange, unreal, distant or strange, or feel that you are separated from, or completely separated from, part or all of your body?	No	Yes
	j. Are you afraid that you will lose control or go crazy?	No	Yes
	k. Are you afraid that you will die?	No	Yes
	l. Do you have a tingling or numbness in a certain part of your body?	No	Yes
	m. Do you feel flushed or shivering?	No	Yes
E5	*Is the four or more answer in E4 coded ”yes”?*<br>*If E5 = ”No”, skip to E7.*	**No**	**Yes**<br>**Panic disorder lifelong**

E6	**In the past month,** have you repeatedly (three times) this episode, and then always fear of another episode?<br>*If E6 = ”Yes”, skip to F1*	**No Yes**<br><br>**Panic disorder**<br>**Now suffering**
E7	*Is there 1 item, 2 items or 3 items in E4 coded ”Yes”?*	**No Yes**<br>**Partial attack**<br>**lifelong**

F. Location phobia (AGORAPHOBIA)

F1	Do you feel nervous or anxious in certain places or situations, such as places that are difficult to escape or that may not be helpful in the event of a panic attack, such as in a crowd, in a team, or away from home Or stay alone at home, cross the bridge, take a bus, train or car?	No Yes
	If F1=No, F2 selects "No".	
F2	Are you very afraid of these situations, and therefore avoiding these situations, or need to suffer a lot of pain in these situations, or need to be accompanied by others to face?	**No Yes**<br><br>**Place phobia**<br>**Now suffering**
	F2 (Fear of the current situation) code "No"<br>*And*<br>*E6 (now suffering from panic disorder) code "Yes"*	**No Yes**<br>**Panic disorder**<br>**Without fear of place**<br>**Now suffering**
	F2 (Fear of the current situation) code "Yes"<br>*And*<br>*E6 (now suffering from panic disorder) code "Yes"*	**No Yes**<br>**Panic disorder**<br>**Fear of the place**<br>**Now suffering**
	F2 (Fear of the current situation) code "Yes"<br>*And*<br>*E5 (panic disorder, lifetime) code "no"*	**No Yes**<br>**Place phobia**<br>**No history of panic disorder**<br>**Now suffering**

G. Social phobia (social anxiety disorder) [SOCIAL PHOBIA (Social Anxiety Disorder)]

(➔refers to: go to the diagnosis box, circle "No" on the top of the corresponding diagnosis, and then go to the next question group.)

G1	In the past month, when you are being watched by others or become the focus of others' attention, do you feel scared, upset, or afraid of being laughed at/ridiculed? This includes speaking in public, eating in public or eating with others, writing under the eyes of others, or participating in social activities.	➔ No	Yes
G2	Is this fear too much or unreasonable?	➔ No	Yes
G3	Are you very afraid of these situations and avoid them? Or do you feel pain in these situations?	➔ No	Yes
G4	Does this fear undermine your normal work or social function? Or can it cause you obvious pain?	No	Yes

**No**	**Yes**
**Social phobia**	
**Now suffering**	

H. Obsessive-compulsive disorder (OBSESSIVE COMPULSIVE DISORDER)

(➔refers to: go to the diagnosis box, circle "No" on the top of the corresponding diagnosis, and then go to the next question group.)

H1	In the past month, have you been troubled by recurring thoughts, impulses, or images, and are these unpleasant, unpleasant, unpleasant, sudden, or painful? (If you think you are dirty, contaminated, or have bacteria or fear of infecting others, or if you don't want to, but you are afraid of hurting others, or you are afraid to take action under impulse, fear or believe that you will be responsible for something wrong. Responsibility, or ideas, images, or impulses about sexuality, or stubborn thoughts about storing and collecting religion.)<br><br>*Does not include excessive concerns about real life issues.*<br>*Does not include the concept of obsession directly related to eating disorders, sexual preferences, pathological gambling, or alcohol abuse, because patients can get pleasure from these activities, and want to overcome it just because it brings negative results.*	No	Yes
	If H1=No, skip to H4.		
H2	Even when you try to ignore or get rid of them, these ideas will continue to appear in your mind?	No	Yes
	If H2=No, skip to H4.		
H3	Do you think these obsessive ideas are part of your own thoughts, not imposed by the outside world?	No	Yes
H4	In the past month, have you been able to resist something that you can't resist, such as over-washing or cleaning, endlessly checking or counting, or repeating, sorting, placing items, or other superstitious rituals?	No	Yes
	H3 or H4 code "Yes"?	➔<br>No	Yes
H5	Do you realize that these compulsive thinking or compulsions that you are unable to restrain are excessive or unreasonable?	➔<br>No	Yes
H6	Do these compulsive thinking or compulsive behaviors significantly interfere with your normal life, professional function, daily social or relationship, or spend more than an hour a day in this area?	No	Yes
	H6 code "Yes"?	**No**	**Yes**<br>**OCD**<br>**Now suffering**

I. Post-traumatic stress disorder (optional) [POSTTRAUMATIC STRESS DISORDER (optional)]

(➔refers to: go to the diagnosis box, circle "No" on the top of the corresponding diagnosis, and then go to the next question group.)

I1	Have you ever experienced, witnessed, or had to deal with an extremely serious traumatic event, such as a real death, or a threat of death or a serious injury to yourself or others?<br>*(Examples of traumatic events: serious accidents, sexual or physical attacks, terrorist attacks, being taken hostage, kidnapping, robbery, fire, discovery of bodies, accidental death, war, natural disasters...)*	➔<br>No	Yes
I2	In the past month, have you seen this incident painfully (such as nightmares, deep memories, flashbacks, or physiological reactions)?	➔<br>No	Yes
I3	**In the past month:**		
	a. Have you ever avoided thinking about it or avoiding items that remind you of the incident?	No	Yes
	b. Have you ever had a hard time remembering the important part of what happened?	No	Yes
	c. Are you less interested in your original preferences or social activities than before?	No	Yes
	d. Do you feel that you are alienated or unfamiliar with others?	No	Yes
	e. Do you notice that your feelings are numb?	No	Yes
	f. Do you feel that your life will be shortened because of this trauma?	No	Yes
	Is the I3 three or more answer code "Yes"?	➔<br>No	Yes
I4	**In the past month:**		
	a. Do you have difficulty sleeping?	No	Yes
	b. Are you particularly prone to anger or temper?	No	Yes
	c. Do you have difficulty focusing?	No	Yes
	d. Do you feel nervous or often on alert?	No	Yes
	e. Are you vulnerable to fright?	No	Yes
	Is there 2 or more answers in the I4 code "Yes"?	➔<br>No	Yes
I5	In the past month, have these problems significantly interfered with your work or social activities, or caused obvious pain?	No	Yes
	Is the I5 code "yes"?	**No**<br>**Post-traumatic**	**Yes**

Stress disorder
Now suffering

J. Alcohol abuse or alcohol dependence (ALCOHOL ABUSE AND DEPENDENCE)

(➔refers to: go to the diagnosis box, circle "No" on the top of the corresponding diagnosis, and then go to the next question group.)

J1	In the past 12 months, have you ever had more than 3 drinkings, and each time you drink more than 30ml of pure alcohol in 3 hours? For example, 56° white wine is more than two, 32° red wine is about 2 two, 20° wine is 3 or so. It can be combined with local drinking habits or customs.	➔<br>No	Yes
J2	**In the past 12 months**		
	a. Do you need to drink more wine to get the feeling of your initial drink?	No	Yes
	b. Have you ever had hand shaking, sweating or feeling uneasy when you are reducing your alcohol consumption? Or, do you drink to avoid these symptoms or avoid headaches after drinking, such as "hand shake", sweating or restlessness? If one answer is "yes", the code is "yes".	No	Yes
	c. When you drink, do you actually drink more than originally expected?	No	Yes
	d. Have you ever tried to reduce alcohol consumption or stop drinking but did not succeed?	No	Yes
	e. Do you spend a lot of time looking for wine, drinking or sobering during the day of drinking?	No	Yes
	f. Have you reduced your work, your preferences, or the time you spend with others because you drink?	No	Yes
	g. Even if you know that drinking has caused you health or mental problems, continue to drink?	No	Yes

J2 three or more answers code "yes"?

No Yes
Alcohol dependence
Now suffering

Is the patient's alcohol dependence code "yes"?

No ➔ Yes

J3	**In the past 12 months**		
	a. Have you ever been drunk, excited after drinking, or in the aftereffects of alcohol when you study at school, work at work, or do housework at home? What caused this problem?<br>*Only "Yes" is encoded when the problem is caused.*	No	Yes
	b. Have you ever been involved in life-threatening activities such as driving a car, riding a motorcycle, operating a machine, rowing, etc. after being drunk?	No	Yes
	c. Have you ever had an illegal problem with drinking, such as being arrested or harassing law and order?	No	Yes
	d. Even though drinking has already caused problems with your family or other people, do you still drink alcohol?	No	Yes
	J3 has 1 or more than answered "yes"?	**No**	**Yes**<br>**Alcohol abuse**<br>**Now suffering**

K. Non-alcoholic psychoactive substance use disorder (NON-ALCOHOL PSYCHOACTIVE SUBSTANCE USE DISORDERS)

(This question group is mainly for the study of the use of related substances or for the use of high-risk groups. Only the generic name is translated and listed in the original "common name (trade name)". It is recommended that the researcher should address the local situation when needed. Ask the interviewee with the generic name/common name/commodity name.)

(➔refers to: go to the diagnosis box, circle "No" on the top of the corresponding diagnosis, and then go to the next question group.)

K1	Now I want to show you (showing a card for psychoactive substances) or give you a list of drugs or drugs (read the list below). In the past 12 months, have you used more than one of these drugs to achieve the feeling of "floating", making yourself feel better or changing your mood?	➔<br>No	Yes

Circle each drug you have taken:

Stimulants: amphetamines, such as ice, dextroamphetamine, methylphenidate (Ritalin), diet pills.

Cocaine: sonrting, IV, frecbase, crack, "speedball".

Anesthetics: heroin, morphine, hydromorphone hydrochloride, opium, methadone, codeine, compound oxycodone, propoxyphene hydrochloride

Hallucinogen: LSD (acid), mescaline, penicillin, benzocyclidine (PCP) ("Angel Dust", "peace pill", psilocybin, STP, "mushrooms", ecstasy, MDA, or MDMA, Ktamine("special K")

Inhalants: ”glue”, ethyl chloride, ”rush”, nitrous oxide (”laughing gas”), amyl or butyl nitracte (”poppers”).

Cannabis: hashish (”hash”), THC, ”pot”, ”grass”, ”weed”, ”reefer”.

Sedatives: quaalude, Seconal (”reds”), Valium, Xanax, Librium, Ativan, Dalmane, Halcion, Barbiturate, Miltown, GHB, Roofinol, ”roofies”.

Other: steroids, over-the-counter hypnosis, diet pills or cold medicines, etc., any other medicine

Indicate the most used drugs:

- *If multiple substances are used simultaneously or sequentially:*
  - *Each used drug (or a class of drugs)* €
  - *Only investigate the most commonly used drugs (or a class of drugs)* €
- *If only one drug (or one class of drugs) is used:*

Only investigate one drug (or one class of drugs) that has been used €

K2 **Think about your use of the [name of the selected drug or drug category] in the past 12 months]**

a. Did you find that you need to use a larger amount to achieve the effect when you first started using the [name of the drug or drug category]? No Yes

b. When you reduce or stop using the [name of the selected drug or drug category], do you have withdrawal symptoms (pain, hand shake, fever, fatigue, diarrhea, nausea, sweating, palpitations, difficulty sleeping, irritability) , anxiety, irritability or depression)? Or do you use these drugs to avoid discomfort (without symptoms) or to make yourself feel better? No Yes

If any one answers ”yes”, the code is ”yes”.

c. When using the [name of the selected drug or drug category], did you find that the actual amount is more than originally estimated? No Yes

d. Have you ever tried to reduce or stop using the [name of the selected drug or drug category], but did not succeed? No Yes

e. In the days when you used [name of the selected drug or drug category], did you spend a lot of time (> 2 hours) getting the drug, using the drug, or recovering from the effects of the drug or continuing to think about it? No Yes

f. Are you reducing your work, participating in past preferences, or spending time with No Yes

others because of medication?

g. Even if you know that the drug has caused you a health or mental problem, do you still use the [name of the selected drug or drug category]? No Yes

Does K2 have 3 or more answer codes "Yes"? **No Yes Drug dependence Now suffering**

Indicate the drug used:

➔

Is the code for patient drug abuse "yes"? No Yes

K3 **In the past 12 months:**

a. Have you ever used excessive [names of selected drugs or drug classes], excitement after medication, or the effects of drugs in school, school work, or housework at home? What caused this problem? No Yes

Only "Yes" is encoded when the problem is caused.

b. Have you ever engaged in life-threatening activities when you use the [name of the selected drug or drug category] excessively or after excitement? (such as driving a car, riding a motorcycle, operating a machine, rowing, etc.?) No Yes

c. Have you ever had a problem with the use of [name of the selected drug or drug category]? Such as arrested or harassed law and order. No Yes

d. Even if [the name of the selected drug or drug category] has caused problems with your family and other people, do you still use it? No Yes

At least one of the answers in K3 is coded "Yes"? **No Yes**

Drug abuse
Now suffering

Indicate the drug used:

L. Psychiatric disorders (PSYCHOTIC DISORDERS)

(For each question that answered ”yes”, ask for an example. Only when the example given clearly shows a distortion of thought or perception or appears inappropriate in the current cultural context, it is coded ”yes.” Before answering Be careful to evaluate Whether delusions or hallucinations are ”weird”.)

(Weird delusions are: obviously unreasonable, absurd or incomprehensible, and not a delusion derived from everyday life experiences.)

(Weird illusion refers to auditory hallucinations that comment on the patient’s thinking or behavior, or two or more voices talking to each other.)

Now I have to ask you some questions about unusual experiences, others may have experienced:			weird
**L1 a. Have you ever believed that someone is secretly monitoring you, or is someone designing to harm you, or someone wanting to hurt you?**	No	Yes	Yes
If ”Yes”, continue to ask:			
b. Do you still believe in these things now?	No	Yes	Yes ➔ L6a
**L2 a. Have you ever believed that even if you don’t say it, will someone read your thoughts or hear your thoughts? Or have you ever been able to read someone else’s thoughts or hear what others are thinking?**	No	Yes	Yes
If ”Yes”, continue to ask:			
b. Do you still believe in these things now?	No	Yes	Yes ➔ L6a
**L3 a. Have you ever believed that some people or the outside world have some kind of ideas that are not your own, input into your brain, or forced you to act in ways that are not your usual way? You used to feel yourself Is it attached?**	No	Yes	Yes
If ”Yes”, continue to ask:			
b. Do you still believe in these things now?	No	Yes	Yes ➔ L6a
**L4 a. Have you ever believed that TV, radio or newspaper is broadcasting special messages to you? Or do people you don’t know pay special attention to you?**	No	Yes	Yes
If ”Yes”, continue to ask:			
b. Do you still believe in these things now?	No	Yes	Yes ➔ L6a

L5 a. Did your relatives or friends ever think that your thoughts were strange or unusual? No Yes Yes

(Excluding the delusions asked about the L1 to L4 questions, such as exaggerating swearing, suspecting illness, destroying sinful delusions, etc., only coding "yes".)

If the answer is "Yes", continue to ask:

b. Do they still think that your idea is strange? No Yes Yes

No Yes Yes

L6 a. Have you ever heard a voice that no one else can hear?

(Only when the patient answers "yes" to the following question, the code is weird "yes". Is the voice you hear commenting on your thoughts or behavior, or are you hearing two or more voices talking to each other?

If "Yes", continue to ask:

b. Can you hear these sounds in the last month? No Yes Is L8b➔

L7 a. Have you seen something special when you are awake, or see something that others can't see? No Yes

(If the vision is not suitable for the current cultural background, the code is "yes")

If "Yes", continue to ask:

b. Can you still see this in the last month? No Yes

Interviewer judgment

L8 b. Does the patient currently exhibit incoherent speech, speech disorder, or significant thought relaxation? No Yes

L9 b. Does the patient currently exhibit behavioral disorder or stupor? No Yes

L10 b. During the interview, did the patient show significant negative symptoms of schizophrenia? No Yes

Such as: obvious apathy, lack of speech, or the inability to initiate and maintain purposeful actions (will decline)

**L11**	*From L1 to L10:*<br>*Have 1 or more "b" questions answer "weird"?*<br>***or***<br>*There are 2 or more "b" questions to answer "yes", but not "weird"?*	**No Yes**<br>**Psychiatric disorder**<br>**Now suffering**
**L12**	*From L1 to L7*<br>*Answer 1 "weird" code with 1 or more "a" questions;*<br>***or***<br>*Have 2 or more "a" questions answered "yes" but not "weird"?*<br>*(Need to confirm whether these two symptoms occurred in the same period of time in the past)*<br>***or***<br>***Is the L11 code "Yes"?***	**No Yes**<br>**Psychiatric disorder**<br>**lifelong**
**L13**	a. If the L12 code is "Yes", or if L1-L7 has at least 1 "Yes", does the patient meet any of the following diagnoses?<br>*Depression (current or previous episode)*<br>***or***<br>*Manic episode (current or previous episode)*	➔<br>No Yes
	As you said before, you have felt for a while (depression/emotional up/sustained irritability).	
	b. These beliefs and experiences you just mentioned (the symptoms of the L1-L7 code " yes ") only occur when you feel depressed/emotional/sustainable irritability?	no Yes
	Is the L13b code "yes"?	**No Yes**<br>**Mood disorder**<br>**Psychiatric symptoms**<br>**Now suffering**

M. Anorexia nervosa (ANOREXIA NERVOSA)

(➔refers to: go to the diagnosis box, circle "No" on the top of the corresponding diagnosis, and then go to the next question group.)

M1 a What's your height? ☐ ☐ ☐ cm

b What is your minimum weight in the past three months? ☐ ☐ ☐ kg

c Is the patient's weight lower than the lower body weight corresponding to his height? ➔ No Yes

(See the reference standard at the bottom of this page)

In the past three months:

M2 Even though your weight is so low, are you still trying not to gain weight? ➔ No Yes

M3 Even though your weight is already low, are you still afraid of gaining weight or getting fat? ➔ No Yes

M4 a Do you think you are too fat, or is some part of your body too fat? No Yes

b Does your weight or body shape seriously affect how you feel about yourself? No Yes

c Do you think the current low weight status is normal or even too fat? No Yes

M5 ***In the question of M4, is one or more of the answers coded "yes"?*** ➔ No Yes

M6 Only for female patients: Has there been menopause in the past three months? (At the time you were not pregnant) ➔ No Yes

Female patients: Is the M5 and M6 code "yes"?
Male patient: Does the M5 code "yes"?

**No** **Yes**
**Anorexia nervosa**
**Now suffering**

Table: Adult standard height/body weight (weight) reference threshold (height - no shoes; body weight - no clothes)

Height (cm)	145	150	155	160	165	170	175	180	185	190
Body mass (kg)	37	39	42	45	48	51	54	57	60	64

Note: The body mass threshold in the table is the calculated value, and the body mass is not less than 15%

of the normal body mass (as defined by DSM-IV).

N. Neurogenic bulimia (BULIMIA NERVOSA)

(➔refers to: go to the diagnosis box, circle "No" on the top of the corresponding diagnosis, and then go to the next question group.)

**N1**	In the past three months, have you ever gluttoned or consumed too much food within two hours?	➔ No	Yes
**N2**	In the past three months, have you had as many as 2 binge eatings per week?	➔ No	Yes
**N3**	Do you feel that your food is uncontrollable when you are overeating?	➔ No	Yes
**N4**	In order to avoid weight gain after binge eating, have you taken compensation actions such as: vomiting, fasting, exercising, taking laxatives, enema, diuretics (aqueous medications) or other medications?	➔ No	Yes
**N5**	Does your weight or body shape seriously affect how you feel about yourself?	➔ No	Yes
**N6**	Does the patient's symptoms meet the diagnostic criteria for anorexia nervosa?	No	Yes
	If N6 = "No", skip to N8.		
**N7**	Does this binge eating only happen when you weigh less than (kg)*?<br>** Refer to the standard height and weight of the anorexia nervosa group, and record the standard weight in accordance with the patient's height in parentheses.*	No	Yes
**N8**	***N5 encoding "Yes", and N7 encoding "No" or being skipped?***	**No Yes**<br>**Neurogenic bulimia**<br>**Now suffering**	
	Is the N7 code "yes"?	**No Yes**<br>**Anorexia nervosa**<br>**Gluttony/clearing type**	

Now suffering

O. GENERALIZED ANXIETY DISORDER

(➔refers to: go to the diagnosis box, circle "No" on the top of the corresponding diagnosis, and then go to the next question group.)

**O1**	**a In the past six months, have you been overly worried or nervous about a series of events in your daily life, at work, at home, or around you?**<br><br>*If the patient's anxiety is due to a disease we have previously assessed, such as a panic attack (panic disorder), anxiety in the public (social anxiety disorder), fear of being contaminated (obsessive-compulsive disorder), or fear of weight gain (nervous anorexia)... , then no longer encode "yes" here.*	➔<br>No	Yes
	**b Are you worried about most days?**	➔<br>No	Yes
**O2**	Have you found it difficult to control these concerns, or have these concerns interfered with you so that you can't concentrate on doing your own thing?	➔<br>No	Yes
**O3**	*From O3a to O3f, if these symptoms are caused by a disease of the previous question group, the code is "No".*<br>**In the past six months when you feel anxious, you almost every day:**		
	**Does it feel restless, "full of strings" or on the verge of losing control?**	No	Yes
	**Does b feel muscle tension?**	No	Yes
	**Does c feel tired, tired or exhausted?**	No	Yes
	**d Is it difficult to concentrate or feel a blank brain?**	No	Yes
	**Does e feel irritating?**	No	Yes
	**f Is there a sleep disorder (difficult to fall asleep, wake up at night, wake up early or sleep too much)?**	No	Yes

Does O3 have 3 or more answer codes "Yes"?

No Yes
Generalized anxiety disorder
Now suffering

P. Antisocial personality disorder (optional)
[ANTISOCIAL PERSONALITY DISORDER (optional)]

(➔refers to: go to the diagnosis box, circle "No" on the top of the corresponding diagnosis, and then go to the next question group.)

		No	Yes
**P1**	**Before the age of 15, did you:**		
	a often skip school or run out of home all night?	No	Yes
	b often lie, lie, or steal things?	No	Yes
	c provoking fights, bullying, intimidating or provoking others?	No	Yes
	d intentionally damaged items or arson?	No	Yes
	e intentionally cruel or harm others?	No	Yes
	f Force others to have sex with you?	No	Yes
	***P1** has **2** or more answer codes "Yes"?*	➔ No	Yes
**P2**	*The following behavior, if only for political or religious motives, does not encode "yes"*		
	**From the age of 15, do you:**		
	**a** often acts in a way that makes others think that you are irresponsible, such as taking something without giving money, deliberately showing impulsiveness or deliberately not working, unable to support yourself?	No	Yes
	**b** Do something illegal, even if you are not caught (eg destroying property, picking up a sheep at a store, stealing something, selling drugs, or committing a felony?)	No	Yes
	**c** often fight with people (including physical conflicts with spouses or children)?	No	Yes
	**d** often lie or deceive others to obtain other people's property or pleasure, or just to deceive for fun?	No	Yes
	**e** to put others in a dangerous situation regardless of?	No	Yes
	**f** hurt, abuse others, lie, steal other people's things, or damage other people's property, there is no guilt (guilty) feeling?	No	Yes
	***P2** has **3** or more answer codes "Yes"?*	**No**	**Yes**

No Yes
Antisocial personality disorder lifelong

End of interview

Please fill in the M.I.N.I diagnostic record
