# Supplementary material for: A phase 3, multicenter, double-blind, randomized, placebo-controlled clinical trial to verify the efficacy and safety of ansofaxine (LY03005) for major depressive disorder
Source: Transl Psychiatry. 2023 May 10;13:163. doi: 10.1038/s41398-023-02435-0 (PMC10171157; doi:10.1038/s41398-023-02435-0)
Supplement: Supplementary file 2 — Supplementary Information [file 41398_2023_2435_MOESM2_ESM.pdf]

Supplementary Information

Supplementary Fig 1. The Changes from Baseline in $HAMD_{17}$ Total Score (FAS)

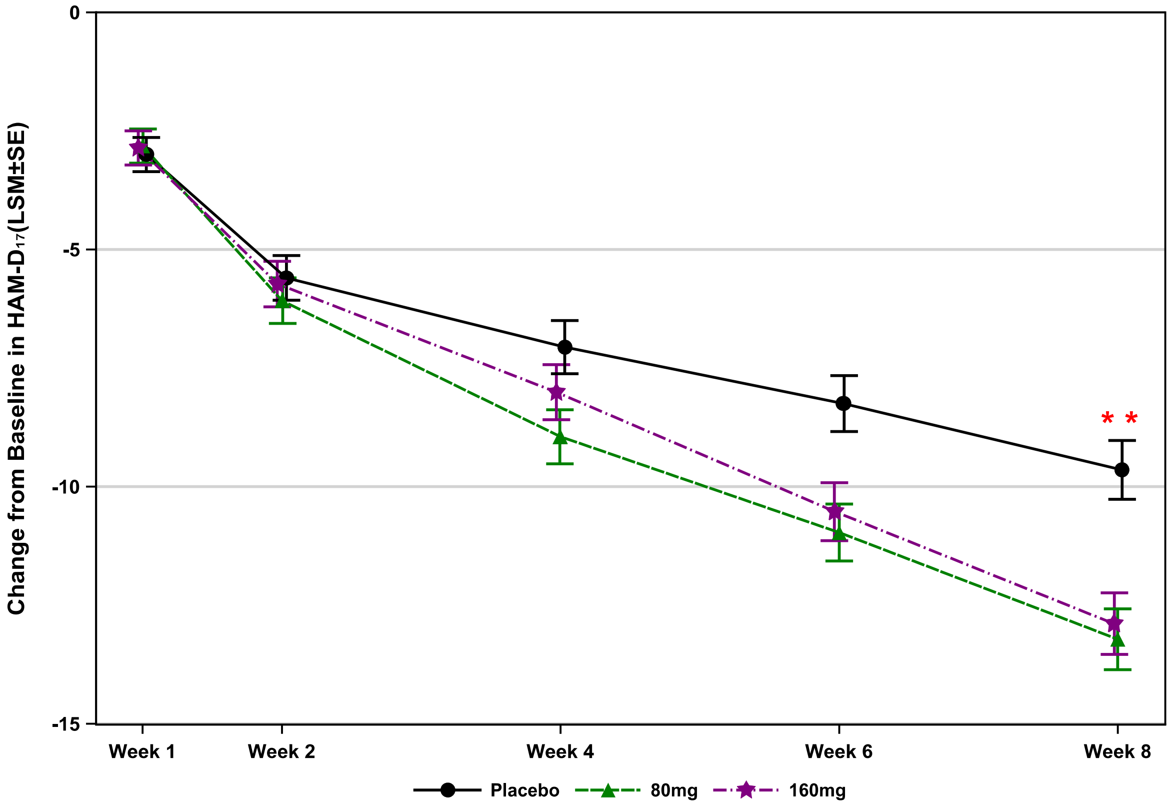

** indicates P < 0.0001. FAS, full analysis set; $HAM\text{-}D_{17}$, 17-item Hamilton Depression Rating Scale.

Supplementary Fig 2. MADRS Response and Remission rate at the end of week 8

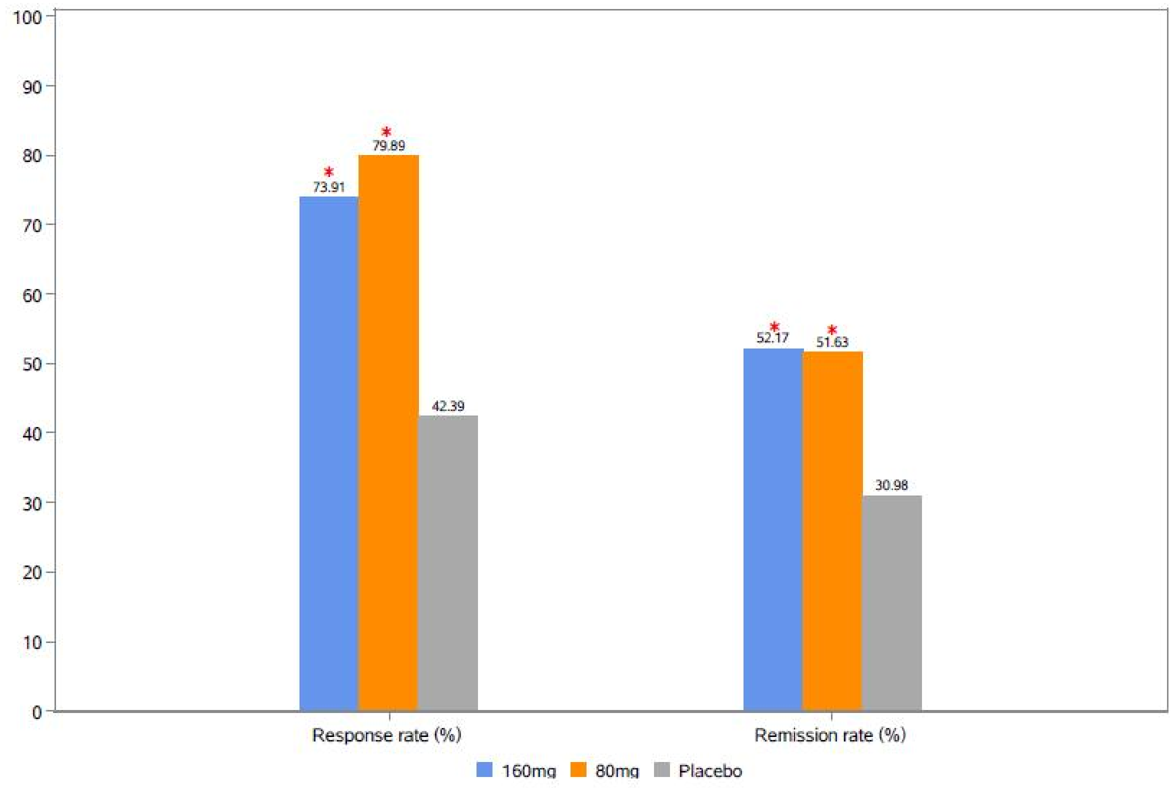

* Indicates significant changes *vs.* placebo.
MADRS, Montgomery- Åsberg Depression Scale.

Supplementary Fig 3. HAM-D17 Response and Remission rate at the end of week 8

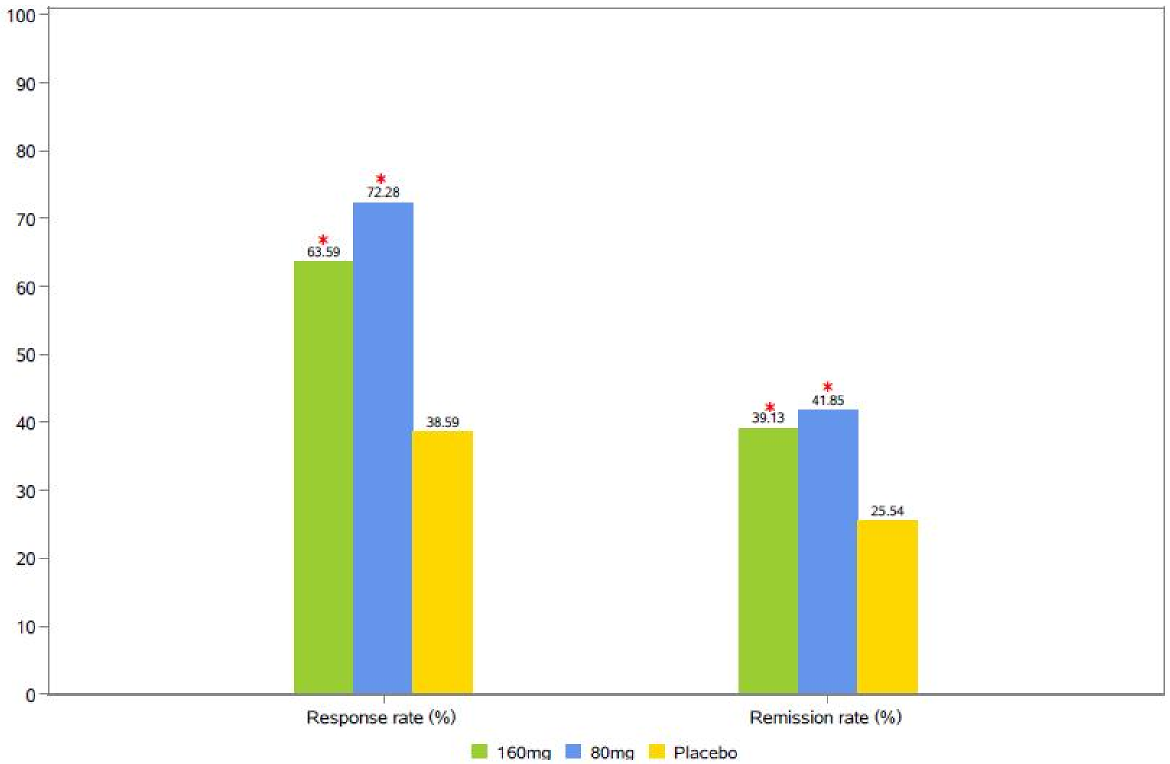

* Indicates significant changes *vs.* placebo.
HAMD17, 17-item Hamilton Depression Rating Scale.

Safety

Vital signs

There were no significant changes in armpit temperature or changes in respiration rate or changes in pulse after treatment intra- and inter-group.

Supine and orthostatic blood pressure

Ansofaxine ER tablets might slightly increase the mean (±SD) changes of supine systolic and diastolic pressure in 160 mg group *vs.* placebo. The baseline mean systolic pressure in supine position were 110.95±11.316 mmHg, 109.07±10.569 mmHg, and 110.36±10.956 mmHg in 80 mg, 160 mg and placebo group respectively. The LS mean changes of supine systolic pressure post 8-week treatment *vs.* baseline were 0.92±8.500 mmHg, 2.96±9.008 mmHg and -0.98±7.652 mmHg in 80 mg, 160 mg, and placebo group respectively. The baseline mean diastolic pressure in supine position were 70.60±8.109 mmHg, 69.03 ± 7.347 mmHg and 69.27±7.621 mmHg in 80 mg, 160 mg and placebo group respectively. The mean changes of supine diastolic pressure post 8-week treatment *vs.* baseline were 1.58±7.151 mmHg, 3.86±6.791 mmHg and 0.27±7.082 mmHg in 80 mg, 160 mg, and placebo group respectively (see Table S1).

The mean (±SD) changes of orthostatic systolic and diastolic pressure in 80 mg and 160 mg of ansofaxine ER tablets were similar as that of placebo group.

Table.S1 Changes of Mean Supine Systolic and Diastolic Pressure Post 8-week Treatment *vs.* Baseline

	160mg	80mg	Placebo
**Supine Systolic Pressure**			
Baseline			
N (Missing)	184 (0)	184 (0)	184 (0)
Mean (SD)	109.07 (10.569)	110.95 (11.316)	110.36 (10.956)
8-week treatment			
N (Missing)	146 (38)	148 (36)	151 (33)
Mean (SD)	111.43 (9.674)	111.36 (10.359)	109.92 (9.723)
8-week treatment -Baseline			
N (Missing)	146 (38)	148 (36)	151 (33)
Mean (SD)	2.96 (9.008)	0.92 (8.500)	-0.98 (7.652)
LS Mean (95%CI)	4.418 (2.836, 5.999)	3.415 (1.852, 4.978)	1.873 (0.319, 3.427)
LSM changes *vs.* placebo (95%CI)	2.544(0.939,4.149)	1.542(-0.050. 3.133)	
**Supine Diastolic Pressure**			
Baseline			
N (Missing)	184 (0)	184 (0)	184 (0)
Mean (SD)	69.03 (7.347)	70.60 (8.109)	69.27 (7.621)
8-week treatment			
N (Missing)	146 (38)	148 (36)	151 (33)
Mean (SD)	72.62 (7.855)	71.78 (7.927)	69.80 (8.008)
8-week treatment -Baseline			
N (Missing)	146 (38)	148 (36)	151 (33)
Mean (SD)	3.86 (6.791)	1.58 (7.151)	0.27 (7.082)
LS Mean (95%CI)	5.639 (4.254, 7.024)	3.980 (2.611, 5.349)	2.446 (1.105, 3.788)
LSM changes *vs.* placebo (95%CI)	3.193 (1.794, 4.592)	1.534 (0.138, 2.930)	

Physical examination

Physical examination included lungs, abdomen, spine, limbs, thyroid gland, lymph node, skin mucosa, nervous

system, head and neck, facial features, heart, general condition, motor system, and others. During the study period, there was two cases in 160 mg of ansofaxine ER tablets, who had normal skin mucosa on the baseline and became abnormal after 8 weeks of treatment, presenting as a superficial knife scar on the wrist and scabbed several knife scratches on the left wrist, respectively. Both has reported adverse events and was confirmed to be unlikely related and unrelated to the study drug, respectively.

Laboratory tests

Blood routine: Except some individuals were abnormal with clinical significance, blood routine didn't show any significant trend with time changes. Treatment-emergent adverse events of blood routine were listed in table S2. All the TEAEs were mild or moderate.

Table S2. Treatment-Emergent Adverse Events of Blood Routine

	160 mg (N=184)			80 mg (N=184)			Placebo (N=184)		
**Preferred Term**	**Events**	**Cases**	**Incidence (%)**	**Events**	**Cases**	**Incidence (%)**	**Events**	**Cases**	**Incidence (%)**
White blood cell count increased	2	2	1.09	0	0	0.00	2	2	1.09
White blood cell count decreased	1	1	0.54	0	0	0.00	3	3	1.63
Platelet count increased	0	0	0.00	0	0	0.00	2	2	1.09
Plateletcrit increased	0	0	0.00	0	0	0.00	1	1	0.54
Neutrophil count decreased	0	0	0.00	0	0	0.00	1	1	0.54
Granulocytopenia	2	2	1.09	0	0	0	0	0	0
Anaemia	2	2	1.09	0	0	0	1	1	0.54

Blood biochemistry: Blood biochemistry didn't show any significant trend with time changes. Treatment-emergent adverse events of blood biochemistry were listed in table S3 and all the TEAEs were mild or moderate.

Table S3. Treatment-Emergent Adverse Events of Blood Biochemistry

	160 mg (N=184)			80 mg (N=184)			Placebo (N=184)		
**Preferred Term**	**Events**	**Cases**	**Incidence (%)**	**Events**	**Cases**	**Incidence (%)**	**Events**	**Cases**	**Incidence (%)**
Blood triglycerides increased	3	3	1.63	0	0	0.00	3	3	1.63
Aspartate aminotransferase increased	2	2	1.09	0	0	0.00	1	1	0.54
Blood bilirubin increased	3	2	1.09	0	0	0.00	2	2	1.09
Blood creatine phosphokinase abnormal	2	2	1.09	0	0	0.00	1	1	0.54
Blood glucose increased	2	2	1.09	0	0	0.00	1	1	0.54
Gamma-glutamyltransferase increased	1	1	0.54	1	1	0.54	0	0	0.00
Low density lipoprotein increased	1	1	0.54	0	0	0.00	1	1	0.54
High density lipoprotein increased	1	1	0.54	0	0	0.00	0	0	0.00
Bilirubin conjugated increased	1	1	0.54	0	0	0.00	1	1	0.54
Blood cholesterol increased	1	1	0.54	0	0	0.00	1	1	0.54
Blood creatine phosphokinase MB increased	1	1	0.54	0	0	0.00	0	0	0.00
Blood creatine phosphokinase increased	1	1	0.54	0	0	0.00	1	1	0.54
Blood potassium decreased	1	1	0.54	1	1	0.54	0	0	0.00
Blood urea increased	1	1	0.54	0	0	0.00	0	0	0.00
Blood uric acid abnormal	1	1	0.54	0	0	0.00	0	0	0.00
Apolipoprotein B increased	1	1	0.54	0	0	0.00	0	0	0.00
Alanine aminotransferase increased	0	0	0.00	2	2	1.09	1	1	0.54
Myocardial necrosis marker increased	0	0	0.00	0	0	0.00	1	1	0.54
Blood albumin increased	0	0	0.00	0	0	0.00	1	1	0.54
Blood potassium abnormal	0	0	0.00	0	0	0.00	1	1	0.54
Blood chloride decreased	0	0	0.00	0	0	0.00	1	1	0.54
Protein total increased	0	0	0.00	0	0	0.00	1	1	0.54
Dyslipidaemia	3	3	1.63	0	0	0.00	0	0	0.00
Hypertriglyceridaemia	1	1	0.54	0	0	0.00	0	0	0.00
Hyperuricaemia	1	1	0.54	1	1	0.54	0	0	0.00
Hypokalaemia	0	0	0.00	1	1	0.54	0	0	0.00
Hyperlipidaemia	0	0	0.00	1	1	0.54	1	1	0.54
Rhabdomyolysis	1	1	0.54	0	0	0.00	0	0	0.00
Renal impairment	0	0	0.00	1	1	0.54	0	0	0.00
Hepatic function abnormal	2	2	1.09	0	0	0.00	3	3	1.63

Serum prolactin: Serum prolactin didn't show any significant trend with time changes. Treatment-emergent adverse events of serum prolactin were listed in table S4 and all the TEAEs were mild or moderate.

Table S4. Treatment-Emergent Adverse Events of Serum Prolactin

	160 mg (N=184)			80 mg (N=184)			Placebo (N=184)		
**Preferred Term**	**Events**	**Cases**	**Incidence (%)**	**Events**	**Cases**	**Incidence (%)**	**Events**	**Cases**	**Incidence (%)**
Blood prolactin increased	5	5	2.72	6	6	3.26	1	1	0.54
Blood prolactin decreased	0	0	0.00	1	1	0.54	0	0	0.00
Hyperprolactinaemia	0	0	0.00	0	0	0.00	2	2	1.09

Urinalysis： Urinalysis didn't show any significant trend with time changes. Treatment-emergent adverse events of urinalysis were listed in table S5 and all the TEAEs were mild or moderate.

Table S5. Treatment-Emergent Adverse Events of Urinalysis

	160 mg (N=184)			80 mg (N=184)			Placebo (N=184)		
**Preferred Term**	**Events**	**Cases**	**Incidence (%)**	**Events**	**Cases**	**Incidence (%)**	**Events**	**Cases**	**Incidence (%)**
Urobilinogen urine increased	7	6	3.26	3	3	1.63	1	1	0.54
White blood cells urine positive	5	5	2.72	2	2	1.09	6	6	3.26
Protein urine present	6	5	2.72	2	2	1.09	4	4	2.17
Urine ketone body present	5	5	2.72	3	3	1.63	2	2	1.09
Urinary occult blood positive	4	4	2.17	3	3	1.63	1	1	0.54
Urine bilirubin increased	2	2	1.09	1	1	0.54	0	0	0.00
Red blood cells urine positive	1	1	0.54	0	0	0.00	1	1	0.54
Urinary tract infection	3	3	1.63	2	2	1.09	2	2	1.09
Proteinuria	0	0	0.00	0	0	0.00	1	1	0.54

12-Lead ECG

In this study, the baseline means (±SD) of heart rates (HR) were 69.4±10.67 bpm, 69.2±10.52 bpm and 68.9±11.20 bpm in ansofaxine 80 mg, ansofaxine 160 mg and placebo group respectively. After 8-week treatment, the mean (±SD) changes of heart rates (HR) *vs.* baseline were 0.9±9.94bpm, 4.6±10.32 bpm and -1.7±9.99 bpm in ansofaxine 80 mg, ansofaxine 160 mg and placebo group respectively. Ansofaxine 160 mg demonstrated a slight increase of the HR in the comparison between 8 weeks and baseline whereas there were no obvious changes in the HR in ansofaxine 80 mg and placebo group.

The baseline means (±SD) of corrected Q-T interval (QTc) were 402.08±22.367 ms, 403.28±22.456 ms and 402.30±24.354 ms in ansofaxine 80 mg, ansofaxine 160 mg and placebo group respectively. After 8-week treatment, the mean (±SD) changes of QTc *vs.* baseline were 2.66±18.203 ms, 1.19±18.225 ms and -0.05±19.806 ms in ansofaxine 80 mg, ansofaxine 160 mg and placebo group respectively. There were no obvious changes in the QTc in ansofaxine 80 mg, ansofaxine 160 mg and placebo group.

The other parameters of 12-Lead ECG didn't show any significant trend with time changes.

C-SSRS

Ansofaxine ER tablets (80 mg and 160 mg) didn't increased suicide risks in comparison with placebo group. One case in 80 mg group reported a TEAE named suicidal thinking, which was considered unlikely related to the studied drug by the investigator.
